# A cytidine deaminase biodegrader sensitises multiple cancer types to deoxycytidine analogue-based chemotherapies

**DOI:** 10.1038/s41467-026-78315-0

**Published:** 2026-10-07

**Authors:** Marie Sorbara, Margot Cristol, Charlène Lopez, Ben Allal, Manon Gervais, Anaïs Faihy, Margaux Vienne, Naïma Hanoun, Constance Manso, Alexandre Stella, Laetitia Ligat, Odile Burlet-Schiltz, Etienne Chatelut, Laura Poillet, Carine Joffre, Fabienne Thomas, Pierre Cordelier, Nicolas Bery

**Affiliations:** 1 Université de Toulouse, CNRS, Inserm, CRCT, Toulouse, France; 2 Université de Toulouse, Oncopole-Claudius Regaud, IUCT-Oncopole, Inserm, CRCT, Toulouse, France; 3 Université de Toulouse, CNRS, IPBS, Toulouse, France; 4 Infrastructure Nationale de Protéomique, ProFI, Toulouse, France; 5 Service de gastroentérologie et de pancréatologie, CHU de Toulouse, Université de Toulouse, Toulouse, France

**Keywords:** Protein design, Cancer therapeutic resistance, Drug development

## Abstract

Deoxycytidine (dC) analogues are widely used to treat solid tumours and blood cancers. However, their clinical efficacy is often limited by resistance mediated by cytidine deaminase (CDA), which converts these drugs into inactive metabolites. Existing CDA inhibitors have limited efficacy and off-target effects, highlighting the need for alternative approaches. Here, we show the development of a CDA biodegrader, an intracellular anti-CDA VHH fused to the SPOP E3 ubiquitin ligase, enabling specific CDA degradation. It effectively depletes CDA in pancreatic, lung, and acute myeloid leukaemia cancer cell lines, increasing sensitivity to dC analogues in vitro. Combined with gemcitabine, the CDA biodegrader inhibits pancreatic and lung tumour growth in vivo. Importantly, mRNA encoding the biodegrader delivered using virus-like particles achieves efficient sensitisation to gemcitabine in vitro and in vivo. This proof-of-concept study establishes targeted CDA degradation as a feasible strategy to enhance the therapeutic efficacy of dC analogues.

## Abstract (web summary)

Deoxycytidine (dC) analogues form the basis of many chemotherapeutic agents but their efficacy is limited by cytidine deaminase-mediated inactivation. Here, the authors develop an intracellular biodegrader targeting cytidine deaminase, capable of sensitising multiple cancer types to dC analogues.

## Introduction

Despite significant progress in cancer treatment over the past decades, resistance to both classical chemotherapeutic agents and novel targeted drugs remains a major challenge. Drug resistance is, in fact, responsible for most cancer relapses and is a leading cause of cancer-related death^1^. Deoxycytidine (dC) analogues, such as gemcitabine and cytarabine, belong to the antimetabolites class of chemotherapeutic agents, used in the treatment of various cancers^2^. These compounds function as prodrugs that require cellular uptake and subsequent intracellular activation to exert their cytotoxic effects^3^. Gemcitabine is one of the chemotherapies used for patients with pancreatic cancer^4^ and is also administered to patients with lung cancer combined with a platinum-based chemotherapy agent^5^. Cytarabine, also known as Ara-C, is one of the major chemotherapy drugs used in combination with an anthracycline in acute myeloid leukaemia (AML)^6^. Their efficacy, however, is limited by resistance mechanisms that notably impair cellular uptake or target the nucleotide metabolism enzymes required for their activation^2^. Cytidine deaminase (CDA) has been identified as one key regulator of resistance to these therapies once they reach the cells, promoting their deamination and subsequent inactivation^7^. Actually, up to 90% of gemcitabine can be inactivated by CDA^8^. CDA is an enzyme from the pyrimidine salvage pathway that catalyses the transformation of cytidine and deoxycytidine into uridine and deoxyuridine, respectively^9^. It participates in maintaining the nucleotides pool for DNA/RNA biosynthesis, complementary to the de novo pyrimidine synthesis pathway^9^. Importantly, CDA is not an essential enzyme as Cda^-/-^ mice are viable and have a comparable lifetime than Cda^+/+^ mice^10^. Small molecule inhibitors targeting CDA active site have been developed but exhibit limited efficacy^11^ and/or off-target effects^12,13^. Hence, to target CDA and overcome the resistance to dC analogues, alternative strategies need to be explored.

Intracellular antibody reagents, i.e. antibodies expressed inside the cells, are one appealing avenue to fulfil this objective. We and others demonstrated that the high affinity and specificity of intracellular antibodies can be exploited to modulate the function of proteins that are hard-to-target by conventional approaches (e.g. RNA/DNA-based techniques, small molecules) such as protein-protein interactions (PPIs)^14–16^, post-translational modifications^17,18^ or specific conformations^19–21^. To enhance the potency of intracellular antibodies, they can be functionalised with an E3 ubiquitin ligase domain to induce target degradation via the ubiquitin-proteasome system^19,22–25^. They are known as bioPROTACs or biodegraders^26^. The targeted degradation method offers several advantages: (i) it enhances protein interference through a catalytic mechanism of action, where a single biodegrader molecule can induce the degradation of multiple target protein molecules; and (ii) it only requires binding to the target, regardless of whether the intracellular antibody inhibits its function^27^. However, like most intracellular proteins, biodegraders cannot penetrate cell membranes, which hinders their use as direct therapeutics. Only a few studies have reported successful delivery of intracellular biodegraders in vitro and/or in vivo, either by directly administering modified biodegrader proteins conjugated with cell-penetrating peptides (CPP)^23^, encapsulating them in lipid nanoparticles (LNPs)^28^ or nanogel^29^, delivering mRNA via LNPs^30^ or cationic peptides^31^, or inserting biodegrader cDNA into adeno-associated viruses (AAV) genome^25,32^. Therefore, no systematic delivery approaches has yet been established for biodegraders.

Here, we report the identification of a CDA biodegrader through a cell-based screen. This biodegrader is highly specific and potently depletes CDA across a panel of cancer cell lines, including pancreatic, lung, and AML. CDA degradation sensitises pancreatic and lung cancer cells to gemcitabine in both in vitro and in vivo settings. The CDA biodegrader further sensitises AML cells to another dC analogue, cytarabine, in vitro. Importantly, CDA biodegrader mRNA delivered via non-integrative virus-like particles (VLPs) using the FlashRNA® vector effectively sensitises pancreatic cancer cells to gemcitabine in vitro and lung cancer cells in both in vitro and in vivo models. These findings reveal that the CDA biodegrader represents a promising and feasible strategy to improve the efficacy of dC analogues in cancer therapy.

## Results

### A cell-based screening identifies intracellular CDA biodegraders

A biodegrader is made of an E3 ligase domain that is fused to a protein binder specific of a target, here an intracellular antibody. We previously showed that this fusion does not always produce a functional biodegrader^22,33^. We therefore established a two-steps workflow to identify functional biodegraders targeting CDA: (i) the selection of a functional E3 ligase and (ii) the identification of a functional biodegrader using that E3 ligase. To achieve that, we first created HeLa S3 cells stably expressing GFP-CDA. We then tested the ability of previously reported GFP biodegraders^34^ to decrease the GFP signal in HeLa S3/GFP-CDA cells compared to their related negative control (i.e. anti-ALFA tag biodegraders^34^). A mCherry fluorescent transfection reporter is included on each biodegrader construct, which enables GFP intensity quantification in the mCherry positive cells. Flow cytometry data showed that the SPOP-based GFP biodegrader is, among the three tested, the most potent to reduce the GFP fluorescence intensity of HeLa S3/GFP-CDA cell line (Supplementary Fig. 1a, b). SPOP is therefore the E3 ligase that we selected to fuse to anti-CDA variable heavy domain of heavy chain-only antibody (VHHs).

**Fig. 1:**
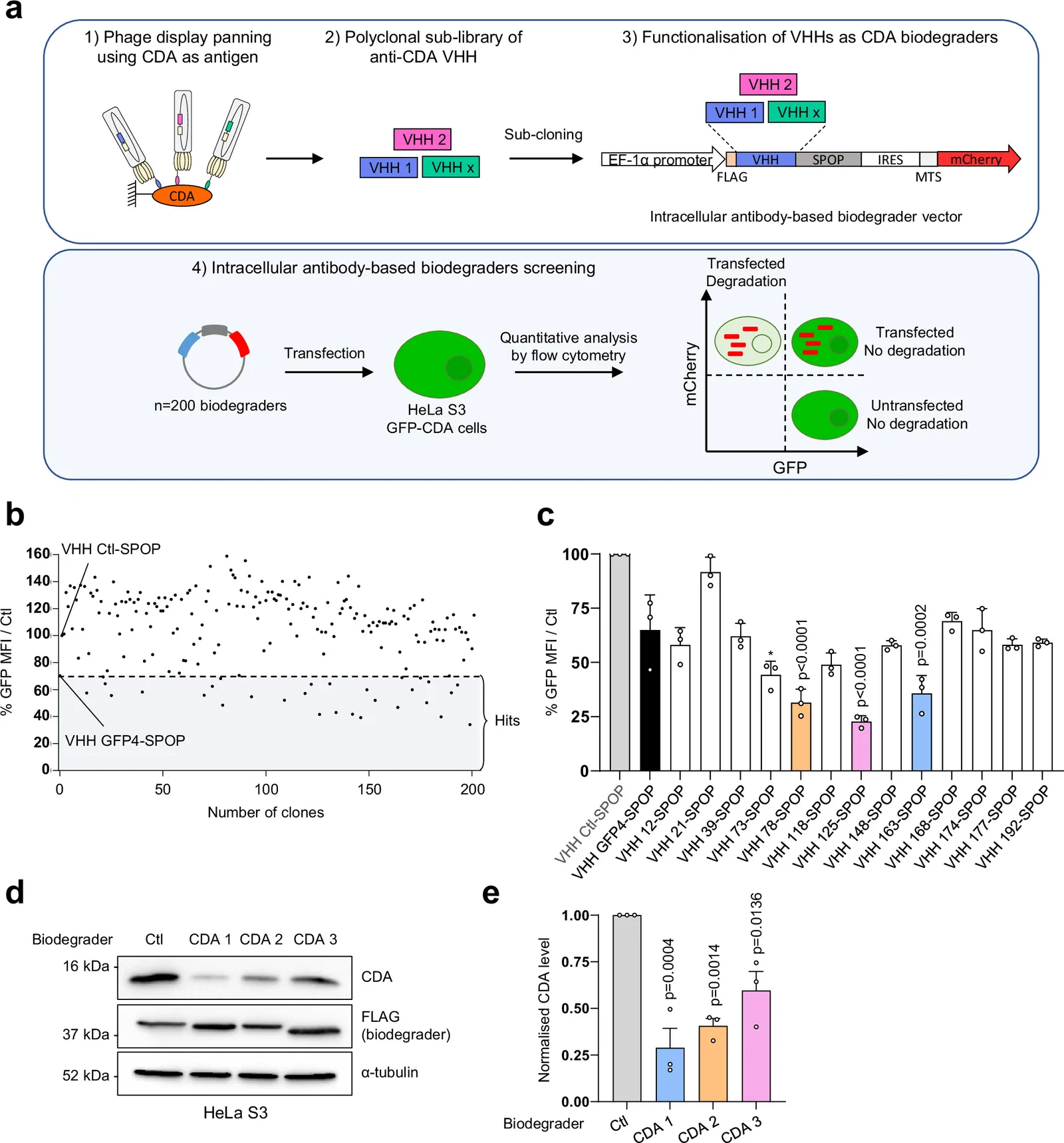
A cell-based screening for intracellular biodegraders identifies CDA biodegraders. **a** General scheme of intracellular antibody-based biodegraders selection (by Power Point). (1) Phage display was performed in vitro to select anti-CDA VHHs. (2) A polyclonal sub-library of anti-CDA VHHs was obtained after 4 rounds of phage display by digestion of the DNA coding for the VHHs. (3) Digested VHHs cDNA were directly subcloned in fusion with SPOP E3 ligase domain in the antibody-based biodegraders vector that includes a fluorescent transfection reporter (mCherry mitochondria). (4) The cell-based screening was performed by transient transfection of 200 plasmids coding for biodegraders in HeLa S3 cells stably expressing GFP-CDA. GFP-CDA degradation was quantified by flow cytometry by measuring GFP intensity in the transfected cells (mCherry positive cells). **b** Scatter plot of the GFP median fluorescence intensity (MFI) of each transfected biodegrader (mCherry positive cells) measured by flow cytometry and normalised to the GFP MFI of the negative control (VHH ALFA-SPOP hereafter named VHH Ctl-SPOP) in HeLa S3/GFP-CDA. The positivity threshold (dotted line) is set on the level of the positive control (VHH GFP4-SPOP). **c** Confirmation of the positive hits assessed by flow cytometry. GFP MFI of HeLa S3/GFP-CDA transfected for 48 h by the 13 positives hits of the screening normalised as in (**b**). **d** Western blot analysis of the endogenously expressed CDA degradation by the biodegraders. The three best biodegraders from the screening (hereafter named CDA biodegraders 1, 2 and 3) were transfected 48 h in HeLa S3 cells along with a control biodegrader (Ctl biodegrader) and CDA expression was determined by Western blotting. The biodegraders expression is detected with a FLAG antibody. α-tubulin is the loading control. **e** CDA level quantification from (**d**). Statistical significance in (**c**, **e**) was determined by one-way ANOVA followed by Dunnett post-hoc tests (ns, not significant). Error bars in (**c**) correspond to mean values ± standard deviation (SD) or ± standard mean of error (SEM) in (**e**) from three independent biological repeats. Each experiment was performed once (**b**) or three times (**c**–**e**) as biological repeats. Source data are provided as a Source Data file.

To isolate anti-CDA binders, we performed a phage display panning against CDA by employing NaLi-H1 synthetic VHH library^33^ (Fig. 1a). After four rounds of panning, we obtained a polyclonal sub-library of VHHs enriched towards CDA. We directly subcloned their cDNA in fusion to SPOP E3 ligase in a bicistronic biodegrader vector^35^ (Fig. 1a). To identify functional CDA biodegraders, i.e. that deplete CDA protein inside the cells, we performed a cell-based screening by flow cytometry. We transiently transfected individually 200 plasmids encoding for biodegraders in HeLa S3/GFP-CDA cells. 48 h later, we measured the GFP median of fluorescence intensity (MFI) of the transfected -mCherry positive - cells (Fig. 1a). We identified 30 hits that decreased the GFP MFI at a level comparable to that of SPOP-based GFP biodegrader, the positive control (Fig. 1b). DNA sequencing analysis of the 30 hits showed 13 different biodegrader clones (Supplementary Fig. 1c). Of note, one hit sequence (clone 163) was repeated 10 times, highlighting its enrichments during the phage display selection process. We then confirmed the hits positivity by repeating the flow cytometry experiment with the 13 different biodegraders in HeLa S3/GFP-CDA cells (Fig. 1c) and in HeLa S3/H2B-GFP cells (Supplementary Fig. 1d). None of the biodegraders diminished the H2B-GFP signal (Supplementary Fig. 1d) and only VHH21-SPOP did not reduce the GFP-CDA MFI compared to the positive control (Fig. 1c), demonstrating the specificity of the 12 remaining hits for GFP-CDA. We next focused on the three best CDA biodegraders, i.e. which induced the highest decrease of GFP-CDA MFI: VHH 78-SPOP (orange), VHH 125-SPOP (pink), and VHH 163-SPOP (blue). We characterised these three biodegraders and first assessed whether their activity was driven by SPOP/VHH fusion and not by the expression of the VHH itself. 3xFLAG-VHHs were not able to reduce GFP-CDA intensity compared to their respective biodegrader, confirming that the degradation is dependent on the fusion with SPOP (Supplementary Fig. 1e). Additionally, we determined whether GFP-CDA degradation was dependent on the proteasome activity. Treatment of cells expressing CDA biodegraders with epoxomicin, a proteasome inhibitor, impeded the decrease of GFP-CDA MFI (Supplementary Fig. 1f). Finally, we evaluated the efficacy of CDA biodegraders to deplete endogenously expressed, and untagged, CDA in HeLa S3 cells. Ctl and CDA biodegraders 1, 2 and 3 (VHH 163, 78 and 125 fused to SPOP, respectively) were transiently transfected in HeLa S3 cells for 48 h. Western blotting analysis revealed that CDA was depleted by the three biodegraders with different degradation efficiencies (Fig. 1d, e). CDA biodegrader 1 induced the strongest depletion of CDA, followed by CDA biodegrader 2 and 3 (Fig. 1e). Therefore, we decided to pursue the study with the two most efficient CDA biodegraders 1 and 2.

### Anti-CDA VHHs bind with high affinity and specificity to their target

We next thought to characterise the binding of the VHHs targeting CDA (anti-CDA VHH 1 and 2 derived from CDA biodegraders 1 and 2, respectively). We first monitored the interaction of the VHHs with CDA in live cells by Bioluminescence Resonance Energy Transfer (BRET) donor saturation assay (Supplementary Fig. 2a). BRET data showed that both VHHs interacted specifically with CDA WT, but also with CDA K27Q, the most described non-synonymous single-nucleotide polymorphism (SNP) found in patients^36^, compared to the Ctl VHH (Supplementary Fig. 2a). Importantly, in these conditions, anti-CDA VHHs 1 and 2, CDA WT and CDA K27Q were equally expressed in the cells (Supplementary Fig. 2a). Furthermore, BRET experiments demonstrated that anti-CDA VHHs 1 and 2 did not interact with Activated Induced CDA (AID, Supplementary Fig. 2b), which shares a similar deamination mechanism and has an active site structurally close to that of CDA^37^. The BRET data suggest a specificity of these antibodies towards CDA. We next confirmed that these VHHs bind CDA overexpressed in HEK293T cells (Supplementary Fig. 2c) and endogenously expressed in HeLa S3 cells (Supplementary Fig. 2d) by co-immunoprecipitation. We noticed that VHH CDA 1 precipitated more CDA protein than VHH CDA 2, while being expressed at the same level (Supplementary Fig. 2c, d). These data suggest that VHH CDA 1 has a stronger affinity than VHH CDA 2. To verify this hypothesis, the affinity of each VHH was measured by Surface Plasmon Resonance method (SPR). SPR results showed that anti-CDA VHHs 1 and 2 have a high affinity for their target with a *K*_D_ in the pM and nM range, respectively (Supplementary Fig. 2e). These findings confirm that VHH CDA 1 has a stronger affinity towards CDA than VHH CDA 2.

### CDA biodegraders enable a rapid, long-lasting and specific CDA degradation

**Fig. 2:**
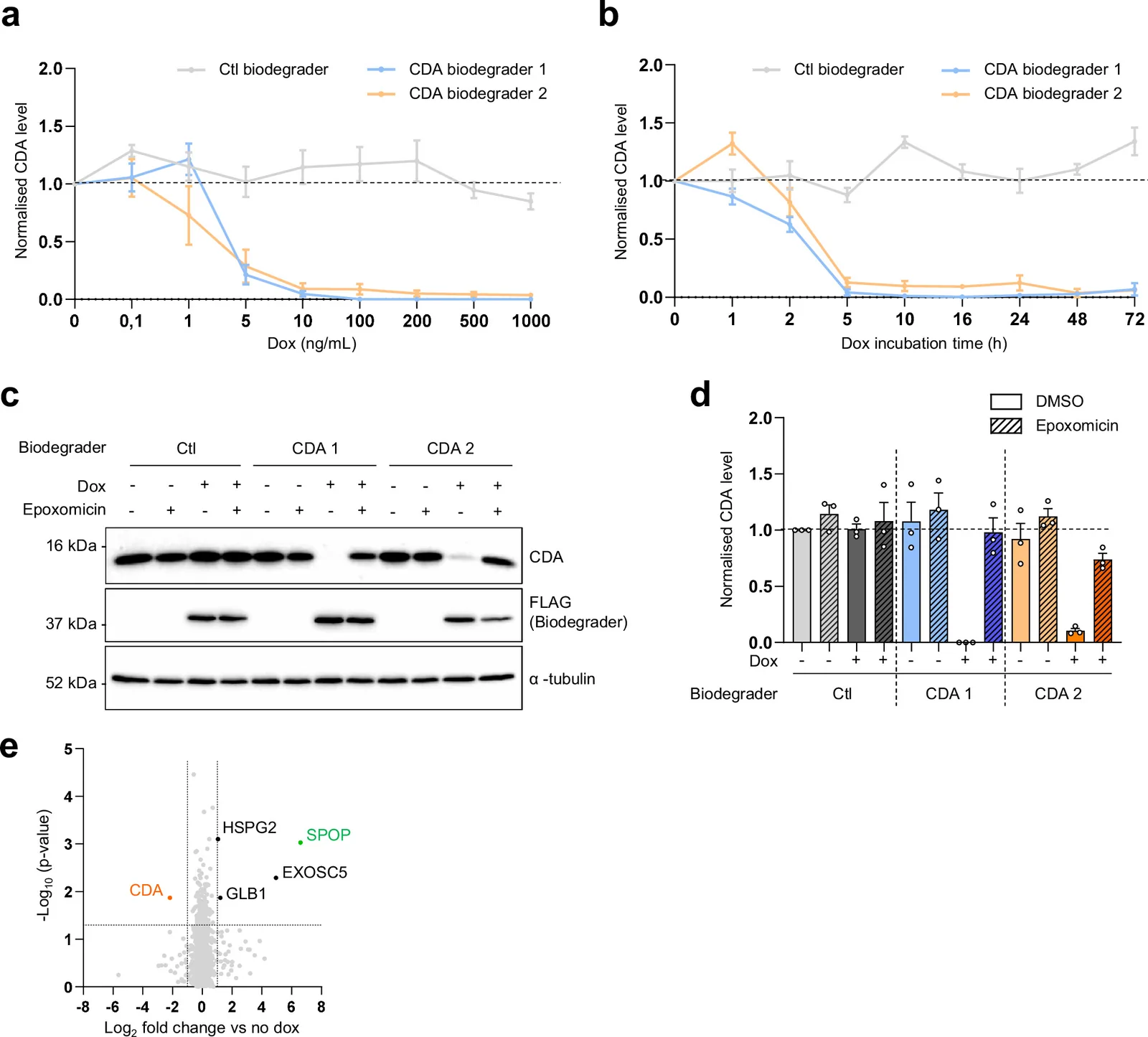
CDA biodegraders induce a rapid, sustained and specific degradation of CDA. Characterisation of CDA biodegraders in AsPC-1 pancreatic cancer cell line. **a** Quantification of CDA protein level from the dose response experiments with the indicated doxycycline (dox) concentrations incubated 48 h in AsPC-1 cells expressing either Ctl, CDA biodegraders 1 or 2. CDA protein level is normalised to the no dox condition for each biodegrader. **b** Quantification of CDA protein level from the time response experiments after addition of 0.5 µg.mL^−1^ of dox in AsPC-1 cells. For each biodegrader, CDA protein level is normalised to the no dox condition. **c** AsPC1 cells expressing ( + dox) or not (- dox) the different biodegraders were treated with DMSO or epoxomicin (0.8 μM), a proteasome inhibitor epoxomicin (crossed bars) for 18 h. CDA protein level was evaluated by Western blotting. **d** Quantification of CDA protein level from (**c**) normalised to the untreated control condition. **e** Whole cell proteomics mass spectrometry analysis of AsPC-1 cells expressing CDA biodegrader 1 (10 h of dox treatment at 0.5 µg.mL^−1^) or not (no dox). *P*-values were determined by a two-tailed unpaired Student’s *t* test. Significantly altered proteins (*P* < 0.05, log_2_ fold change < −1 or >1) are indicated as black dots, CDA is in orange and SPOP in green on the volcano plot. Error bars in (**a**, **b**, **d**) are mean ± SEM of biological repeats. Each experiment was performed with three independent biological replicates (**a**–**e**). Source data are provided as a Source Data file.

Next, we aimed to characterise CDA biodegraders 1 and 2 properties compared to the Ctl biodegrader in AsPC-1, a pancreatic cancer cell line that expresses the enzyme. Target cells were transduced with a lentivector using the TET-ON inducible system, i.e. biodegraders expression is induced after doxycycline treatment. We first performed a dose response of doxycycline to assess the specificity of degradation of the biodegraders as shown by transient transfection in Fig. 1e. We analysed the expression of the biodegraders and CDA by Western blots (Supplementary Fig. 3a, quantified in Fig. 2a), which demonstrated a dose-dependent depletion of CDA with CDA biodegraders 1 and 2 only and not with the Ctl biodegrader (Fig. 2a and Supplementary Fig. 3a). We then studied CDA degradation kinetics. The biodegraders protein expression was detected as early as 5 h after doxycycline induction and persisted up to 72 h, which was associated with a complete degradation of CDA (Supplementary Fig. 3b and Fig. 2b). We confirmed that CDA degradation only occurred at the protein level, as CDA mRNA levels were not affected in AsPC-1 cells expressing or not the biodegraders (Supplementary Fig. 3c). Then, we assessed the requirement of the proteasome activity for CDA degradation with epoxomicin treatment. We showed that epoxomicin inhibited CDA depletion by CDA biodegraders 1 and 2 (Fig. 2c, d). Finally, we evaluated CDA biodegrader specificity by analysing changes in the proteome of AsPC-1 cells expressing or not the CDA biodegrader 1 by liquid chromatography tandem mass spectrometry (LC-MS/MS). MS analysis revealed that CDA is the only significantly detected protein showing >50% depletion (*P* < 0.05) and that SPOP is the most enriched protein after 10 h of CDA biodegrader 1 expression (Fig. 2e and Supplementary Fig. 3d). In addition, no proteins were significantly downregulated when the Ctl biodegrader was expressed in the same conditions (Supplementary Fig. 3d, e). Of note, in addition to the upregulation of SPOP for both conditions, three and four different proteins were significantly increased upon expression of CDA or Ctl biodegraders, respectively (Fig. 2e and Supplementary Fig. 3e), ruling out a potential off-target effect of the doxycycline itself. Overall, CDA biodegraders induce a rapid, long-lasting and highly specific degradation of CDA in cancer cells.

### CDA biodegraders induce CDA degradation in cancer cell lines

**Fig. 3:**
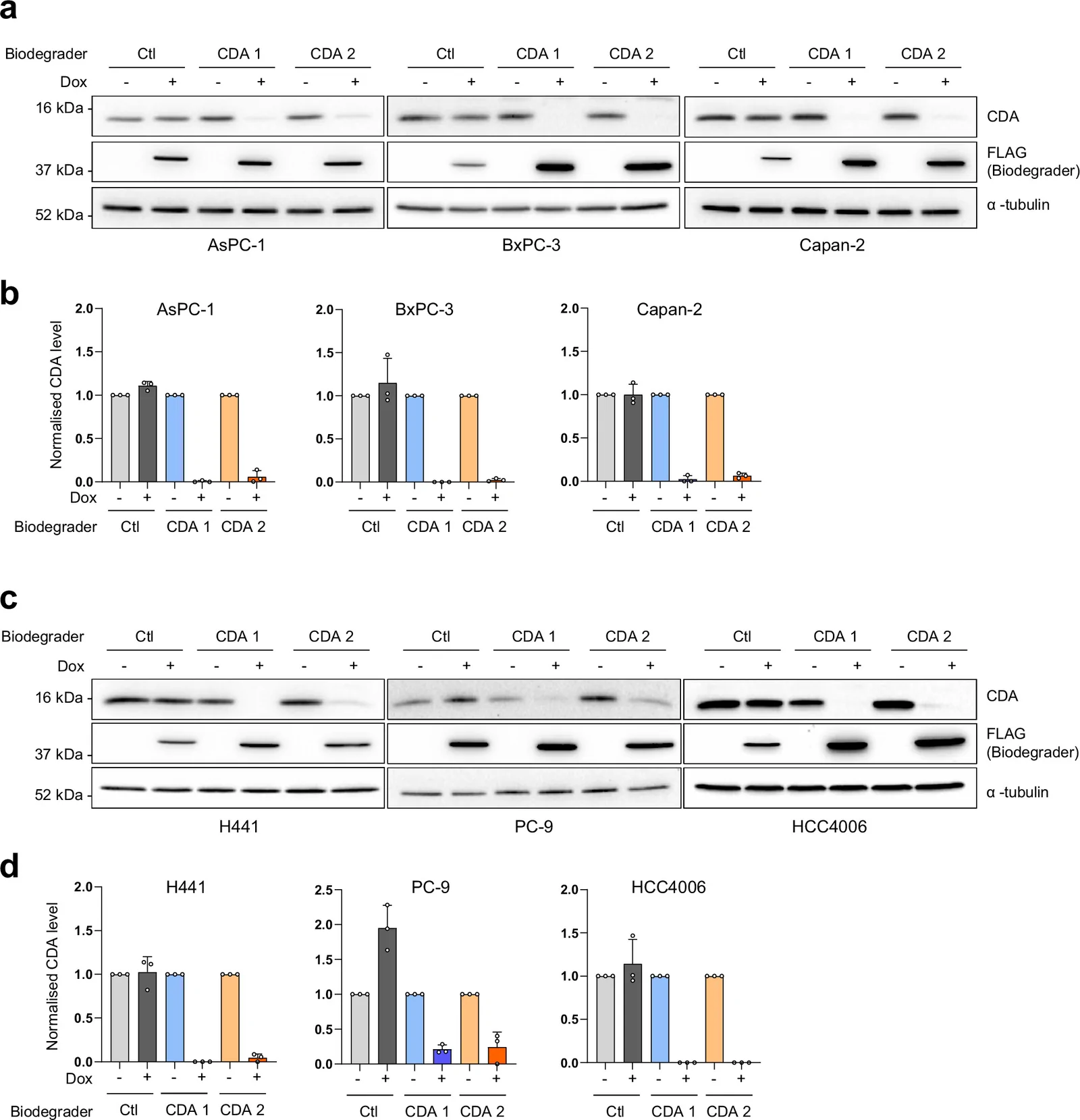
CDA biodegraders efficiently deplete CDA in cancer cells. Western blots of CDA level upon induction of the biodegraders across pancreatic and lung cancer cell lines. **a** Efficacy of CDA biodegraders 1 and 2 to deplete CDA in AsPC-1, BxPC-3 and Capan-2 pancreatic cancer cell lines after 48 h of dox induction compared to the Ctl biodegrader. **b** Quantification of CDA protein level from (**a**) normalised to the no dox (-) condition of each biodegrader. **c** Efficacy of CDA biodegraders 1 and 2 to degrade CDA in lung cancer cell lines H441, PC-9 and HCC4006 after 48 h of dox induction compared to the Ctl biodegrader. **d** Quantification of CDA protein level from (**c**) normalised to the no dox (-) condition of each biodegrader. Biodegraders expression is shown with the FLAG antibody. α-tubulin is the loading control. Error bars are mean ± SD (**b**, **d**) of biological repeats. Each experiment was performed independently three times as biological replicates (**a**–**d**). Source data are provided as a Source Data file.

CDA protein is heterogeneously overexpressed in pancreatic and lung tumours compared to adjacent normal tissues^38,39^. To evaluate the efficacy of CDA biodegraders across different pancreatic and lung cancer cell lines, we first assessed CDA protein expression levels by Western blot analysis. In pancreatic cancer cell lines, Capan-2 and AsPC-1 expressed higher level of CDA than BxPC-3 (Supplementary Fig. 4a). On the contrary, MIA PaCa-2 cells did not express detectable level of CDA and will be used as control cells (Supplementary Fig. 4a). In lung cancer, H441 is the cell line that expressed the highest amount of CDA, followed by PC-9 and HCC4006 (Supplementary Fig. 4b). Importantly, we also included in this panel two non-cancerous lung cell lines BEAS-2B (epithelial cells) and MRC-5 (fibroblasts) as controls and they did not express detectable levels of CDA (Supplementary Fig. 4b). We then assessed Ctl/CDA biodegraders efficacy in these different cell lines by lentiviral transduction using a TET-ON inducible system, as previously performed with AsPC-1 cells (Fig. 2). After 48 h of doxycycline induction, we analysed the expression levels of biodegraders and CDA by Western blotting. As shown for AsPC-1 cells (Fig. 2c and Supplementary Fig. 3a, b, d), the Ctl biodegrader was expressed and did not affect CDA expression (Fig. 3a–d). Both CDA biodegraders depleted CDA in all pancreatic cancer cell lines expressing CDA (Fig. 3a, quantification in Fig. 3b) as well as in lung cancer cells (Fig. 3c, quantification in Fig. 3d), although CDA biodegrader 1 was more potent than CDA biodegrader 2 in depleting CDA. To evaluate the impact of CDA biodegraders 1 and 2 in cells that have no detectable level of CDA, we generated MIA PaCa-2, BEAS-2B and MRC-5 inducible stable cell lines. Our results indicated that the biodegraders were expressed after doxycycline addition, while CDA was still not detected (Supplementary Fig. 4c). Altogether, these data show that CDA biodegraders effectively depleted CDA in various pancreatic and lung cancer cell lines.

### CDA biodegrader chemosensitises pancreatic and lung cancer cells to gemcitabine in vitro

**Fig. 4:**
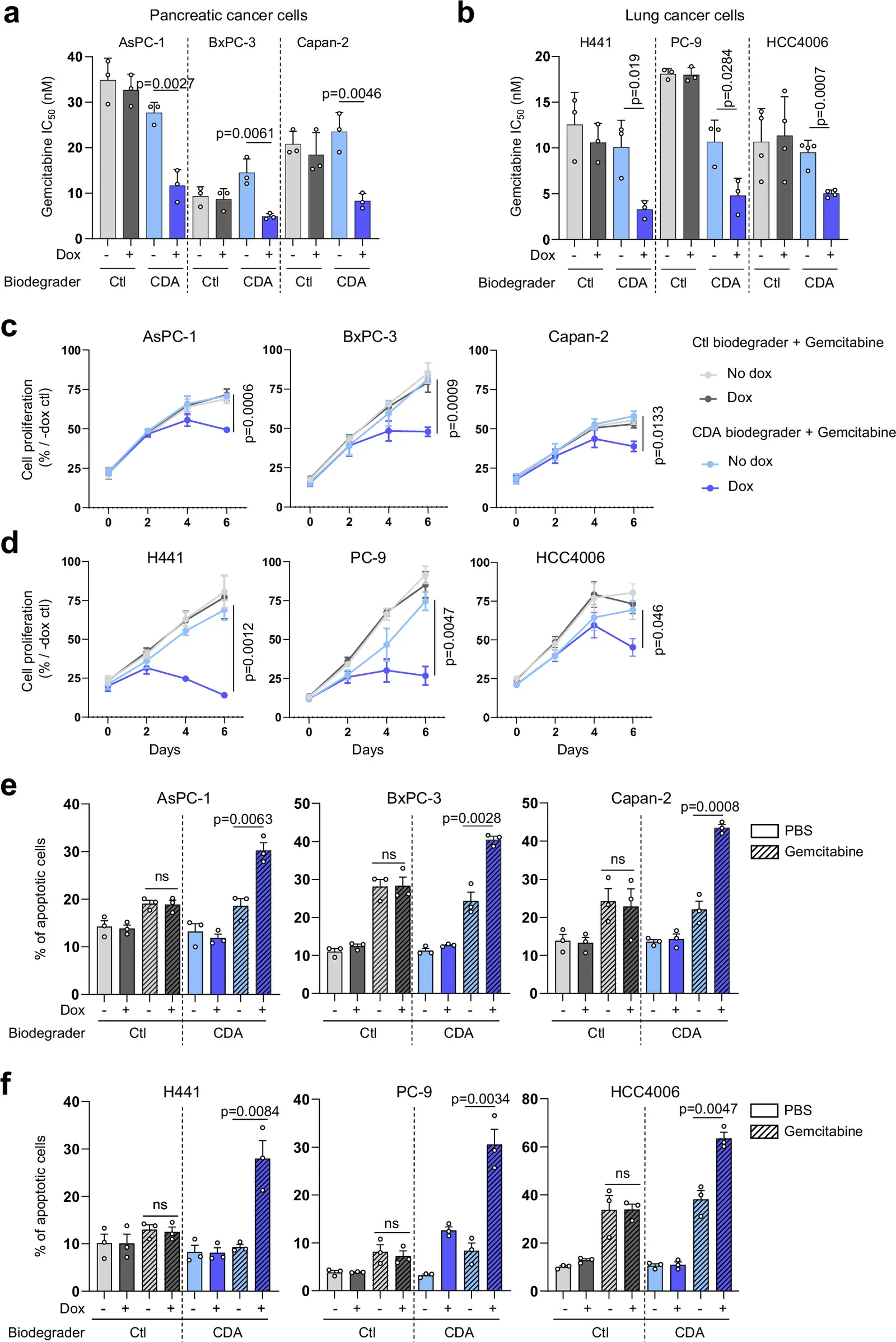
CDA biodegrader sensitises pancreatic and lung cancer cells to gemcitabine in vitro. **a** Pancreatic cancer cells (AsPC-1, BxPC-3 and Capan-2) and **b** lung cancer cells (H441, PC-9 and HCC4006) expressing either the Ctl or CDA biodegrader ( + dox) or not (- dox) were treated for 6 days with a range of gemcitabine concentrations. The half-maximal inhibitory concentration (IC_50_) values of gemcitabine were determined at day 6 with the Cell Titre Glo assay for each cell line. Cell proliferation of AsPC-1, BxPC-3 and Capan-2 cell lines (**c**) or H441, PC-9 and HCC4006 cell lines (**d**) expressing either the Ctl or CDA biodegrader (dox) or not (no dox) and treated with gemcitabine for 6 days at the IC_50_ value of 25 nM, 10 nM, and 20 nM respectively for pancreatic cancer cell lines and 10 nM for lung cancer cell lines. Percentage of apoptotic cells determined by Annexin V/Propidium Iodide (PI) staining of AsPC-1, BxPC-3 and Capan-2 cell lines (**e**) and H441, PC-9 and HCC4006 cell lines (**f**) expressing either the Ctl or CDA biodegrader ( + dox) or not (- dox) and treated (crossed bars) or not with gemcitabine at the IC_50_ value for 6 days. The data are displayed as percentage of the Annexin V positive/Propidium Iodide negative cell population from the total cell population after flow cytometry analysis. Statistical significance was determined by a two-tailed unpaired Student’s t-test in (**a**–**f**). ns not significant. Error bars indicate mean ± SD (**a**, **b**) or SEM (**c**–**f**) of biological repeats. Each experiment was performed three times in (**a**–**f**), excepted HCC4006 in (**b**) (*n* = 4), as biological replicates. Source data are provided as a Source Data file.

We next evaluated whether CDA degradation would sensitise these cells to gemcitabine in vitro. Because CDA biodegrader 1 is more potent than CDA biodegrader 2 at depleting CDA, we decided to perform the following experiments using this biodegrader (hereafter named CDA biodegrader). To this end, we determined gemcitabine half-maximal inhibitory concentration (IC_50_) across all cancer cell lines by assessing cell viability following 6 days of treatment with doxycycline and/or gemcitabine. As expected, the Ctl biodegrader had no effect on gemcitabine IC_50_ in any tested cell line (Fig. 4a, b and Supplementary Fig. 5a). In contrast, CDA biodegrader significantly reduced gemcitabine IC_50_ values in all CDA-expressing cell lines (Fig. 4a, b), but not in cells with undetectable CDA level (Supplementary Fig. 5a), supporting that the measured sensitisation is driven by targeted CDA degradation. We then assessed whether CDA biodegrader would directly impact gemcitabine metabolism by reducing its metabolic inactivation through CDA degradation. To this end, we quantified the inactive gemcitabine metabolite 2’,2’-difluorodeoxyuridine (dFdU) level in AsPC-1 cells supernatant treated with gemcitabine and with or without doxycycline to induce the CDA biodegrader expression using Ultra Performance Liquid Chromatography (UPLC)-MS/MS. dFdU level is significantly decreased upon CDA biodegrader expression (Supplementary Fig. 5b, c), concomitant with CDA depletion (Supplementary Fig. 5c), suggesting that CDA biodegrader positively affects gemcitabine metabolism. We next examined the effect of CDA degradation, with or without gemcitabine treatment, on the proliferation of pancreatic and lung cancer cell lines. The combination of CDA biodegrader and gemcitabine significantly inhibited cell proliferation compared to gemcitabine treatment alone, while the Ctl biodegrader had no effect (Fig. 4c, d). Interestingly, 6 days of CDA biodegrader expression did not impact the viability of cells with undetectable level of CDA (Supplementary Fig. 5d). We further analysed the impact of CDA biodegrader alone on the proliferation of CDA-positive cells. In the absence of gemcitabine, CDA biodegrader had no effect on most cell lines, with the exception of a significant reduction in proliferation observed in PC-9 and AsPC−1 cells after 6 days of its expression (Supplementary Fig. 5e, f). To investigate deeper the molecular mechanisms underlying the proliferation inhibition observed in Fig. 4c, d, we analysed the percentage of apoptotic cells by flow cytometry (Fig. 4e, f and Supplementary Fig. 5g) and the expression of apoptosis protein markers by Western blot analyses (Supplementary Fig. 5h, i). Flow cytometry analysis revealed a significant increase in apoptotic cells upon expression of CDA biodegrader in combination with gemcitabine treatment, compared to gemcitabine alone (Fig. 4e, f). Accordingly, the expression of the apoptosis markers cleaved caspase 3 (CL-CAS3) and cleaved PARP (CL-PARP) was increased in all cell lines with the combination CDA biodegrader/gemcitabine, compared to gemcitabine alone (Supplementary Fig. 5h, i). Overall, our data imply that targeted degradation of CDA sensitises cancer cells to gemcitabine, leading to reduced cell proliferation through increased apoptosis in vitro.

### CDA biodegrader chemosensitises AML to cytarabine in vitro

**Fig. 5:**
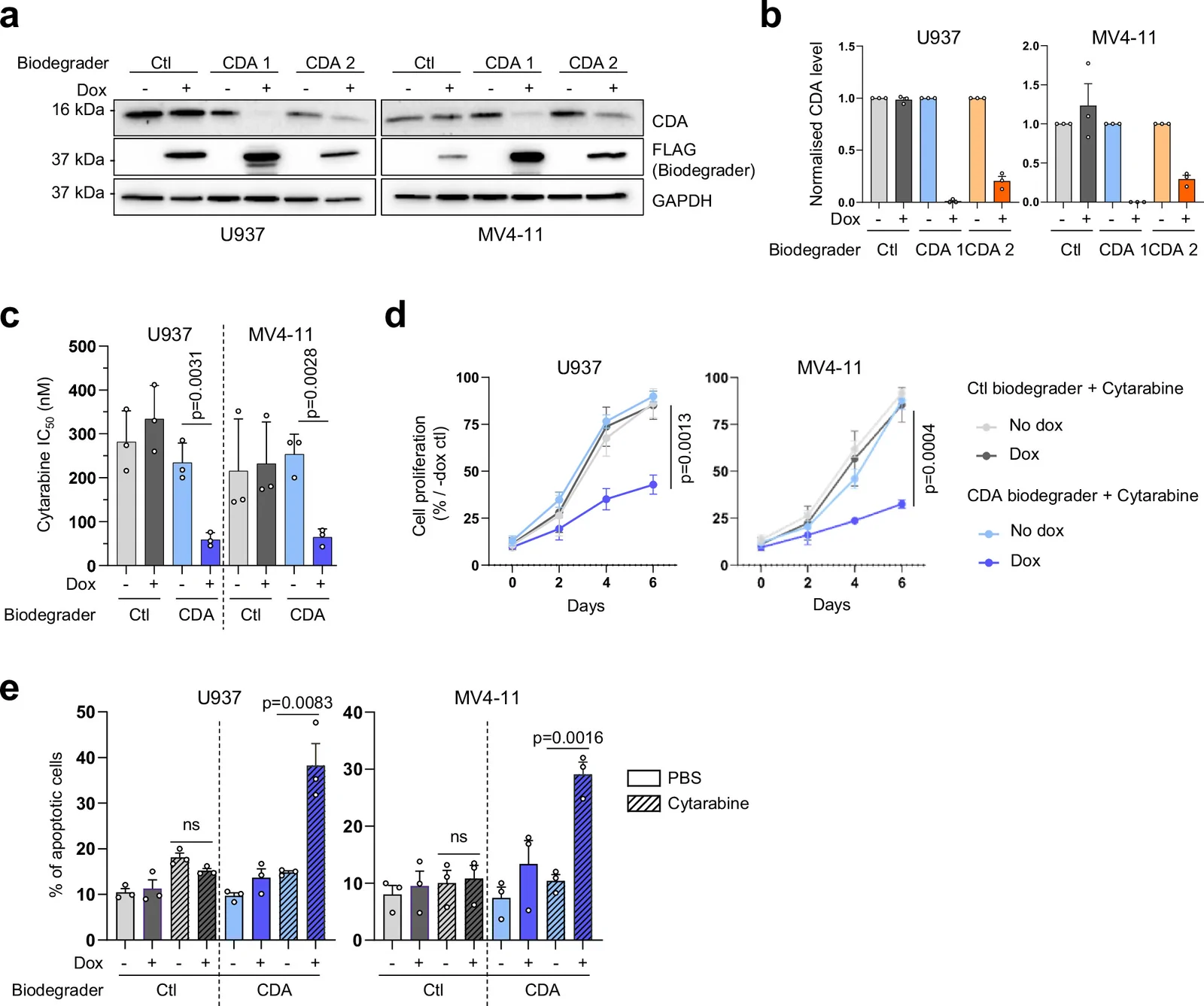
CDA biodegrader sensitises AML cells to cytarabine in vitro. **a** Efficacy of CDA biodegraders 1 and 2 to deplete CDA in U937 and MV4-11 cell lines after 24 h of doxycycline induction compared to the Ctl biodegrader was determined by Western blotting. GAPDH is the loading control. **b** Quantification of CDA protein level from (**a**) normalised to the no dox (-) condition of each biodegrader. **c** U937 and MV4-11 cell lines expressing either the Ctl or CDA biodegrader ( + dox) or not (- dox) were treated for 6 days with a range of cytarabine concentrations. The IC_50_ values of cytarabine were determined at day 6 with the Cell Titre Glo assay for both cell lines. **d** Cell proliferation of U937 and MV4-11 cell lines expressing either the Ctl or CDA biodegrader (dox) or not (no dox) and treated with cytarabine at 60 nM (the IC_50_ value), for 6 days. **e** Percentage of apoptotic cells determined by Annexin V/Propidium Iodide staining of U937 and MV4−11 cell lines expressing either the Ctl or CDA biodegrader ( + dox) or not (- dox) treated (crossed bars) or not with cytarabine at the IC_50_ value for 6 days. The data are displayed as percentage of the Annexin V positive/Propidium Iodide negative cell population from the total cell population after flow cytometry analysis. Statistical significance was determined by a two-tailed unpaired Student’s t-test in (**c**–**e**). ns not significant. Error bars indicate mean ± SD (**c**) or SEM (**b**, **d**, **e**) of biological repeats. Each experiment in (**a**–**e**) was performed three times as biological replicates. Source data are provided as a Source Data file.

CDA has also been implicated in the resistance to cytarabine^7^, a chemotherapy used for AML treatment^6^. To broaden the potential utility of CDA biodegraders to another dC analogue and cancer type, we investigated whether CDA degradation could sensitise AML cells to cytarabine in vitro. We first assessed CDA protein levels in three AML cell lines: HL-60, U937 and MV4-11. While CDA was undetectable in HL-60 cells, it was expressed in both U937 and MV4-11 cells (Supplementary Fig. 6a). The three cell lines were engineered to express either the Ctl or CDA biodegraders under the control of doxycycline. Expression of CDA biodegrader 1 induced the complete depletion of CDA protein in U937 and MV4-11 cell lines, while CDA biodegrader 2 showed lower efficacy (Fig. 5a, b), consistent with observations in pancreatic and lung cancer cell lines. Of note, a band with minor intensity was observed underneath the CDA biodegrader 1 expected band (Fig. 5a). This might result from a byproduct of the biodegrader, which is only seen in the AML cell lines. We next evaluated the functional impact of CDA depletion in these AML cell lines with CDA biodegrader 1 (named CDA biodegrader). Expression of CDA biodegrader significantly reduced cytarabine IC_50_ values in CDA-positive cell lines compared to cytarabine treatment alone, while the Ctl biodegrader had no effect (Fig. 5c). Importantly, the cytarabine IC_50_ in HL-60 cells, which have undetectable CDA level, remained unchanged upon expression of CDA biodegrader (Supplementary Fig. 6b), suggesting an on-target mechanism. Then, co-treatment with CDA biodegrader and cytarabine significantly impaired proliferation of U937 and MV4-11 cells (Fig. 5d). Neither the Ctl nor CDA biodegrader alone affected the proliferation of these cells (Supplementary Fig. 6c). In addition, HL-60 cell viability remained unchanged after 6 days of expression of the Ctl or CDA biodegrader (Supplementary Fig. 6d). We finally explored the molecular mechanisms underlying the proliferation inhibition measured in Fig. 5d by flow cytometry and Western blot analyses. Apoptosis was significantly increased when the cells expressed CDA biodegrader combined to cytarabine treatment compared to cytarabine alone (Fig. 5e and Supplementary Fig. 6e). These results highlight that the chemosensitisation efficiency of CDA biodegrader is not limited to pancreatic and lung cancer cells treated with gemcitabine, as it also sensitises AML cells to cytarabine, another dC analogue, in vitro.

### CDA biodegrader chemosensitises pancreatic and lung tumours to gemcitabine in vivo

We then evaluated the efficacy of CDA biodegrader in subcutaneous cell-derived xenograft (CDX) mouse models. From the previously established BxPC-3 and H441 stable cell lines, we isolated individual clones expressing either the Ctl (BxPC-3) or CDA biodegrader (BxPC-3 and H441) to ensure homogenous biodegrader expression across all cells. These clones were first characterised in vitro by Western blot and IC_50_ determination. Induction of CDA biodegrader expression in both BxPC-3 and H441 cells led to CDA protein depletion (Supplementary Fig. 7a) and significantly decreased gemcitabine IC_50_ values (Supplementary Fig. 7b, c), whereas the Ctl biodegrader had no effect on CDA level (Supplementary Fig. 7a).

**Fig. 6:**
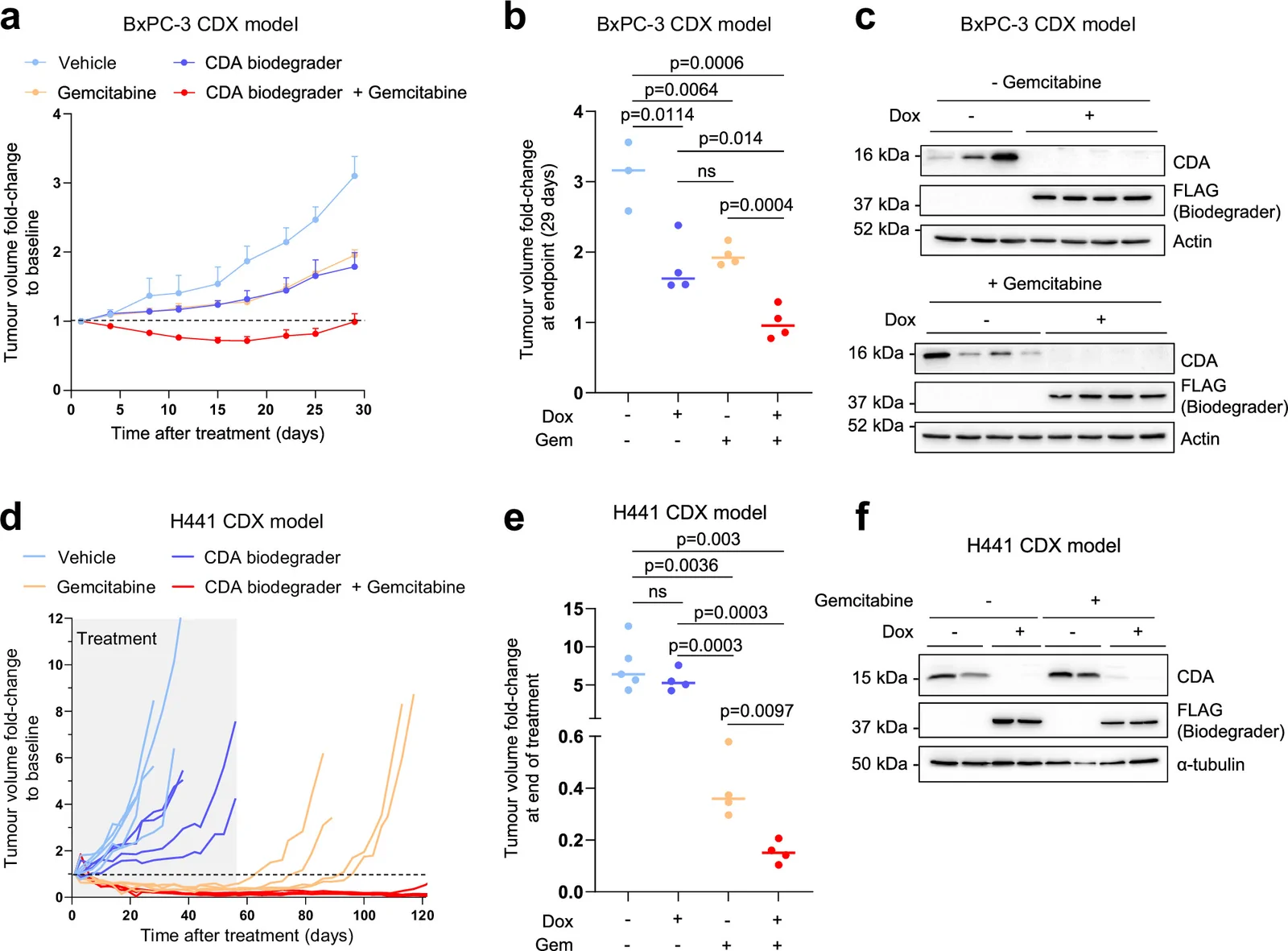
CDA biodegrader potently inhibits tumour growth in combination with gemcitabine in vivo. **a** Tumours volume fold-change from BxPC-3 cell-derived xenograft (CDX) model. Tumours were treated with vehicle (*n* = 3 mice), gemcitabine (40 mg.kg^−1^, *n* = 4 mice), doxycycline (2 mg.mL^−1^ to induce expression of CDA biodegrader, *n* = 4 mice) or doxycycline and gemcitabine combination (*n* = 4 mice). Tumours were normalised to the first day of treatment. **b** Scatter dot plot displaying the tumour volume fold-change at endpoint from (**a**). Bars show the median value. **c** Western blot analysis of individual BxPC-3 CDX tumours from (**a**) resected at endpoint (*n* = 4, excepted vehicle *n* = 3). The expression of CDA biodegrader is shown with the FLAG antibody. Actin is the loading control. **d** Individual tumours volume fold-change monitored from H441 cell-derived xenograft (CDX) model. Tumours were treated with vehicle (*n* = 5 mice), gemcitabine (25 mg.kg^−1^, *n *= 4 mice), doxycycline (2 mg.mL^−1^, *n* = 4 mice) or doxycycline and gemcitabine combination (*n* = 4 mice). Individual tumours were normalised to the first day of treatment. After 58 days, the treatments (gemcitabine monotherapy and CDA biodegrader/gemcitabine treatment combination) were stopped and the corresponding tumour volume was measured as indicated until 121 days. **e** Scatter dot plot showing the tumour volume fold-change from (**d**) at endpoint of the treatment period. Bars show the median value. **f** Western blot analysis of individual H441 CDX tumours resected after 14 days of treatment (*n* = 2 for each treatment). The expression of CDA biodegrader is shown with the FLAG antibody. α-tubulin is the loading control. Statistical significance was determined by a two-tailed unpaired Student’s t-test in (**b**, **e**). ns not significant. Error bars indicate mean ± SEM (**a**) of biological repeats. Source data are provided as a Source Data file.

Doxycycline-inducible cells were injected subcutaneously in immunodeficient mice to establish CDX models and to evaluate the impact of the CDA biodegrader, the gemcitabine, or their combination on tumour growth. In the BxPC-3 pancreatic cancer CDX model, both CDA biodegrader and gemcitabine monotherapies decreased tumour volume after 29 days of treatment (Fig. 6a, b). Importantly, the combination of CDA biodegrader and gemcitabine reduced tumour volume significantly more than either monotherapy at endpoint (Fig. 6a, b). None of the treatments affected mouse body weight, indicating good tolerability (Supplementary Fig. 7d). Tumours were resected at endpoint and analysed by Western blot. CDA biodegrader was expressed and CDA depleted in each of the corresponding tumours (Fig. 6c). Given the inhibitory effect of the CDA biodegrader alone on the tumour volume (Fig. 6a, b), we evaluated whether this could be due to the doxycycline and/or expression of the SPOP E3 ligase itself. To this end, we established a CDX model with BxPC-3 expressing the Ctl biodegrader. Doxycycline-induced expression of the Ctl biodegrader had no effect on the tumour volume (Supplementary Fig. 7e, f) or mouse body weight (Supplementary Fig. 7g). Western blot analysis confirmed Ctl biodegrader expression in tumours at endpoint (Supplementary Fig. 7h).

In the H441 CDX lung cancer model, mice were treated for 58 days with doxycycline, gemcitabine, or the combination. While CDA biodegrader alone had no significant effect on tumour volume, gemcitabine monotherapy induced approximatively 50% tumour regression (Fig. 6d, e). Remarkably, the combination CDA biodegrader and gemcitabine induced a nearly complete tumour regression after 58 days of treatment (Fig. 6d, e). To assess whether the tumours were completely eradicated under gemcitabine monotherapy or combination treatment, both treatments were discontinued and tumour volume was monitored for an additional two months. Following treatment withdrawal, all tumours previously treated with gemcitabine alone relapsed. In contrast, tumours treated with the combination remained in complete regression, with the exception of a single tumour that began to regrow (Fig. 6d). Mice body weight remained stable throughout the 58-day treatment period; however, it decreased in mice whose tumours relapsed following gemcitabine monotherapy (Supplementary Fig. 7i). To analyse CDA biodegrader expression and CDA depletion in tumours, two mice were treated with doxycycline and/or gemcitabine for 14 days. Western blot analysis confirmed both CDA biodegrader expression and effective CDA degradation (Fig. 6f).

These doxycycline-inducible CDX models demonstrate a proof-of-concept that CDA biodegrader efficiently sensitises tumours to gemcitabine, inducing potent inhibition in the pancreatic cancer CDX model and near-complete regression in the lung cancer CDX model.

### CDA biodegrader mRNA delivery via virus-like particles chemosensitises cancer cells to gemcitabine in vitro and in vivo

**Fig. 7:**
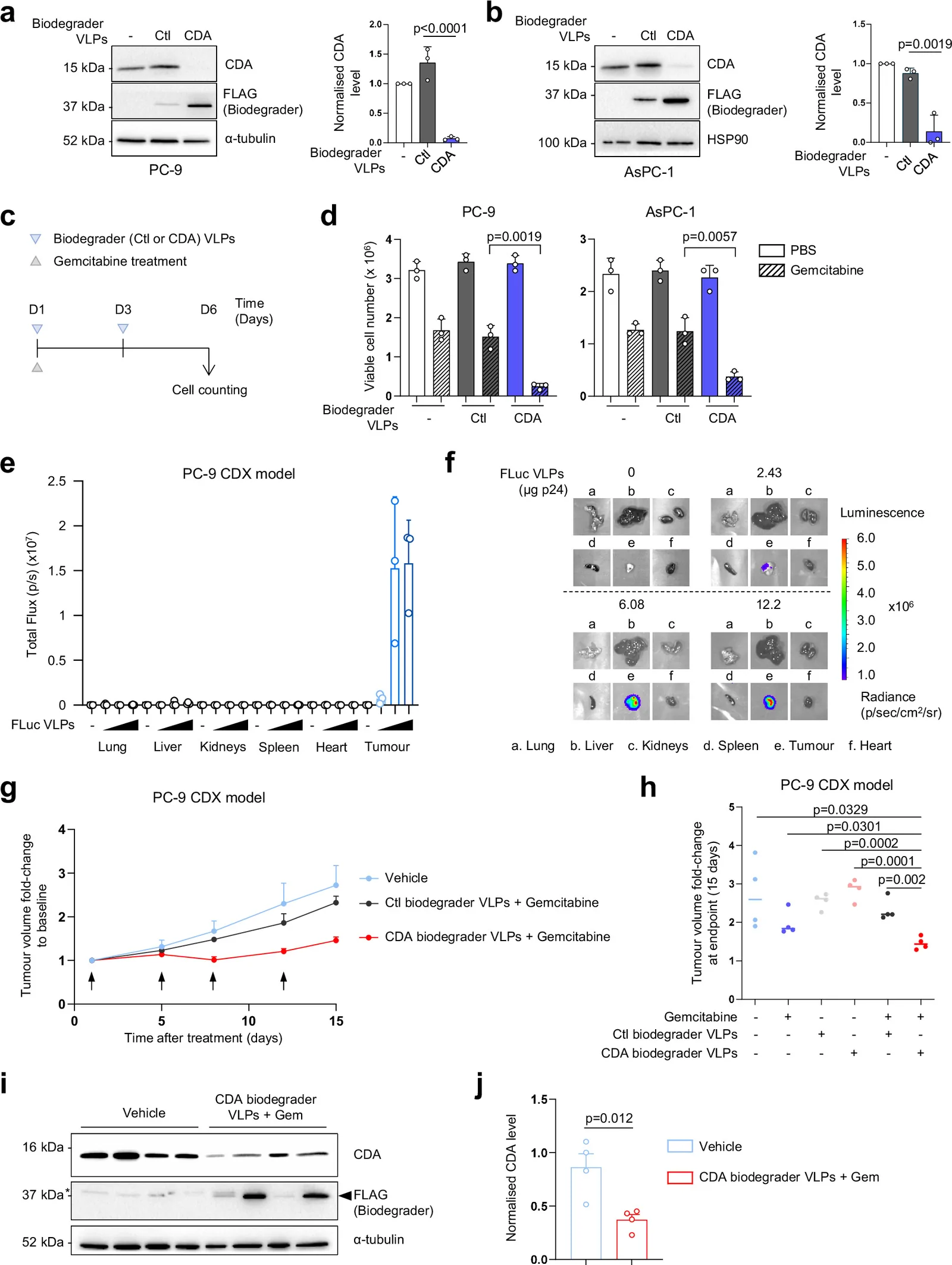
CDA biodegrader mRNA delivery via VLPs sensitises cancer cells to gemcitabine in vitro and in vivo. Western blot analysis of CDA and biodegraders protein levels 24 h after transduction or not (-) with 10 µg p24/cell of Ctl or CDA biodegrader VLPs in PC-9 (**a**) and AsPC-1 cells (**b**). α-tubulin and HPS90 are the loading controls. Quantification of CDA protein level normalised to the loading control is shown on the right-hand side of (**a**, **b**). **c** Schematic representation of the chemosensitisation experiment. **d** Number of viable PC-9 and AsPC-1 cells after 6 days of treatment with or without gemcitabine, and with or without transduction with Ctl or CDA biodegrader VLPs. **e**, **f** FLuc expression was assessed ex vivo by bioluminescence imaging in tumours and indicated organs, 6 h after intratumoral injection of increasing doses of FLuc mRNA-loaded VLPs (*n* = 3 mice per group). Total flux quantification is shown in (**e**) and representative images of each organ and tumour at the different doses are displayed in (**f**). **g** Tumours volume fold-change from PC-9 CDX model. Tumours (*n* = 4 mice per group) were treated with vehicle, Ctl biodegrader VLPs and gemcitabine (40 mg.kg^−1^) or CDA biodegrader VLPs and gemcitabine combination. Arrows indicate the injection times of the treatments (VLPs and/or gemcitabine). Tumours were normalised to the first day of treatment. **h** Scatter dot plot displaying the tumour volume fold-change at endpoint from (**g**) and Supplementary Fig. 9c (*n* = 4). Bars show the median value. (**i**) Western blot analysis of individual PC-9 CDX tumours from (**g**) resected at endpoint (*n* = 4). α-tubulin is the loading control. The star (*) indicates the top band obtained with the FLAG antibody, which is a non-specific band. **j** Quantification of CDA protein level normalised to the loading control from (**i**) (*n* = 4). Statistical significance was determined by a two-tailed unpaired Student’s *t* test in (**a**, **b**, **d**, **h**, **j**). Error bars indicate mean ± SD (**a**, **b**, **d**, **e**) or SEM (**g**, **j**) of biological repeats. Each experiment in (**a**–**d**) was performed three times as biological replicates. Source data are provided as a Source Data file.

CDA biodegrader is an intracellular protein, and its delivery into the cytoplasm is required for potential translational applications. As proof-of-concept, we investigated an mRNA delivery method based on the FlashRNA^®^ vector, which enables transient mRNA delivery via non-integrative VLPs^40^. This approach was recently employed to express intracellular therapeutic proteins in vivo^41^. First, we assessed which cancer cell lines could be efficiently transduced by VLPs loaded with ZsGreen mRNA (hereafter named ZsGreen VLPs). Three pancreatic and three lung cancer cell lines were transduced with ZsGreen VLPs in a dose-response experiment. Real-time microscopy revealed rapid expression of ZsGreen as early as 6 to 12 h post-transduction in PC-9 and AsPC-1 cells, whereas the green signal was lower in the other cell lines (Supplementary Fig. 8a, b). Flow cytometry analysis indicated that all cell lines were transduced by VLPs in a dose dependent manner, though with varying efficiencies (Supplementary Fig. 8c–e). While only 30% of H441 and Capan-2 cells expressed ZsGreen at the highest dose of VLPs, this percentage was ≥75% for the four other cell lines, even approaching 100% for PC-9 and AsPC-1 cells (Supplementary Fig. 8c–e). Moreover, the percentage of ZsGreen-positive cells correlated positively with the fluorescence intensity in those cells (Supplementary Fig. 8d, e). Based on these results, we chose to continue the delivery study using PC-9 and AsPC−1 cells, which exhibited the highest VLP transduction and transgene expression levels. Next, we evaluated whether Ctl and CDA biodegraders could be delivered via VLPs in PC-9 and AsPC-1 cells using Western blot analysis. Both biodegraders were expressed 24 h after transduction with VLPs loaded with their respective mRNAs (Fig. 7a, b). Importantly, expression of CDA biodegrader led to degradation of the CDA protein, whereas the Ctl biodegrader had no effect on CDA protein level (Fig. 7a, b). We then assessed the kinetics of CDA biodegrader and CDA protein expression over 6 days following a single transduction with CDA biodegrader VLPs. The CDA biodegrader was expressed, and CDA protein was depleted for 2 days in PC-9 cells and 3 days for AsPC-1 cells (Supplementary Fig. 8f, g). After these time points, CDA levels gradually returned to baseline (Supplementary Fig. 8f, g), suggesting that an additional transduction would be required for experiments extending beyond 2–3 days. Finally, we investigated whether CDA biodegrader delivery could sensitise PC-9 and AsPC-1 cells to gemcitabine in vitro. Based on the kinetic experiments, each cell line was transduced on day 1 and day 3, treated or not with gemcitabine on day 1, and the cell number was determined on day 6 (Fig. 7c). As expected, gemcitabine treatment of untransduced cells or cells transduced with Ctl biodegrader VLPs reduced cell number by 50% compared to their respective untreated controls (Fig. 7d). The number of CDA biodegrader VLPs transduced cells treated with gemcitabine decreased by 85 and 90% compared to the untreated control in AsPC-1 and PC-9 cells, respectively (Fig. 7d). This represented a significant reduction compared to cells treated with gemcitabine and transduced with Ctl biodegrader VLPs (Fig. 7d). This drop in cell number was accompanied by a significant increase in apoptosis in PC-9 cells, but not in AsPC-1 (Supplementary Fig. 8h, i). These data demonstrate that VLP-mediated delivery of the CDA biodegrader sensitises cancer cells to gemcitabine in vitro.

To evaluate the in vivo therapeutic efficacy of this strategy, we first explored whether VLPs could deliver mRNA in vivo using the PC-9 CDX mouse model (immunodeficient mice). To this end, Firefly Luciferase (FLuc) mRNA-loaded VLPs were intratumorally injected at different doses (0; 2.43; 6.08 and 12.2 µg p24) and the bioluminescent imaging (BLI) was measured ex vivo 6 h after injection with the IVIS spectrum instrument (Fig. 7e, f). A bioluminescent signal was detected exclusively in tumours and followed a dose-response relationship, whereas no signal was observed in any of the analysed organs (i.e. lung, liver, kidneys, spleen, heart, Fig. 7e, f). Then, we injected the highest dose of VLPs (i.e. 12.2 µg p24) and measured the bioluminescent signal 6, 24 and 48 h after. The signal was maximal at 6 h and decreased over time to almost disappear after 48 h (Supplementary Fig. 9a, b). These data show that VLPs intratumoral injection (IT) leads to an efficient, transient and localised mRNA delivery in tumours.

Finally, we assessed the efficacy of CDA biodedegrader VLPs in PC-9 CDX mouse model. The combination gemcitabine/CDA biodegrader VLPs significantly inhibited tumour growth compared to all the other groups (Fig. 7g, h and Supplementary Fig. 9c). Importantly, VLPs treatment did not affect body weight compared to vehicle or the gemcitabine treatments, suggesting a good biosafety in tumour-bearing mice (Supplementary Fig. 9d). We confirmed the expression of the FLAG-tagged biodegraders by Western blot (Fig. 7i and Supplementary Fig. 9e). In addition, CDA biodegrader induced a ~ 40–60% degradation of CDA in tumour tissues compared to the other groups (Fig. 7j, Supplementary Fig. 9e). Altogether, these findings demonstrate the in vivo efficacy of VLP-based delivery of CDA biodegrader in promoting CDA degradation and sensitising tumours to gemcitabine.

## Discussion

Intracellular biodegraders have been used to target a large variety of proteins including hard-to-drug proteins such as BCL11A^23,42^, KRAS^22,43,44^, YAP^32^, misfolded SOD1 protein^45^ or HuR^24^. Designing effective biodegraders that deplete their targets in cells is challenging. First, antibody fragments such as VHHs may lose functionality in the reducing intracellular environment^46^; second, not all E3 ligases are capable of degrading a given target^34,47^; and finally not all E3 ligase-VHH combinations yield functional biodegraders^33^. To overcome these challenges, we reported here a two-step cell-based screening. We first adapted our E3 ligase-based screening^34,35^ to identify which E3 ligase, when fused to an anti-GFP VHH, could deplete the GFP-CDA fusion in cells. We showed that only SPOP-based GFP biodegrader led to GFP-CDA degradation. Using SPOP as E3 ligase moiety in a biodegrader vector, we then modified our previously reported microscopy cell-based screening of intracellular biodegraders^19,48^ into a flow cytometry-based screening. This second screen serves as a powerful dual-purpose platform, enabling rapid identification of intracellular anti-CDA VHHs post-phage display and selection of VHH-SPOP combinations with degradation activity. Compared to our previous method, flow cytometry is quantitative and offers higher throughput. Importantly, the three most potent hits depleted endogenous CDA in cells, highlighting the utility of this two-steps screening strategy, which could be applied to virtually any target in the future.

The catalytic site of CDA is targeted by small molecule inhibitors such as tetrahydrouridine (THU) and zebularine, but these inhibitors were shown to display off-target effects^12,13^ and/or a lack of efficacy in patients^11^. Biodegraders represent a promising alternative by directly addressing this major limitation through their high specificity towards their target^24,30^. We demonstrated here the specificity of CDA biodegrader with a global proteomic analysis by mass spectrometry, in which CDA was the only protein significantly decreased in cells expressing CDA biodegrader. In addition, CDA biodegrader had no effect on cells that did not express CDA: neither sensitising these cells to dC analogues nor affecting their cell viability at the basal state, confirming the specificity of action of CDA biodegrader. We found that cancer cell lines, including pancreatic, lung, and AML, exhibit different levels of CDA expression (Supplementary Figs. 4a, b, and 6a), which did not directly correlate with their IC_50_ values for dC analogues. These data are consistent with previous reports, including the GemPred signature^49^, which show that CDA expression alone cannot predict gemcitabine sensitivity, highlighting the involvement of additional factors such as the hENT1 transporter^50^. Nevertheless, our study indicates that CDA-targeted degradation is sufficient to sensitise cancer cell lines to dC analogues, regardless of their baseline CDA expression levels. Interestingly, the CDA biodegrader alone suppressed BxPC-3 CDX tumour growth (Fig. 6a, b), but had no effect on H441 CDX tumours (Fig. 6d, e), despite no measurable differences in 2D proliferation in either cell line (Fig. 4c, d). These findings suggest that the tumour growth inhibitory effects of CDA degradation may be context dependent, potentially reflecting cell line-specific differences in gene regulatory programmes that emerge under 3D growth conditions^51^. Similar context-specific dependencies have been described for KRAS signalling, where phenotypic effects are often more pronounced in 3D than in 2D assay systems and vary substantially across cell lines^52,53^.

We demonstrated the versatility of the CDA biodegrader approach by efficiently sensitising three distinct cancer models to two dC analogues, gemcitabine and cytarabine. Interestingly, dC analogues are also used in other malignancies, such as gemcitabine in ovarian^54^ and bladder^55^ cancers, and decitabine in myelodysplastic syndromes^56^. Depending on CDA expression levels, the CDA biodegrader could also be effective in these settings, thereby broadening its utility for dC analogues sensitisation. Moreover, we recently showed that CDA is implicated in resistance to DNA-targeting drugs in pancreatic cancer^38^. Other groups demonstrated CDA involvement in the resistance to ceritinib, an ALK tyrosine kinase inhibitor, in lung cancer^39^, and to immunotherapies^57^. Therefore, the applicability of the CDA biodegrader may extend beyond dC analogues, offering broader potential for overcoming drug resistance in cancer.

The clinical application of biodegraders remains limited because these molecules cannot penetrate cell membranes. The development of effective delivery systems is a key hurdle to translating this strategy into clinically meaningful outcomes. To date, only a few methods have been evaluated, including direct administration of proteins fused to CPP^23^, encapsulated mRNA^30^ or proteins^28^ in LNPs, or AAV-mediated delivery of biodegrader^25,32^. To address this challenge, and based on a previous study from our group^58^, we explored, as proof-of-concept, a VLP strategy loaded with mRNA encoding for the CDA biodegrader using the FlashRNA^®^ vector. VLPs mimic the form and size of a virus but they lack the genetic material and are non-integrative vectors^59^. They exhibit cellular uptake comparable to that of natural virus, maintain cell viability, and escape endosomal compartments before lysosomal degradation^40,59^. We demonstrated that VLP-mediated delivery of the CDA biodegrader efficiently sensitises cancer cells to gemcitabine in vitro and in vivo. mRNA is a key component of the delivery method, as it should enable prolonged transgene expression to maximise the effect on target cells. Here, we observed that the half-lives of the CDA biodegrader and, consequently, of the CDA protein were dependent on the cell lines (Supplementary Fig. 8f, g). The faster dividing PC-9 cells (doubling time ~18–26 h) exhibited a shorter duration of CDA biodegrader expression (2 days) compared to AsPC-1 cells (3 days), which have a longer doubling time (~38–40 h). This difference affected CDA protein re-expression, which occurred more rapidly in PC-9 cells (3 days) than in AsPC−1 cells (4 days). The latter parameter likely also depends on the baseline CDA protein expression level in each cell line. Nevertheless, after a single transduction, CDA biodegrader activity persisted for more than 2 days, which is longer than the recently reported activity of a GFP biodegrader delivered via mRNA or protein-loaded LNPs ( < 2 days)^28^. While AsPC-1 cells were sensitised to gemcitabine, the increase in apoptosis was not statistically significant compared to PC-9 cells (Supplementary Fig. 8h, i). In contrast, apoptosis was significantly increased in the doxycycline-inducible AsPC-1 cell line model (Fig. 4e). These findings suggest that CDA biodegrader delivered via VLPs induces a cytostatic effect, whereas the doxycycline-inducible model triggers a more cytotoxic response. This discrepancy may result from differences in the amount of CDA biodegrader expression in the cells, leading to varying CDA degradation efficiencies between the two methods. In vivo delivery of CDA biodegrader VLPs in PC-9 CDX model resulted in effective CDA depletion in tumour and was associated with tumour growth inhibition upon co-treatment with gemcitabine, demonstrating the delivery efficacy of the FlashRNA^®^ vector. To fully evaluate the in vivo efficacy of the CDA biodegrader, it will be important to assess its delivery in additional tumour models that present greater biological challenges than the PC-9 CDX model, including higher growth rates and greater resistance to gemcitabine.

Although the in vivo data obtained in PC-9 tumours are promising, it is noteworthy that CDA degradation within tumours was incomplete, in contrast to the near-complete degradation observed in vitro. These findings suggest that robust expression of the biodegrader across the majority of tumour cells may be required to achieve near-complete target depletion, thereby maximising in vivo efficacy. Accordingly, further optimisation of both the CDA biodegrader and its delivery strategy will be necessary to improve biodegrader stability and expression at both the mRNA and protein levels. At the mRNA level, strategies may include codon optimisation of the coding sequence and modification of the 5′ and/or 3′ untranslated regions (UTRs) to improve translational efficiency^60,61^. mRNA stability may be further increased through nucleoside modifications (e.g. substitution of uridine with pseudouridine) and cap structure engineering (e.g. Cap 1 or Cap 2 structures)^60,61^. Finally, other RNA platforms providing enhanced and prolonged protein expression, such as self-amplifying RNA^62^ or circular RNA^63^, could be explored. Collectively, these approaches are expected to enhance and sustain biodegrader expression.

Although we demonstrate that the CDA biodegrader remains expressed in cells for at least 2 days following delivery, its intracellular protein stability may be further improved through lysine residue mutagenesis^44^ and/or the introduction of stabilising mutations within the VHH framework^46^. These approaches have previously been shown to reduce intracellular proteolysis of a KRAS biodegrader^44^ and to enhance the stability of intracellular VHHs^46^, respectively. In parallel, even though we demonstrate a near-complete CDA degradation in vitro, degradation potency may be further improved through several complementary strategies. For example, a multivalent CDA biodegrader could be developed, as this format has previously been shown to maximise the degradation efficacy of SHP2 biodegraders^64^. Increased potency may also be achieved through the identification of alternative E3 ligases with greater activity than SPOP. Finally, the delivery strategy itself could be further refined, either by increasing the concentration of VLPs administered intratumourally or by implementing alternative delivery platforms such as nanoparticles.

Once both the CDA biodegrader and the delivery method have been optimised, additional work will be required before advancing this therapeutic strategy towards clinical application. In this proof-of-concept study, VLPs were administered intratumourally in mice, as achieving systemic tumour-specific delivery remains a major challenge. Although IT injection has an important clinical limitation, i.e. not applicable in patients with metastatic disease, it also offers several advantages, including a high therapeutic index and low systemic toxicity^65^. Notably, IT injection has previously been used in a clinical gene therapy trial (phase I/II, NCT02806687)^66^, supporting the clinical feasibility of this route of administration in pancreatic cancer.

In the present study, VLPs were administered twice weekly in mice, a dosing frequency that is unlikely to be clinically practical. By contrast, an administration schedule of once every two weeks would be more feasible^67^. Achieving such a schedule will depend directly on the optimisation strategies outlined above to extend the half-life of both the delivered mRNA and the biodegrader protein. Importantly, repeated dosing also raises potential immunogenicity concerns related to both the delivery vehicle and its cargo. Although IT delivery may partially mitigate this risk, immunogenicity will require careful evaluation before clinical translation.

From a translational perspective, we do not anticipate that clinical implementation of the CDA biodegrader would alter the dosing regimens of dC analogues or directly expand their therapeutic index. Rather, the principal benefit is expected to derive from enhanced tumour sensitivity to these agents.

Overall, our study provides compelling proof-of-concept for the development of CDA biodegraders as a strategy to sensitise cancer cells to dC analogues. Further research will be required to improve the translatability of this approach towards clinical applications.

## Methods

### Cell culture

HeLa-S3 (ATCC, CCL-2.2), HEK293T (ATCC, CR-1573) and MIA PaCa2 (ATCC, CRL-1420) were grown in Dulbecco’s Modified Eagle Medium (DMEM). AsPC-1 (ATCC, CRL-1682), BxPC-3 (ATCC, CRL-1687), Capan-2 (ATCC, HTB-80), PC-9, HCC4006, H441 (ATCC, HTB−174) and BEAS-2B were grown in Roswell Park Memorial Institute (RPMI) medium. PC-9, HCC4006 and BEAS-2B cell lines were a gift from Dr. Olivier Calvayrac and Rémi Gence (CRCT, Toulouse, France). MRC-5 (ATCC, CCL-171) was grown in Minimum Essential Medium (MEM)-α supplemented with MEM Non-Essential Amino Acids Solution (Thermo Fisher Scientific, Cat#11140-035). All cell lines were supplemented with 10% foetal bovine serum (FBS, Thermo Fisher Scientific) and 1% penicillin/streptomycin (Life Technologies) and grown at 37 °C in a humidified incubator with 5% CO_2_. They were tested and confirmed to be mycoplasma free.

### Cell transfection and treatments

Transient transfection of DNA plasmids were performed using JetPRIME reagent, as indicated by the supplier (PolyPlus Transfection, Cat# POL101000046). Proteasome inhibition was done by adding 0.8 µM of proteasome inhibitor epoxomicin (Sigma, Cat#E3652-50UG) in cell growth medium for 18 h. Doxycycline (Sigma, Cat#D9891-25G) was used to induce the expression of the biodegraders in stable cell lines at 0.5 µg.mL^−1^ (unless indicated otherwise). Gemcitabine (Sigma, Cat#G6423) and cytarabine that was kindly provided by the pharmacy of the Toulouse University Hospital were used to treat cells at the indicated concentrations.

### Molecular cloning

#### Phage display plasmids construction

CDA was amplified by PCR and inserted into pCMV-2xStrep-MCS and pCMV-CBD-MCS^33^ with KpnI/BamHI sites to generate pCMV-2xStrep-CDA and pCMV-CBD-CDA plasmids.

#### Biodegrader plasmids construction

pEF-FLAG-VHH GFP4-L5-SPOP-, pEF-FLAG-VHH ALFA-L5-SPOP- (VHH ALFA = VHH Ctl), pEF-VHL-L5-VHH GFP4-FLAG-, pEF-VHL-L5-VHH Ctl-FLAG-, pEF-FLAG-VHH GFP4-L5-UBOX-, pEF-FLAG-VHH Ctl-L5-UBOX-IRES-MTS-mCherry plasmids construction were previously reported^34^.

VHH Ctl, and all anti-CDA VHHs were amplified by PCR and inserted into pEF-3xFLAG-VHH plasmid^34^ with NotI/XbaI sites. IRES-MTS-mCherry^33^ was inserted into the newly generated pEF-3xFLAG-VHH plasmids digested by XbaI.

CDA was amplified by PCR and inserted into the pEF-cyto-myc plasmid (Invitrogen, Cat#V89120) with NcoI/NotI sites.

#### BRET plasmids construction

AID cDNA was amplified by PCR from genomic DNA and inserted into pEF-MCS-(GGGGS)_2_-RLuc8^68^ with NcoI/XhoI sites. pEF-CDA-(GGGGS)_2_-RLuc8 was described elsewhere^69^. CDA K27Q was generated by PCR site-directed mutagenesis and inserted into pEF-MCS-(GGGGS)_2_-RLuc8 with NcoI/XhoI sites. VHH Ctl, VHH CDA1 and VHH CDA 2 was inserted between NotI/XbaI into pEF-GFP^2^-(GGGGS)_2_-MCS^68^.

#### Lentivectors construction

FLAG-Ctl biodegrader-SPOP, FLAG-CDA biodegrader 1-SPOP and FLAG-CDA biodegrader 2-SPOP sequences were inserted between AgeI/NheI sites of TLCV2 lentivector (Addgene plasmid #87360)^70^ by PCR.

GFP^2^-CDA was amplified by PCR and inserted into pLenti-CMV-blast-DEST (Addgene plasmid #17451)^71^ by Gateway cloning (Thermo Fisher Scientific).

#### E.coli proteins expression construction

CDA was amplified by PCR and inserted into pET-2xStrep-HA-MCS plasmid^72^ with KpnI/BamHI sites. VHH Ctl, CDA 1 and 2 were inserted into pAOT7-VHH-6xHIS-myc-6xHIS plasmid^73^ with NcoI/NotI sites.

#### Lentivirus production

4.5 × 10^6^ HEK293T cells were seeded in 100 mm dish in 9 mL of complete DMEM. 24 h later, cells were transfected with 6 μg of the TLCV2 construct of interest (i.e. Ctl biodegrader, CDA biodegrader 1 and CDA biodegrader 2), 4 μg of psPAX2 (Addgene, Cat#12260), 1.5 μg of pMD2.G (Addgene, Cat#12259) - the two last are lentiviral packaging and envelope vectors, respectively - and 23 μL of JetPRIME (quantities for one 100 mm dish). The virions-containing media was collected 48 h after transfection, centrifuged 5 min at 850 × *g* and filtered (0.45 µm filter). The filtered media was concentrated in a Vivaspin concentrator (Sartorius, Cat#VS2042) and centrifuged at 1300 × *g* to concentrate it until the remaining volume was around 0.5 mL. A detailed protocol is provided elsewhere^35^.

### Cell transduction

#### With integrative lentiviral particles

Target cells were transduced with the appropriate lentiviral particles for 36 h in 6-well plate in 1 mL of medium containing 8 µg.mL^−1^ of polybrene (Sigma, Cat#107689). Transduced cells were selected with puromycin (Invitrogen, Cat#ant-pr-1) or blasticidin (Invitrogen, Cat#ant-bl-05) antibiotics.

#### With non-integrative FlashRNA^®^ virus-like particles (VLPs)

Cells were transduced at the indicated dose of FlashRNA^*®*^ vectors: 1, 5 and/or 10 pg p24/cell (RNAlead, Cat#LF7-ZsGreen11-002 or Cat# LF7.63) in media containing 4 µg.mL^−1^ of polybrene (Sigma, Cat#107689). 6 h later, the media was replaced with fresh media.

#### Phage display selection of anti-CDA VHHs

Phage display selection was performed using NaLi-H1 library of humanised synthetic single domain antibody^33^. 2xStrep-CDA or CBD-CDA were expressed transiently during 38 h in HEK293T cells and captured after cell lysis on magnetic beads either MagStrep Strep-Tactin XT beads (IBA, Cat#2-5090-002) or Chitin magnetic beads (NEB, Cat#E8036S), respectively. A phage display panning alternating rounds on streptactin beads (1 and 3) with rounds on chitin beads (2 and 4) was performed for 4 rounds. Antigen-coated beads were incubated with the phage library. Phage bound to antigen-coated beads were recovered on a magnet and beads were washed. Phages were eluted using trimethylamine (TEA, 100 mM, Sigma, Cat#90335-100 ML) for 7 min and neutralised using 1 M Tris/HCl pH 7.4. Eluted phages were used to re-infect *E.coli* TG1 cells and plated on ampicillin (100 µg.mL^−1^), 0.5% glucose containing 2XYT agar plates.

#### Cell-based screening of intracellular CDA biodegraders

VHHs obtained after the fourth round of phage display were directly subcloned by NcoI/NotI digestion into pEF-FLAG-VHH-SPOP-IRES-MTS-mCherry vector as previously described^48^. 5000 HeLa S3 stably expressing GFP^2^-CDA were seeded per well of 96-well plates and transfected 24 h later with 200 individual plasmids coding for the biodegraders. 48 h after transfection cells were analysed by flow cytometry. The median fluorescence intensity (MFI) of each biodegrader was analysed in the mCherry transfected cells and the results are given with a normalised GFP MFI (MFI of the degrader/MFI of the negative control x 100).

### Flow cytometry

#### GFP intensity in HeLa S3/GFP-CDA or HeLa S3/H2B-GFP cells

80,000 cells were plated in a 12-wells plate, transfected with 0.8 µg of plasmid DNA of interest. 48 h after transfection, cells were washed with 1xPBS, detached with trypsin and resuspended in 1xPBS-4% FBS and analysed on a MACSQuant VYB flow cytometer (Miltenyi) for GFP fluorescence intensity quantification. The instrument settings log forward scatter (FSC) and log side scatter (SSC) were adapted to the size of the cells. The singlet cell population was gated on FSC and SSC. The typical sample size was 20,000 singlets per measurement. The data were analysed with FlowJo software. The MFI was analysed in the mCherry transfected cells and the results are given with a normalised GFP MFI (MFI of the biodegrader/MFI of the negative biodegrader x 100).

#### ZsGreen intensity in VLPs transduced cells

15,000 cells were seeded in 96-wells plate in technical triplicate. On day later, VLPs were added as described in the cell transduction section. 48 h later, the percentage of ZsGreen-positive cells and the ZsGreen fluorescence intensity were measured using a MACSQuant VYB flow cytometer. Data analysis was performed with FlowJo software.

#### Cell apoptosis by Annexin V/PI staining

Cells were seeded in 60 mm dish at an optimal number to maintain linear growth over the time of the assay. For inducible cell lines, cells were treated 24 h later with or without doxycycline and with or without gemcitabine/cytarabine at the cell lines IC_50_ values. For VLPs delivery experiments, 300,000 of AsPC−1 or 100,000 of PC-9 cells were seeded in 60 mm dish. Ctl or CDA 1 biodegraders VLPs or not (-) were added at 10 pg p24/cell on day 1 and 3. Cells were also treated with or without gemcitabine on day 1 at the IC_50_ value of each cell line (80 nM for PC-9 and 100 nM for AsPC-1 cells).

6 days later, cell apoptosis was assessed with Annexin V/Propidium Iodide (PI) staining using the FITC Annexin V Apoptosis Detection Kit I (BD Biosciences, Cat #556547). Percentage of Annexin V positive and PI negative cells was determined on the MACSQuant VYB and analysed using FlowJo software.

#### BRET2 assays and measurements

650,000 HEK293T cells per well were seeded in a 6-well plate. The day after cells were transfected with a total of 1.6 μg of DNA mix (containing donor + acceptor plasmids), using using JetPrime (Polyplus) following manufacturer’s recommendations. Cells were detached after 24 h of transfection, washed with PBS and seeded in a white 96-well plate (clear bottom, Revvity, Cat#6005181) in OptiMEM no phenol red medium (Gibco™, Cat #1058021) complemented with 4% FBS. The BRET assay measurements were proceeded 20–24 h after. BRET2 signal was determined immediately after injection of coelenterazine 400a substrate (10 μM final) to cells (Cayman Chemicals, Cat #16157), using a CLARIOstar instrument (BMG Labtech) with a luminescence module. Total GFP^2^ fluorescence and total RLuc8 luminescence were also measured with the CLARIOstar instrument. Total GFP^2^ and RLuc8 signals were used to control the protein expression from each plasmid. A step-by-step protocol is provided elsewhere^68^.

#### Immunoprecipitation

650,000 HEK293T cells were seeded in 6 well-plates and co-transfected for 24 h with 1.8 µg of pEF-3xFLAG-VHH CDA 1/2 or pEF-3xFLAG-VHH Ctl and 0.2 µg pEF-CDA-myc plasmids. To immunoprecipitate endogenously expressed CDA, 3 × 10^6^ HeLa S3 cells were seeded in 100 mm dish and were transfected for 24 h with 10 µg of pEF-3xFLAG-VHH CDA 1/2 or pEF-3xFLAG-VHH Ctl.

After two washes with cold 1X PBS, cells were lysed with the immunoprecipitation buffer (150 mM NaCl, 50 mM Tris-HCl pH 7.4, 10 mM MgCl_2_, 10% glycerol and 0.5% Triton X-100) supplemented with protease inhibitors (Sigma, Cat#P8340) and phosphatase inhibitors (Thermo Fisher Scientific, Cat#1862495) for 20 min on ice. Cell lysis was then centrifuged for 15 min (12,000 × *g*, 4 °C). Immunoprecipitation was performed with anti-FLAG antibody (Sigma, Cat#F1804) and protein G magnetic beads (Life Technologies, Cat#10004D) for 2 h at 4 °C on a wheel. Beads were washed 3 times with the IP buffer and the bound proteins were eluted with 1x loading buffer and resolved on 12.5% sodium dodecyl sulfate polyacrylamide gel electrophoresis (SDS-PAGE).

### Western blot analysis

Cells were washed twice with PBS and lysed in RIPA buffer (50 mM Tris-HCl, 150 mM NaCl, 1% Nonidet 40, 1% NaDoc, 1% SDS) supplemented with protease inhibitors (Sigma, Cat#P8340) and phosphatase inhibitors (Thermo Fisher Scientific, Cat#1862495). Cell lysates were sonicated with a VibraCell, and the protein concentrations determined by Bradford assay (Fisher Scientific, Cat#11727936). Equal amounts of protein were resolved on 12.5% SDS-PAGE and subsequently transferred onto a nitrocellulose membrane (Bio-Rad). The membrane was blocked with 10% non-fat milk (Sigma, Cat#70166) or 10% BSA (Euromedex, Cat#04100812E) in TBS-0.1% Tween20 and incubated overnight with primary antibody at 4 °C. After washing, the membrane was incubated with HRP-conjugated secondary antibody for 1 h at room temperature (RT, 23 °C). The membrane was washed with TBS-0.1% Tween and developed using ClarityTM Western ECL Substrate (Bio-Rad) and Chemi-DocTM XR+ (Bio-Rad). Primary antibodies include anti-CDA (1/1000, ABClonal Technology, Cat#A13959), FLAG (1/2000, Sigma, Cat#F1804-200UG), α-tubulin (1/2000, Abcam, Cat#ab4074), pan-actin (1/50000, Millipore, Cat#MAB1501), CL-CASP3 (1/500, Cell Signalling Technology, Cat#9664S), CL-PARP (1/1000, Cell Signalling Technology, Cat#9541S), HSP90 (1/1000, Cell Signalling Technology, Cat#4874S), GAPDH (1/5000, Cell signalling Technology, Cat#5174S), PCNA (1/5000, Abcam, Cat #ab29). Secondary antibodies include anti-mouse-HRP (1/4000, Cell Signalling, Cat#7076S), Anti-rabbit-HRP, 1/4000, Cell Signalling, Cat#7074S).

### RNA analysis

Total RNA was extracted using the RNeasy Mini kit (Qiagen, Cat#74106) according to the manufacturer’s instructions. RNA concentration and purity were assessed using a NanoDrop spectrophotometer (Thermo Fisher Scientific). cDNA was obtained by reverse transcription of 1 μg of total RNA using RevertAid® Reverse Transcription Kit (Thermo Scientific, Cat#K1622) and analysed by quantitative PCR (qPCR) with StepOnePlus™ System (Life Technologies) and SsoFast™ EvaGreen® Supermix (Bio-Rad, Cat#1725204). Values are normalised to cyclophilin A mRNA, used as a control. These are the oligos used for the qPCR: CDA_For 5’ GGGGACAAGTTTGTACAAAAAAGCAGGCTATGGCTATGGCCCAGAAGCGT 3’, CDA_Rev 5’ GGGGACCACTTTGTACAAGAAAGCTGGGTCACTGAGTCTTCTG 3’, CycloA_For 5’ GTCAACCCCACCGTGTTCTT 3’ and CycloA_Rev 5’ CTGCTGTCTTTGGGACCTTGT 3’.

### Proteomic sample preparation and nano LC-MS/MS analysis

450,000 AsPC-1 cells conditionally expressing either CDA biodegrader or Ctl biodegrader were seeded in 6-well plates. 24 h later cells were treated or not with doxycycline for 10 h. At endpoint cells were lysed, sonicated and protein quantified as indicated in the Western blot analysis section. Three independent samples per condition (- and + doxycycline) and per cell line (CDA and Ctl biodegraders) were performed and analysed by nano LC-MS/MS.

Each sample dried protein extracts (40 µg) were solubilized with 25 µL of 5% SDS. Proteins were submitted to reduction and alkylation of cysteine residues by addition of TCEP and chloroacetamide to a final concentration respectively of 10 mM and 40 mM. Protein samples were then processed for trypsin digestion on S-trap Micro devices (Protifi) according to manufacturer’s protocol, with the following modifications: precipitation was performed using 302 µL S-Trap buffer; 2 µg Trypsin was added per sample for digestion, in 25 µL ammonium bicarbonate 50 mM pH7. Tryptic peptides were resuspended in 2% acetonitrile and 0.05% trifluoroacetic acid and analysed by nano-liquid chromatography (LC) coupled to tandem MS, using an UltiMate 3000 system (NCS-3500RS Nano/Cap System; Thermo Fisher Scientific) coupled to an Orbitrap QExactive Plus mass spectrometer (Thermo Fisher Scientific). Around 5 µg of each sample was loaded on a C18 precolumn (300 µm inner diameter ×5 mm, Thermo Fisher Scientific) in a solvent made of 2% acetonitrile and 0.05% trifluoroacetic acid, at a flow rate of 20 µL.min^−1^. After 5 min of desalting, the precolumn was switched online with the analytical C18 column (75 μm inner diameter × 50 cm; in-house packed with Reprosil C18) equilibrated in 95% solvent A (5% acetonitrile, 0.2% formic acid) and 5% solvent B (80% acetonitrile, 0.2% formic acid). Peptides were eluted using a 5–50% gradient of solvent B over 105 min at a flow rate of 300 nL.min^−1^. The mass spectrometer was operated in data-dependent acquisition mode with the Xcalibur software (version 4.3.73.11). MS survey scans were acquired with a resolution of 70,000 and an AGC target of 3e6. The 10 most intense ions were selected for fragmentation by high-energy collision induced dissociation, and the resulting fragments were analysed at a resolution of 17500, using an AGC target of 1e5 and a maximum fill time of 50 ms. Dynamic exclusion was used within 30 s to prevent repetitive selection of the same peptide.

Raw MS files were processed with the Mascot software (version 2.7.0) for database search and Proline^74^ for label-free quantitative analysis (version 2.1.2). Data were searched against Human entries of the UniProtKB protein database (release SwissProt 20201022, 20386 entries). Carbamidomethylation of cysteines was set as a fixed modification, whereas oxidation of methionine was set as variable modifications. Specificity of trypsin/P digestion was set for cleavage after K or R, and two missed trypsin cleavage sites were allowed. The mass tolerance was set to 10 ppm for the precursor and to 20 mmu in tandem MS mode. Minimum peptide length was set to 7 amino acids, and identification results were further validated in Proline by the target decoy approach using a reverse database at both a PSM and protein false-discovery rate of 1%. For label-free relative quantification of the proteins across biological replicates and conditions, cross-assignment of peptide ions peaks was enabled inside group with a match time window of 1 min, after alignment of the runs with a tolerance of +/- 600 s.

A two-tailed unpaired Student’s *t* test was then performed on log_2_-transformed values to analyse differences in protein abundance in all biologic group comparisons. The significance level was set at *P*  ≤  0.05, and ratios were considered relevant if higher than ±1. The mass spectrometry proteomics data have been deposited to the ProteomeXchange Consortium via the PRIDE^75^ partner repository with the dataset identifier PXD065390.

### IC_50_ determination

Cells were seeded in white 96-well plates (clear bottom, Revvity, Cat#6005181) at an optimal amount to maintain linear growth over the time of the assay. The following day cells were treated with or without doxycycline and with or without gemcitabine or cytarabine at an increasing range of concentration (0.01 nM, 0.1 nM, 1 nM, 5 nM, 10 nM, 50 nM, 100 nM, 500 nM, 1 µM, 10 µM, 50 µM for gemcitabine and 0.01 nM, 0.1 nM, 1 nM, 10 nM, 100 nM, 500 nM, 1 µM, 5 µM, 10 µM, 50 µM, 100 µM for cytarabine). 6 days after, cell viability was analysed using CellTiterGlo (Promega, Cat#G7572) by incubation with the cells for 15 min. Gemcitabine IC_50_ value was determined by normalising treated cells to non-treated cells and using GraphPad.

### Cell proliferation assay

Cells were seeded in white 96-well plates (clear bottom, Revvity, Cat#6005181) at an optimal amount to maintain linear growth over the time of the assay. The following day, cells were treated with or without doxycycline and with or without gemcitabine or cytarabine at the cell lines IC_50_ dose for 6 days. Cell viability was analysed every two days using CellTiterGlo (Promega, Cat#G7572) by incubation with the cells for 15 min. Cell viability was determined by normalising treated cells to non-treated cells.

### Real-time microscopy

15,000 cells were seeded in 96-wells plate in technical triplicates. The day after ZsGreen VLPs were added as described in the cell transduction section. Once the media was removed, the plate was placed in the Incucyte Zoom for confluence and ZsGreen intensity (GCUxµm²/Image) analysis for 48 h.

### Cell counting

300,000 AsPC-1 or 100,000 of PC-9 cells were seeded in 60 mm dish. Ctl or CDA biodegrader VLPs were added at 10 pg p24/cell or not (-) on day 1 and 3. Cells were also treated with or without gemcitabine on day 1 at the IC_50_ value of each cell line (80 nM for PC-9 and 100 nM for AsPC−1 cells). On day 6 (i.e. endpoint), viable cells were counted with trypan blue and a haematocytometer (Neubauer Hemocytometer).

### Protein expression and purification

VHHs and CDA were expressed in BL21 (DE3) Escherichia coli (New England Biolabs). VHHs cell pellets were resuspended in 50 mM Na_2_HPO_4_, 300 mM NaCl supplemented with 1X cOmplete, EDTA free protease inhibitor cocktail (Roche, Cat#11873580001), 0.1 mg.mL^−1^ DNAse I (Roche, Cat#10104159001) and 1 mg.mL^−1^ lysozyme (Thermo Fisher Scientific, Cat#90082). Following sonication, the sample was centrifuged at 15,000 ×*g* at 4 °C and proteins batch purified from the supernatant by cOmpleteHis-Tag Purification Resin (Roche, Cat#05893682001). VHHs were eluted in 50 mM Na_2_HPO_4_, 500 mM NaCl and 300 mM imidazole. Finally, VHHs were dialysed against 1x PBS, 20% glycerol using Slide A lysed mini dialysis devices with a 10 kDa cut-off (Thermo Fisher Scientific, Cat#88404).

CDA cell pellet was resuspended in 50 mM Tris-HCl, 150 mM NaCl, 5 mM MgCl_2_, 0.1% triton X-100 supplemented with 1X cOmplete, EDTA free protease inhibitor cocktail (Roche, Cat#11873580001), 0.1 mg.mL^−1^ DNAse I (Sigma, Cat#10104159001) and 1 mg.mL^−1^ lysozyme (Thermo Fisher Scientific, Cat#90082). Following sonication, the sample was centrifuged at 15,000 × *g* at 4 °C and proteins batch purified from the supernatant by Strep-Tactin Superflow resin (IBA, Cat#2-1206-025). CDA was eluted in 50 mM Tris-HCl, 150 mM NaCl, 5 mM MgCl_2_ and 10 mM biotin. Finally, CDA was dialysed against 50 mM Tris-HCl, 150 mM NaCl, 5 mM MgCl_2_, 15% glycerol using Slide A lysed mini dialysis devices with a 10 kDa cut-off (Thermo Fisher Scientific, Cat#88404).

### Affinity measurement

All binding studies based on SPR technology were performed on BIAcore T200 optical biosensor instrument (GE Healthcare) at 4 °C. Capture of VHH-6xHis was performed on a nitrilotriacetic acid (NTA) sensorchip in HBS-P+ buffer (10 mM Hepes pH7.4, 150 mM NaCl, and 0.05% surfactant P20) (GE Healthcare). The four flow cells (FC) of the sensorchip were used: one (FC 1) to monitor nonspecific binding with a control VHH (anti-RHO GTPase VHH, RH29^72^) and to provide background corrections for analyses and two flow cells (FC 2, 3) containing immobilised VHH-6xHis for measurement.

For immobilisation strategies, the four flow cells were loaded with nickel solution (10 mL.min^−1^ for 60 s) in order to saturate the NTA surface with Ni^2+^ and an extra wash using running buffer containing 3 mM EDTA after the nickel injection. Each His-tagged VHH in running buffer was injected in flow cells at a flow-rate of 10 mL.min^−1^. The total amount of immobilised VHH-6xHis was 250–300 resonance units (RUs; 1 RU corresponds approximately to 1 pg.mm^−2^ of protein on the sensorchip). A Single-Cycle Kinetics (SCK) analysis to determine association (on-rates), dissociation (off-rates) and affinity constants (*k*_on_, *k*_off_ and *K*_D_ respectively) was carried out. SCK method prevents potential inaccuracy due to sensorchip regeneration between cycles. SCK binding parameters are evaluated for each injection according to the tools and fit models of the BIAevaluation software. VHH were captured on the sensorchip while the recombinant CDA was used as analyte. Analyte was injected sequentially with increased concentrations ranging between 3.125 nM and 50 nM or 15.62 nM and 250 nM in a single cycle without regeneration of the sensorship between injections. Binding parameters were obtained by fitting the overlaid sensorgrams with the 1:1. Langmuir binding model of the BIAevaluation software version 2.1.

### dFdU quantification by UPLC-MS/MS analysis

#### Chemical and reagent

High-performance liquid chromatography (HPLC) grade acetonitrile (ACN) was purchased from Biosolve chimie (Dieuze, France). Vial with cap and presplit PTFE/Silicone septa were purchased from Waters (Saint-Quentin en Yvelines, France).

#### Instrumentation and chromatographic conditions

Quantitative analysis of gemcitabine and its metabolite products was performed using a Waters Acquity UPLC-MS/MS system (Saint-Quentin-en-Yvelines, France). Chromatographic separation was performed on an Acquity UPLC BEH C18 column (1.7 μm, 2.1 mm × 50 mm; Waters, Milford, MA, USA) maintained at 40 °C. The mobile phase consisted of a gradient elution with phase A (m) and phase B (ACN) at a flow rate of 0.3 mL.min^−1^. The gradient started at 85% B, was maintained for 0.3 min, and then increased to 99% B for 1.3 min before returning to the initial proportions for a total runtime of 3 min. The injection volume was 5 μL.

#### Mass spectrometric conditions

Detection was performed in electrospray positive ionisation (ESI) mode. Source and desolvation temperatures were set at 150 °C and 350 °C, respectively. Cone and desolvation gas flows were maintained at 0 L.h^−1^ and 650 L.h^−1^. Cone voltage was set at 20 V, and collision energy at 3 V. Multiple reaction monitoring (MRM) mode was used for quantification, MS collision parameters are summarised in Supplementary Table 1.

A Waters UPLC Acquity H-Class system coupled with a Xevo TQ-S micro detector and MassLynx (version 4.2) software was used for mass spectra acquisition.

#### Samples preparation and extraction procedure

450,000 AsPC-1 cells were seeded in 6-well plates (2 wells per condition). 24 h later cells were treated or not with doxycycline and with 40 nM gemcitabine for 72 h. At endpoint, cells were lysed, sonicated and protein quantified as indicated in the Western blot analysis section. Supernatants were also collected, centrifuged at 1500 × *g* for 10 min at 4 °C and treated with 50 µM THU and stored at −20 °C prior sample extraction. Sample extraction was performed using acetonitrile (ACN). Briefly, 250 µL of sample was mixed with five volumes of ACN (1250 µL) containing the internal standard (IS) at a final concentration of 20 ng.mL^−1^. The IS was added to all samples to monitor extraction efficiency and normalise analyte responses. Responses were calculated as the ratio of the analyte peak area (area under the curve, AUC) to that of the corresponding IS.

Samples were subjected to an orbital vortex-mixing sequence consisting of 5 min of vigorous mixing, followed by 5 min of rest and a further 5 min of vigorous mixing. Samples were then centrifuged at 1500 × *g* for 10 min at 4 °C. Subsequently, 840 µL of supernatant was transferred to a clean vial and evaporated to dryness under a nitrogen stream at 37 °C. The dried residue was reconstituted in 140 µL of ACN, and 5 µL was injected into the UPLC-MS/MS system for analysis.

### Animal experimentation

Experimental procedures performed on mice were approved by the ethical committee of INSERM CREFRE US006 animal facility and authorised by the French Ministry of Research: APAFIS #57814-2025092411253403 v9. Animals were housed under controlled temperature and lighting (a 12 h light/dark cycle). The maximal tumour size/burden authorised was 1000 mm^3^.

#### In vivo experiments using doxycycline-inducible CDX models

5 × 10^6^ BxPC-3 or H441 stable cell lines expressing the Ctl/CDA biodegraders were resuspended in 100 µL of PBS with 50% of Matrigel (Corning, Cat#356231) and injected subcutaneously into the left flank of 6 to 8-week-old female NSG mice (Charles River Laboratories); thus, sex-disaggregated data were not applicable. Mice were fed with normal diet and water until their subcutaneous tumour reached 200 mm^3^, approximately 30 days after injection. The mice were divided into groups of 4/5 mice (exact number indicated in the corresponding figure legends). Mice were treated twice a week ± doxycycline via drinking water (2 mg.mL^−1^ in 20% sucrose) and ± gemcitabine at 40 mg.kg^−1^ for BxPC-3 CDX model and 25 mg.kg^−1^ for H441 CDX model. Note on the first day of doxycycline treatment, mice were injected with 100 μL of 4 mg.mL^−1^ of doxycycline in sterile 0.9% aqueous NaCl by intraperitoneal injection. Tumours were measured with a digital callipers (Thermo Fisher Scientific) and tumour volume was calculated using the formula *V* = (Width^2^ × Length)/2. Mice were humanly sacrificed and tumours were resected for analysis after 2 weeks of treatment for H441 CDX model and at the end of the experiment for BxPC-3 CDX model.

#### In vivo experiments using FlashRNA^®^ VLPs-transduced CDX model

5 × 10^6^ PC-9 cell lines were resuspended in 100 µL of PBS with 50% of Matrigel (Corning, Cat#356231) and injected subcutaneously into the left flank of 6 to 8-week-old female NSG mice (Charles River Laboratories); thus, sex-disaggregated data were not applicable. Mice were fed with normal diet and water until their subcutaneous tumour reached 150 mm^3^, approximately 15 days after injection.

##### Firefly Luciferase mRNA-loaded FlashRNA^*®*^ VLPs ([VLPs] = 243 µg p24/mL)

For dose-response experiments, three mice were treated for 6 h with 10, 25, or 50 µL of VLPs (i.e. 2.43, 6.08 and 12.2 µg p24, respectively). For time-response experiments, three mice received 50 µL of VLP by intratumoral injection and were analysed after 6, 24, or 48 h. All images were acquired after intraperitoneal injection of 150 µL of D-luciferin (stock solution at 30 mg.mL^−1^ in DPBS; PerkinElmer), corresponding to a dose of 150 mg.kg^−1^ body weight. Bioluminescence images (BLI) were acquired 5 min after D-luciferin injection. Mice were then humanely sacrificed, and organs (lung, liver, spleen, kidneys, and heart) as well as tumours were resected for immediate ex vivo bioluminescence analysis. Image analysis and bioluminescence quantification were performed using Living Image software (PerkinElmer).

##### Biodegraders mRNA-loaded FlashRNA^*®*^ VLPs ([Ctl biodegrader VLPs] = 152 µg p24/mL or [CDA biodegrader VLPs] = 125 µg p24/mL)

Four mice were treated twice a week with 50 µL of VLPs by intratumoral injection (i.e. 7.6 µg p24 of Ctl biodegrader VLPs and 6.25 µg p24 of CDA biodegrader VLPs), with or without gemcitabine (40 mg.kg^−1^). Body weight was monitored twice a week, as well as tumour size that was measured using digital callipers (Thermo Fisher Scientific). Tumour volume was calculated as specified above. After 2 weeks of treatment, mice were humanely sacrificed, and tumours resected for Western blot analysis.

### Quantification and statistical analysis

The quantifications were performed using Image Lab (version 6.1, Bio-Rad), Prism 10.0 (GraphPad Software), FlowJo v10 Software or Living Image (Revvity). BRET titration curves, IC_50_ determination, proliferation curves and statistical analysis were performed using Prism 10.0 (GraphPad Software). The data are typically presented as mean ± SD or SEM as specified in the figure legends. Statistical analyses were performed with one-way ANOVA followed by Dunnett’s post hoc tests or two-tailed unpaired Student’s *t* test, as indicated in the figure legends. ns not significant.

### Reporting summary

Further information on research design is available in the Nature Portfolio Reporting Summary linked to this article.

## Supplementary information

Supplementary Information

Reporting Summary

Transparent Peer Review file

## Source data

Source data file

## Data Availability

The mass spectrometry proteomics data generated in this study have been deposited in the ProteomeXchange Consortium database via the PRIDE partner repository with the accession code PXD065390. The remaining data are available within the Article, Supplementary Information or Source Data file. Source data are provided with this paper.
