# Supplementary material for: A cytidine deaminase biodegrader sensitises multiple cancer types to deoxycytidine analogue-based chemotherapies
Source: Nat Commun. 2026 Oct 7;17:11402. doi: 10.1038/s41467-026-78315-0 (PMC13737281; doi:10.1038/s41467-026-78315-0)
Supplement: Supplementary file 1 [file 41467_2026_78315_MOESM1_ESM.pdf]

Supplementary Information

A cytidine deaminase biodegrader sensitises multiple cancer types to deoxycytidine analogue-based chemotherapies

Sorbara, M *et al*.

a

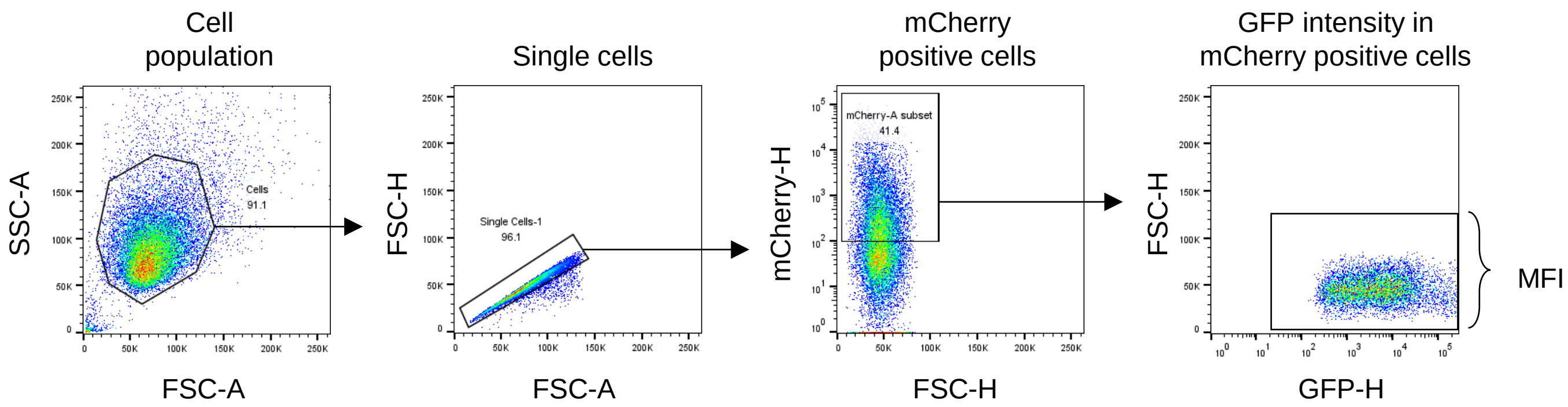

b

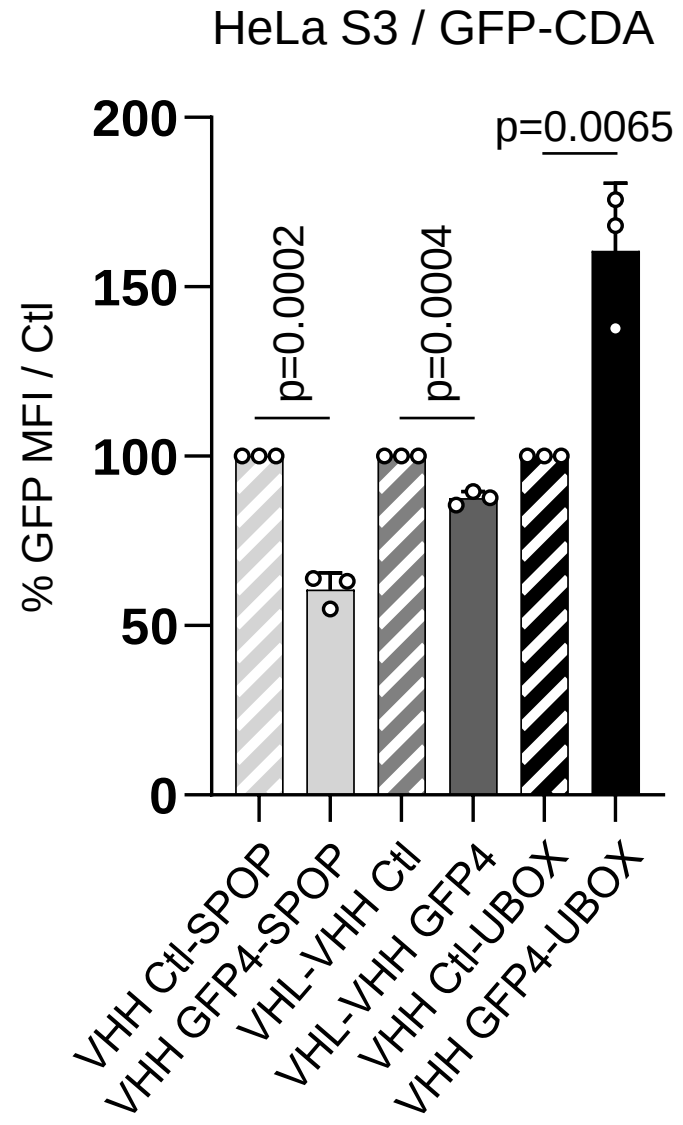

c

# clone	163	12	78	177	192	21	39	73	118	125	148	168	174	Total 13
# repetition	10	6	2	2	2	1	1	1	1	1	1	1	1	30

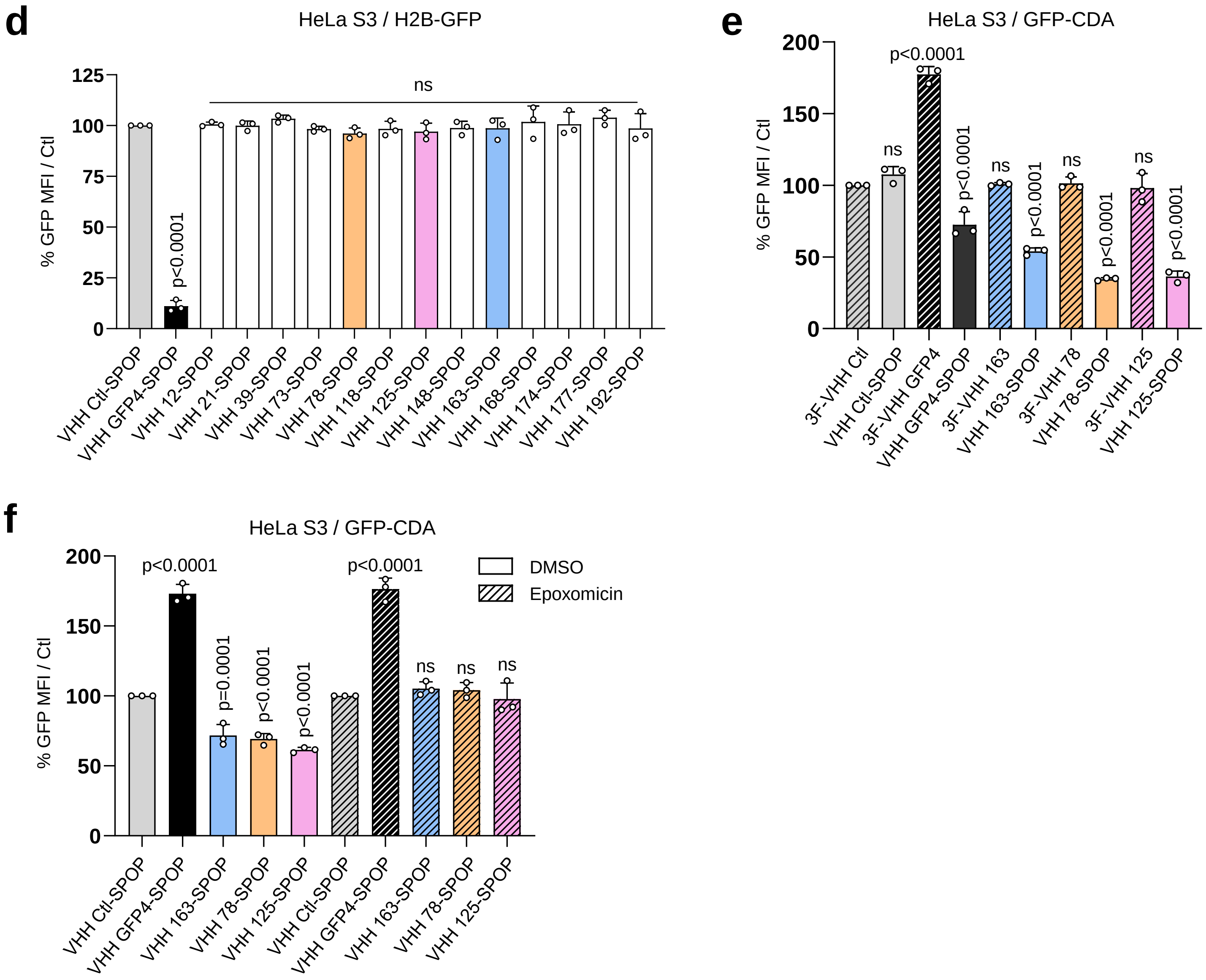

Supplementary Figure 1: Characterisation of the positive hits from the cell-based screening. (**a**) Gating strategy for the acquisition of GFP fluorescence-based flow cytometry data. FSC: forward scatter; SSC: side scatter; MFI: median of fluorescence intensity. (**b**) VHH Ctl and VHH GFP4 were fused to three commonly used E3 ubiquitin ligases (VHL, SPOP, UBOX) and transfected for 48 h in HeLa S3 / GFP-CDA cells. GFP MFI was quantified by flow cytometry in transfected cells and normalised to Ctl GFP MFI. (**c**) The 29 positive hits from the screening were sequenced and 13 unique clones were identified. (**d**) Indicated biodegraders were transfected for 48 h in HeLa S3 / H2B-GFP control cells. GFP MFI was quantified by flow cytometry in transfected cells and normalised to Ctl GFP MFI. (**e**) Indicated VHHs were either fused to 3xFLAG (3F) or to SPOP and transfected for 48 h in HeLa S3 / GFP-CDA cells. GFP MFI was quantified by flow cytometry in transfected cells and normalised to 3F-VHH Ctl GFP MFI. (**f**) Biodegraders were transfected in HeLa S3 / GFP-CDA cells and treated for 18 h with 0.8 μM of the proteasome inhibitor epoxomicin or DMSO. GFP MFI was quantified by flow cytometry in transfected cells and normalised to each Ctl GFP MFI. Statistical significance was determined by a two-tailed unpaired Student's t-test in (**b**) and by one-way ANOVA followed by Dunnett post-hoc tests in (**d-f**) (ns, not significant). Error bars in (**b**, **d-f**) correspond to mean values ± SD from three independent biological repeats. Each experiment was performed three times (**b**, **d-f**) as biological replicates. Source data are provided as a Source Data file.

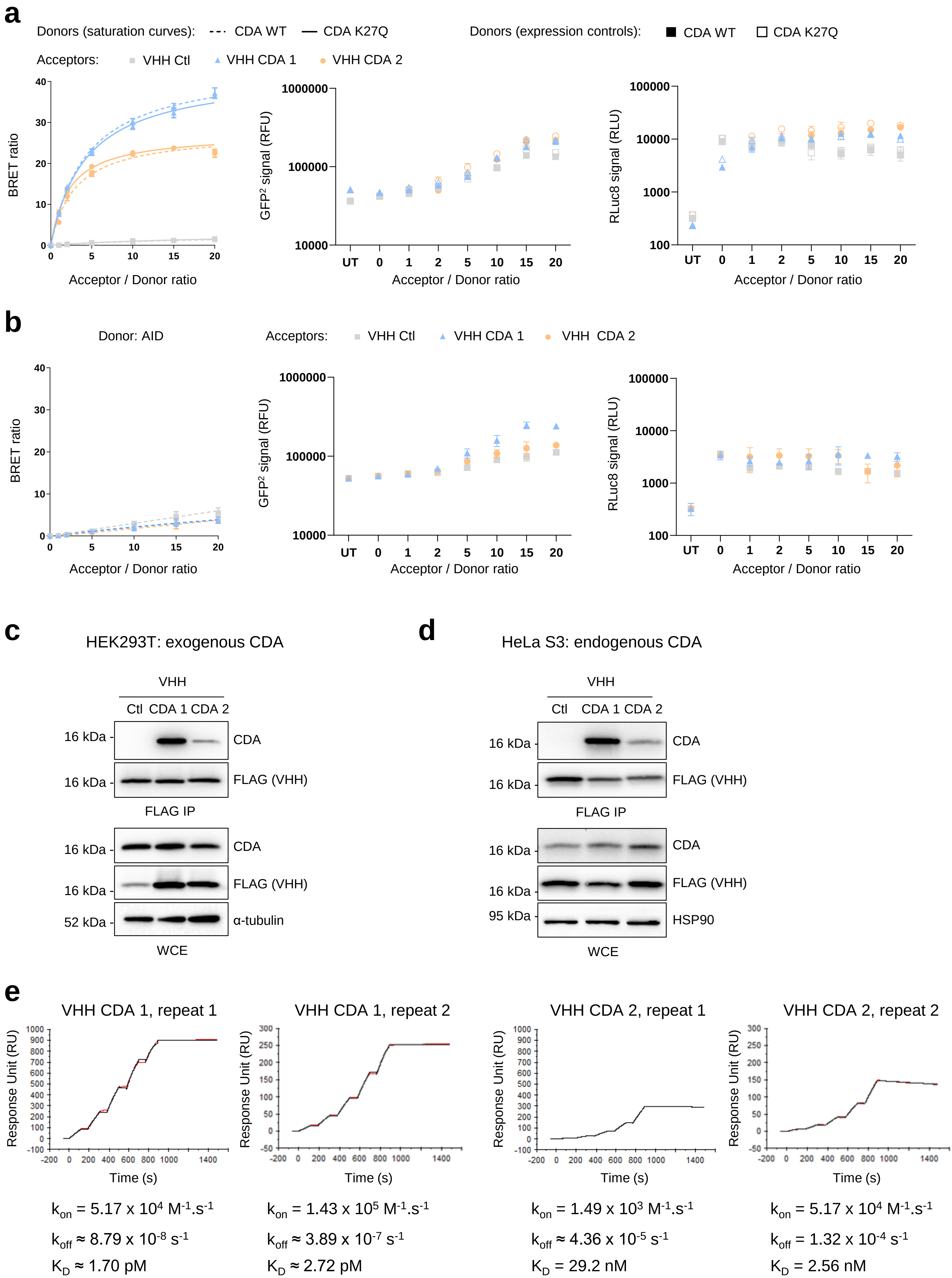
a
Donors (saturation curves): CDA WT CDA K27Q
Donors (expression controls): CDA WT CDA K27Q
Acceptors: VHH Ctl VHH CDA 1 VHH CDA 2
BRET ratio
GFP² signal (RFU)
RLuc8 signal (RLU)
Acceptor / Donor ratio
UT 0 1 2 5 10 15 20
b
Donor: AID
Acceptors: VHH Ctl VHH CDA 1 VHH CDA 2
BRET ratio
GFP² signal (RFU)
RLuc8 signal (RLU)
Acceptor / Donor ratio
c
HEK293T: exogenous CDA
VHH
Ctl CDA 1 CDA 2
16 kDa CDA
16 kDa FLAG (VHH)
FLAG IP
16 kDa CDA
16 kDa FLAG (VHH)
52 kDa α-tubulin
WCE
d
HeLa S3: endogenous CDA
VHH
Ctl CDA 1 CDA 2
16 kDa CDA
16 kDa FLAG (VHH)
FLAG IP
16 kDa CDA
16 kDa FLAG (VHH)
95 kDa HSP90
WCE
e
VHH CDA 1, repeat 1
VHH CDA 1, repeat 2
VHH CDA 2, repeat 1
VHH CDA 2, repeat 2
Response Unit (RU)
Time (s)
k_on = 5.17 x 10^4 M^-1.s^-1
k_off ≈ 8.79 x 10^-8 s^-1
K_D ≈ 1.70 pM
k_on = 1.43 x 10^5 M^-1.s^-1
k_off ≈ 3.89 x 10^-7 s^-1
K_D ≈ 2.72 pM
k_on = 1.49 x 10^3 M^-1.s^-1
k_off ≈ 4.36 x 10^-5 s^-1
K_D = 29.2 nM
k_on = 5.17 x 10^4 M^-1.s^-1
k_off = 1.32 x 10^-4 s^-1
K_D = 2.56 nM

Supplementary Figure 2: Characterisation of anti-CDA VHHs. (**a**) BRET donor saturation assay between CDA WT or CDA K27Q (donors) and the VHH Ctl (grey), CDA 1 (blue) and CDA 2 (orange) (acceptors) with the corresponding GFP^2 and RLuc8 signals. (**b**) BRET donor saturation assay between Activated Induced CDA (AID) as donor and the VHHs as acceptors with the corresponding GFP^2 and RLuc8 signals. (**c**, **d**) Co-immunoprecipitation of 3xFLAG-VHH Ctl, 3xFLAG-VHH CDA 1 and 3xFLAG-VHH CDA 2 with overexpressed CDA in HEK293T cells (**c**) or with the endogenously expressed CDA in HeLa S3 cells (**d**). IP: immunoprecipitation, WCE: whole-cell extract. (**e**) Single cycle kinetics (SCK) surface plasmon resonance (SPR) measurements on VHH CDA 1- and VHH CDA 2-6Hix-myc-6xHis captured on Ni-NTA chip (nickel affinity capture of 6xHis). k_{on}, k_{off} and K_D kinetic parameters are displayed below each corresponding sensorgram. Error bars are mean ± SEM (**a**) or SD (**b**) of biological repeats. Each experiment was performed twice (**e**) or three times (**a-d**) as biological replicates. Source data are provided as a Source Data file.

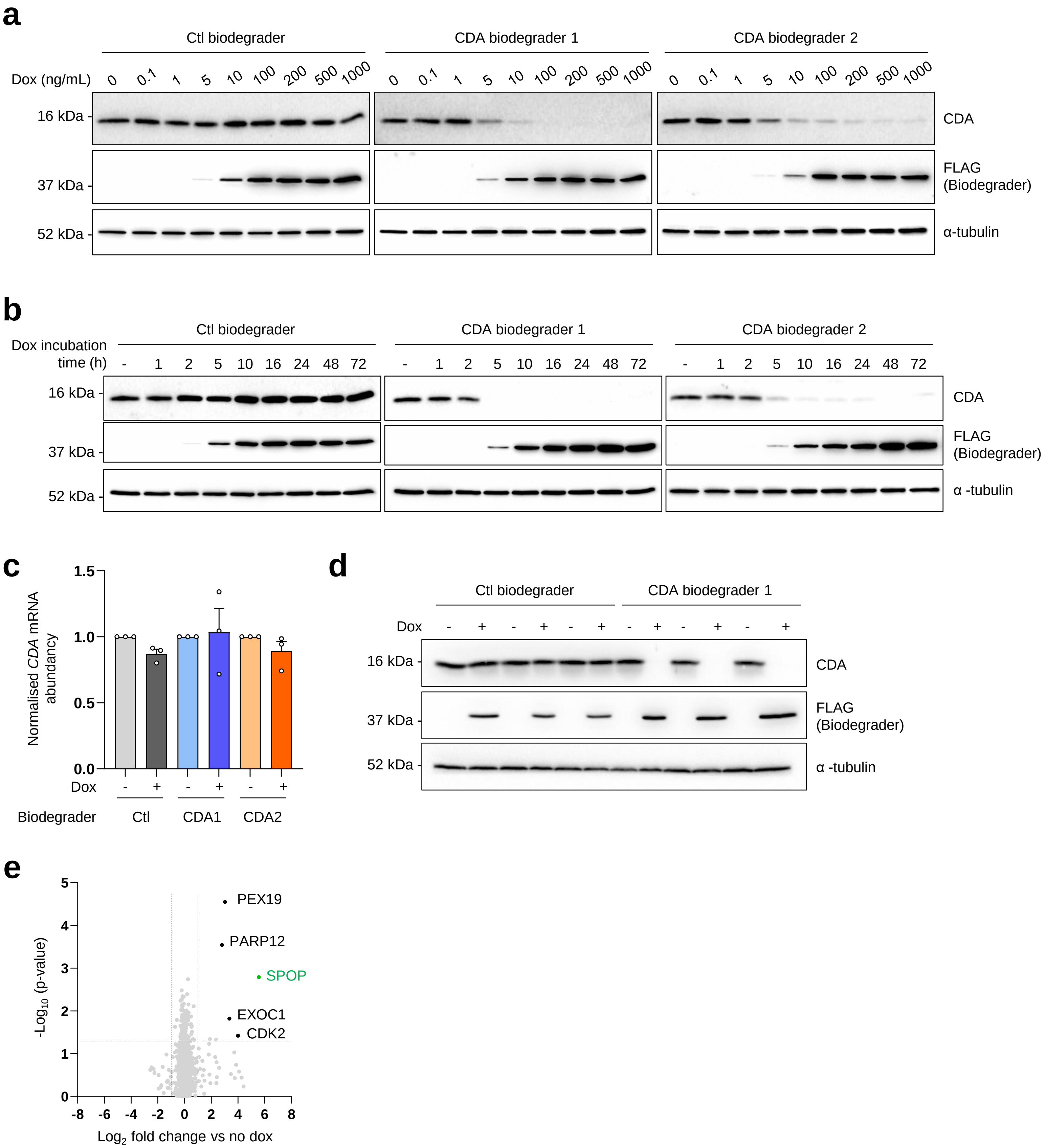

Supplementary Figure 3: Characterisation of CDA biodegraders in AsPC-1 pancreatic cancer cells. (**a**) Western blot analysis of the dose response experiment with the indicated doxycycline (dox) concentrations incubated for 48 h in AsPC-1 cells expressing either Ctl biodegrader or CDA biodegrader 1 or 2. (**b**) Western blot analysis of the time response experiment of AsPC-1 cells expressing the same biodegraders as in (**a**) after addition of 0.5 µg.mL^{-1} of dox. (**c**) Quantitative real-time PCR was performed after 48 h of treatment with 0.5 µg.mL^{-1} of dox (+) or untreated (-) in AsPC-1 stable cell lines. No dox conditions were standardised to a value of 1.0 and CDA mRNA abundancy is represented as a fold-change relative to that value. (**d**) Western blot analysis of the three replicates used for mass spectrometry analysis shown in Figure 2 and in (**e**). The expression of the biodegraders is detected with a FLAG antibody. α-tubulin is the loading control. (**e**) Whole cell proteomics mass spectrometry analysis of AsPC-1 cells expressing Ctl biodegrader (10 h of dox treatment at 0.5 µg.mL^{-1}) or not (no dox). P-values were determined by a two-tailed unpaired Student's t-test. Significantly altered proteins (P<0.05, $\log_2$ fold change < -1 or > 1) are indicated as black dots and SPOP in green on the volcano plot. Error bars are mean ± SD of biological repeats. Error bars in (**c**) correspond to mean values ± SEM from biological repeats. Each experiment was performed with three biological replicates (**a-e**). Source data are provided as a Source Data file.

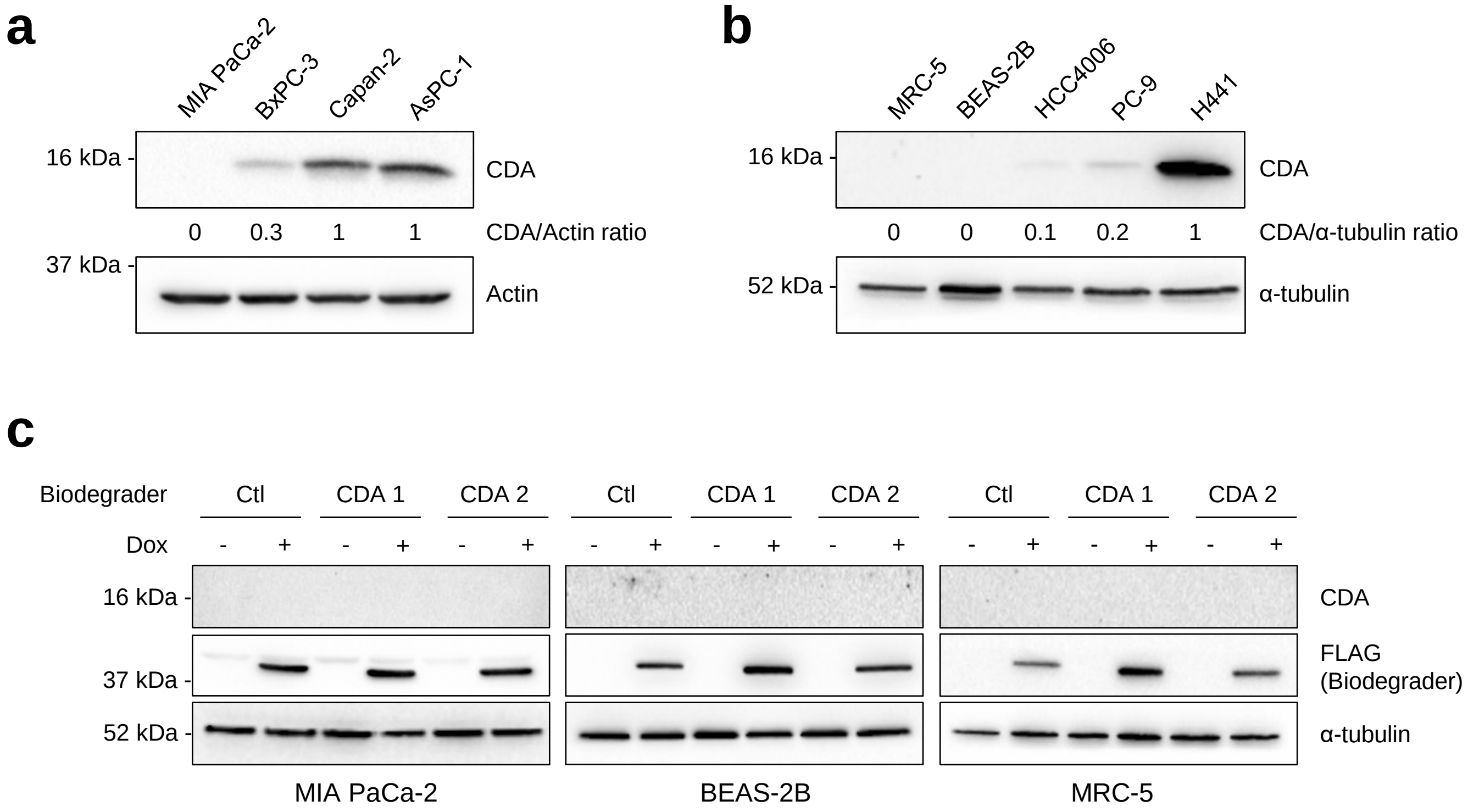

Supplementary Figure 4: CDA biodegraders effect on cells that do not express CDA. (**a**, **b**) Western blot analysis of CDA protein level in pancreatic cancer cells (**a**): MIA PaCa-2, AsPC-1 BxPC-3 and Capan-2 and in lung cells (**b**): non-cancerous MRC-5 and BEAS-2B and cancer cell lines HCC4006, PC-9 and H441. Actin or α-tubulin is the loading control. CDA protein level is normalised to loading control and is indicated underneath each corresponding lane. (**c**) Western blot analysis of CDA and biodegraders' expression in MIA PaCa-2, BEAS-2B and MRC-5 cells after 48 h of induction with 0.5 µg.mL^{-1} of dox. Each experiment in (**a-c**) was performed three times (biological repeats). Source data are provided as a Source Data file.

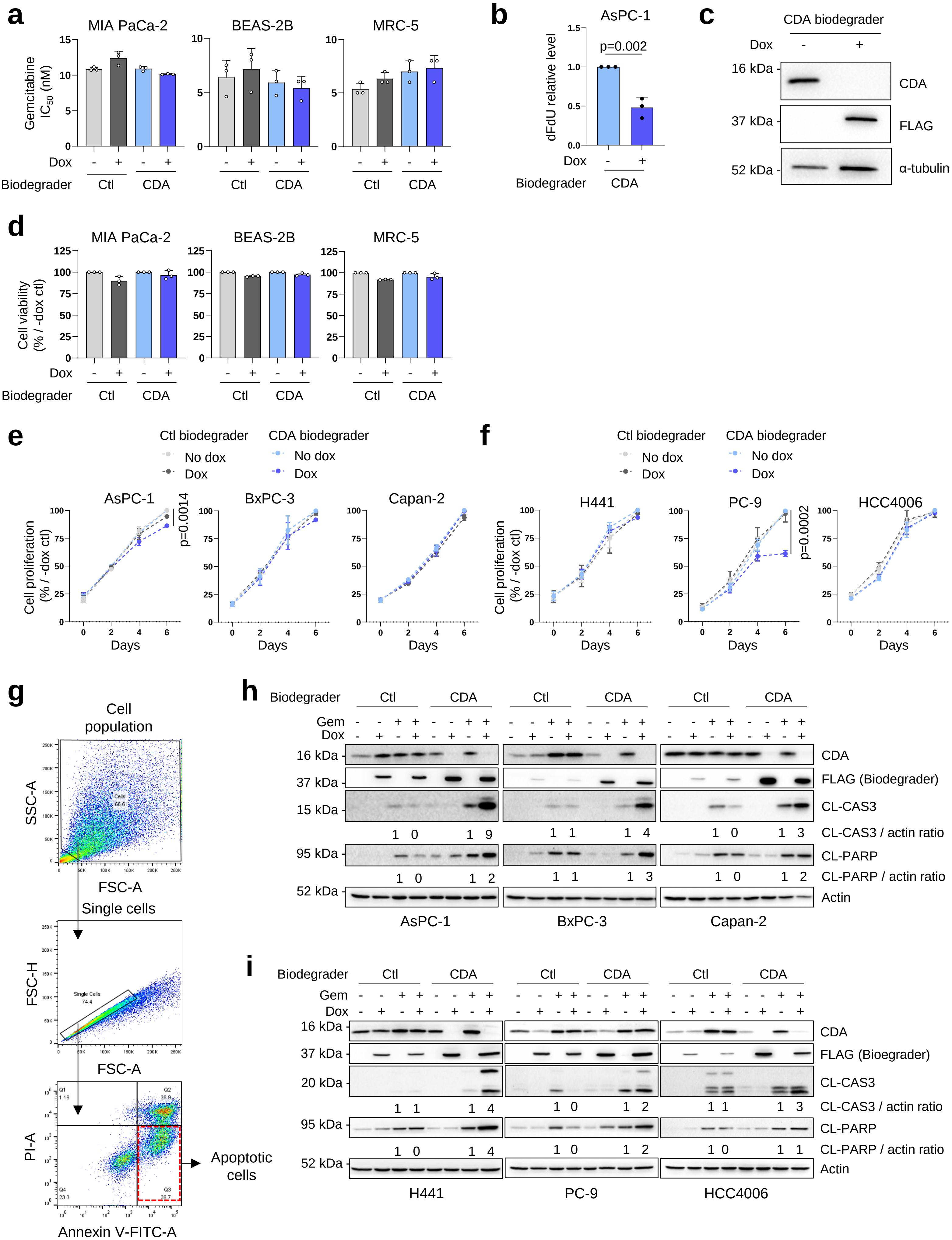

a
MIA PaCa-2
BEAS-2B
MRC-5
Gemcitabine IC50 (nM)
Dox
Biodegrader
Ctl
CDA
b
AsPC-1
p=0.002
dFdU relative level
Dox
Biodegrader
CDA
c
CDA biodegrader
Dox
16 kDa
37 kDa
52 kDa
CDA
FLAG
α-tubulin
d
MIA PaCa-2
BEAS-2B
MRC-5
Cell viability (% / -dox ctl)
Dox
Biodegrader
Ctl
CDA
e
Ctl biodegrader
No dox
Dox
CDA biodegrader
No dox
Dox
AsPC-1
BxPC-3
Capan-2
p=0.0014
Cell proliferation (% / -dox ctl)
Days
f
Ctl biodegrader
No dox
Dox
CDA biodegrader
No dox
Dox
H441
PC-9
HCC4006
p=0.0002
Cell proliferation (% / -dox ctl)
Days
g
Cell population
SSC-A
FSC-A
Single cells
FSC-H
FSC-A
PI-A
Annexin V-FITC-A
Apoptotic cells
h
Biodegrader
Ctl
CDA
Gem
Dox
16 kDa
37 kDa
15 kDa
95 kDa
52 kDa
CDA
FLAG (Biodegrader)
CL-CAS3
CL-CAS3 / actin ratio
CL-PARP
CL-PARP / actin ratio
Actin
AsPC-1
BxPC-3
Capan-2
i
Biodegrader
Ctl
CDA
Gem
Dox
16 kDa
37 kDa
20 kDa
95 kDa
52 kDa
CDA
FLAG (Bioegrader)
CL-CAS3
CL-CAS3 / actin ratio
CL-PARP
CL-PARP / actin ratio
Actin
H441
PC-9
HCC4006

Supplementary Figure 5: Characterisation of CDA biodegrader effect on cell proliferation and apoptosis. (**a**) The control cell lines, i.e. which do not express CDA, MIA PaCa-2, BEAS-2B and MRC-5 expressing either the Ctl or CDA biodegrader (+ dox) or not (- dox) were treated for 6 days with a range of gemcitabine concentrations. The IC_{50} values of gemcitabine were determined at day 6 with the Cell Titer Glo assay for each cell line. (**b**) dFdU level in AsPC-1 cells supernatant treated 72 h with gemcitabine (40 nM) ± doxycycline was quantified by UPLC-MS/MS and normalised to the no dox (-) condition. (**c**) Western blot analysis of the corresponding cells from (**b**). (**d**) MIA PaCa-2, BEAS-2B and MRC-5 cell viability determined after day 6 after expression (+) or not (-) of Ctl or CDA biodegrader by doxycycline induction. (**e**, **f**) Proliferation of AsPC-1, BxPC-3 and Capan-2 cell lines (**e**) and H441, PC-9, HCC4006 cell lines (**f**) expressing either the Ctl or CDA biodegrader (+ dox) or not (- dox) for 6 days (without gemcitabine treatment). (**g**) Gating strategy for the acquisition of cell apoptosis-based flow cytometry data. FSC: forward scatter; SSC: side scatter; PI: Propidium Iodide. (**h**, **i**) Western blot analysis of the apoptosis markers cleaved caspase 3 (CL-CAS3) and cleaved PARP (CL-PARP) in pancreatic cancer cells AsPC-1, BxPC-3 and Capan-2 (**h**) and in H441, PC-9, HCC4006 lung cancer cell lines (**i**) expressing (+ dox) or not (- dox) the indicated biodegraders after 6 days of treatment with or without gemcitabine. Actin is the loading control. CL-CAS3 and CL-PARP protein levels are normalised to the loading control and are indicated underneath each corresponding lane. Statistical significance was determined by a two-tailed unpaired Student's t-test in (**b**, **e**, **f**). Error bars indicate mean ± SD (**a**, **b**, **d**) or SEM (**e**, **f**) of biological repeats. Each experiment in (**a-f**, **h-i**) was performed three times (biological repeats). Source data are provided as a Source Data file.

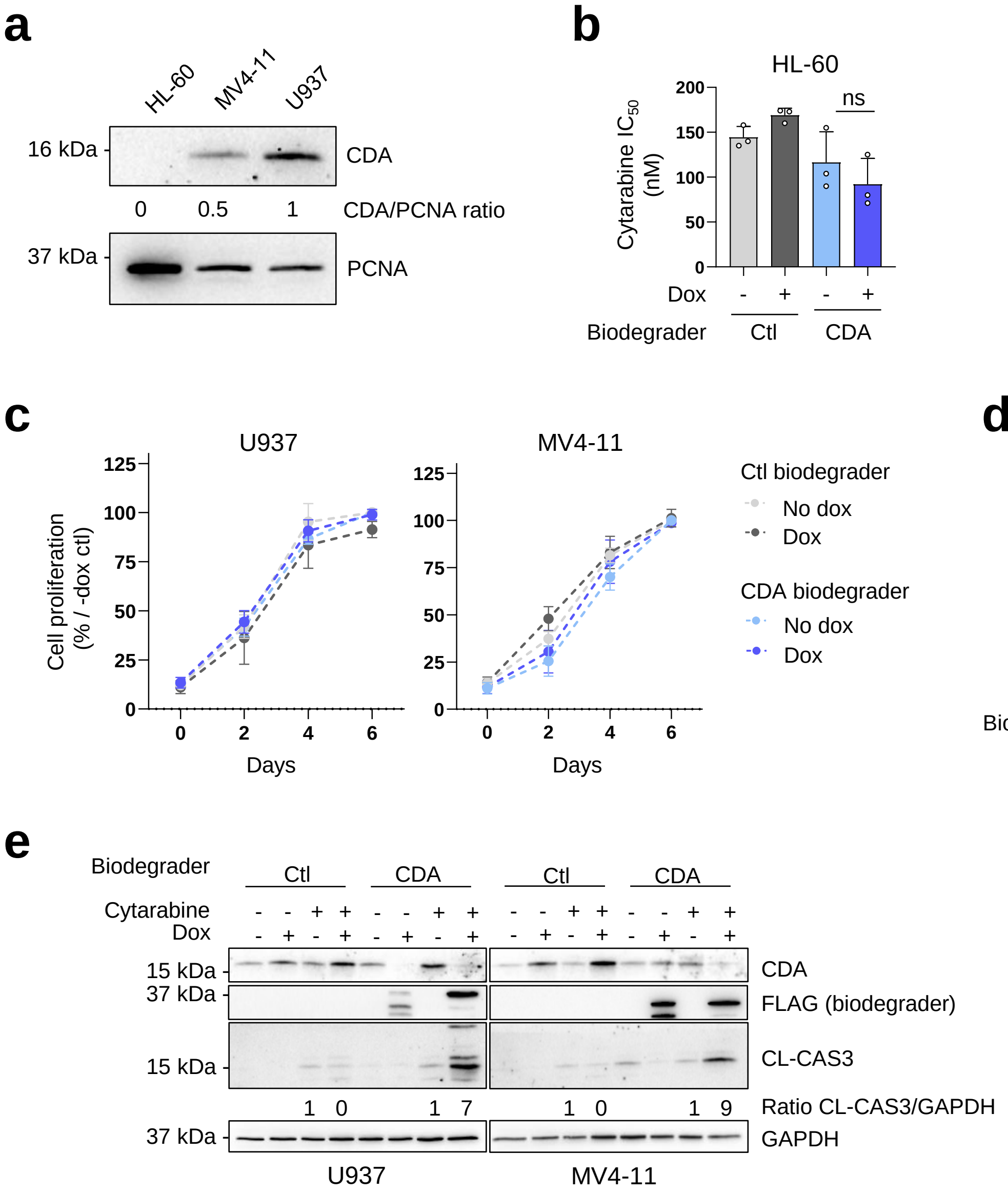

Supplementary Figure 6: Characterisation of CDA biodegrader in AML cells. (**a**) Western blot analysis of CDA protein level in AML cancer cells U937, MV4-11. PCNA is the loading control. CDA protein level is normalised to loading control and is indicated underneath each corresponding lane. (**b**) The CDA-negative HL-60 cell line expressing either the Ctl or CDA biodegrader (+ dox) or not (- dox) was treated for 6 days with a range of cytarabine concentrations. The IC_{50} of cytarabine was determined with the Cell Titer Glo assay at day 6. (**c**) Proliferation of U937 and MV4-11 cell lines expressing either the Ctl or CDA biodegrader (dox) or not (no dox) for 6 days (without cytarabine treatment). (**d**) HL-60 cell viability was determined after 6 days of expression (+) or not (-) of either the Ctl or CDA biodegrader by doxycycline induction. (**e**) Western blot analysis of the apoptosis markers cleaved caspase 3 (CL-CAS3) in U937 and MV4-11 cells expressing (+ dox) or not (- dox) the indicated biodegraders after 6 days of treatment with or without cytarabine. GAPDH is the loading control. CL-CAS3 protein levels are normalised to the loading control and are indicated underneath each corresponding lane. Statistical significance was determined by a two-tailed unpaired Student's t-test in (**b**). ns, not significant. Error bars indicate mean ± SD (**b**, **d**) or SEM (**c**) of biological repeats. Each experiment in (**a-e**) was performed three times (biological repeats). Source data are provided as a Source Data file.

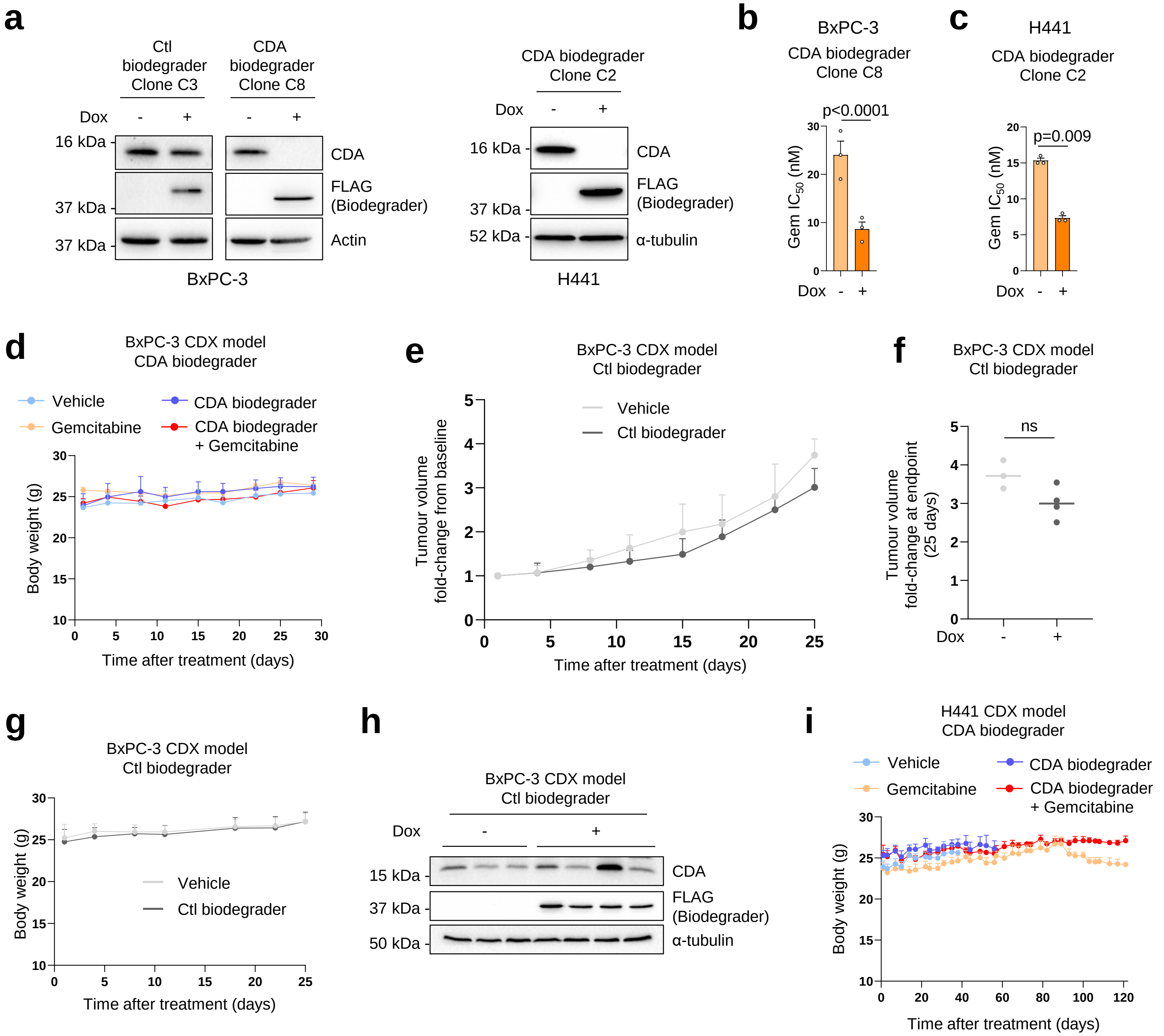

Supplementary Figure 7: Characterisation of BxPC-3 and H441 clones and effect of the Ctl biodegrader on tumour growth. (**a**) Western blot analysis of BxPC-3 and H441 clones used for the in vivo experiments after 48 h of treatment with dox (+) or not (-). Actin or α-tubulin is the loading control. (**b**, **c**) Gemcitabine IC_{50} values of BxPC-3 clone C8 (**b**) and H441 clone C2 (**c**) expressing CDA biodegrader (+ dox) or not (- dox) and treated with a range of concentrations of gemcitabine for 6 days. (**d**) Body weight of mice from experiments displayed on Figure 6a. (**e**) Tumours volume fold-change of BxPC-3 cell-derived xenograft (CDX) model. Tumours were treated with vehicle (n=3 mice), or doxycycline (2 $mg.mL^{-1}$ to induce expression of the Ctl biodegrader, n=4 mice). Tumour volumes were normalised to the first day of treatment. (**f**) Scatter dot plot showing the tumour volume fold-change from (**e**) at endpoint. Bars show the median value. (**g**) Body weight of mice from (**e**) (vehicle n=3, Ctl biodegrader n=4). (**h**) Western blot analysis of individual BxPC-3 CDX tumours from (**e**) resected at endpoint (vehicle n=3, Ctl biodegrader n=4). The expression of Ctl biodegrader is shown with the FLAG antibody. α-tubulin is the loading control. (**i**) Body weight of mice from experiments displayed on Figure 6d. Statistical significance was determined by a two-tailed unpaired Student's t-test (ns, not significant). Error bars indicate mean ± SEM (**b**, **c**, **d**, **g**, **i**) or SD (**e**). Each experiment in (**a-c**) was performed three times (biological repeats). Source data are provided as a Source Data file.

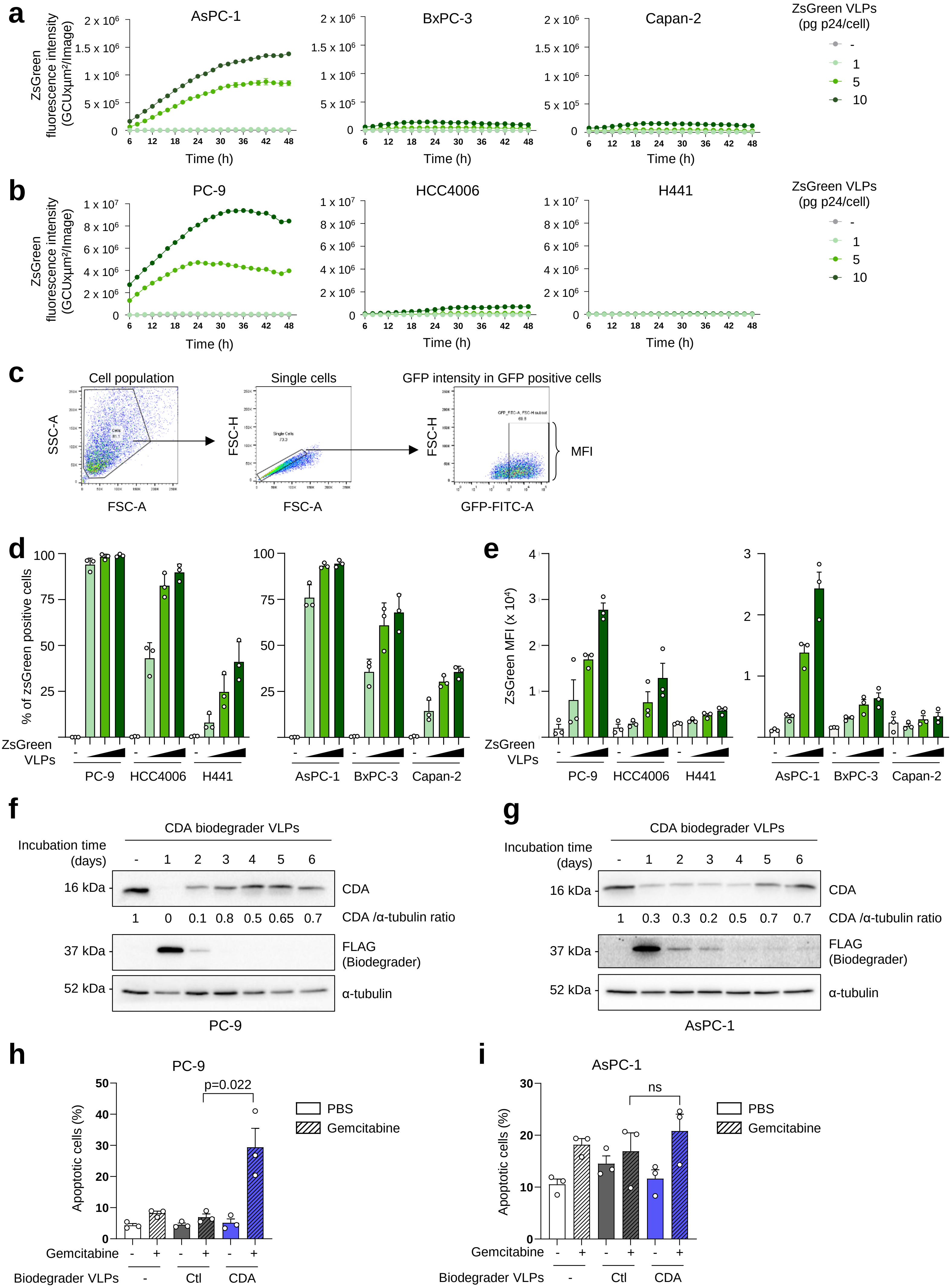

a
AsPC-1
BxPC-3
Capan-2
ZsGreen fluorescence intensity (GCUxµm²/Image)
Time (h)
ZsGreen VLPs (pg p24/cell)
-
1
5
10
b
PC-9
HCC4006
H441
ZsGreen fluorescence intensity (GCUxµm²/Image)
Time (h)
ZsGreen VLPs (pg p24/cell)
c
Cell population
Single cells
GFP intensity in GFP positive cells
SSC-A
FSC-A
FSC-H
GFP-FITC-A
MFI
d
% of zsGreen positive cells
ZsGreen VLPs
PC-9
HCC4006
H441
AsPC-1
BxPC-3
Capan-2
e
ZsGreen MFI (x 10⁴)
ZsGreen VLPs
PC-9
HCC4006
H441
AsPC-1
BxPC-3
Capan-2
f
CDA biodegrader VLPs
Incubation time (days)
- 1 2 3 4 5 6
16 kDa
CDA
1 0 0.1 0.8 0.5 0.65 0.7
CDA /α-tubulin ratio
37 kDa
FLAG (Biodegrader)
52 kDa
α-tubulin
PC-9
g
CDA biodegrader VLPs
Incubation time (days)
- 1 2 3 4 5 6
16 kDa
CDA
1 0.3 0.3 0.2 0.5 0.7 0.7
CDA /α-tubulin ratio
37 kDa
FLAG (Biodegrader)
52 kDa
α-tubulin
AsPC-1
h
PC-9
p=0.022
Apoptotic cells (%)
PBS
Gemcitabine
Gemcitabine
Biodegrader VLPs
-
Ctl
CDA
i
AsPC-1
ns
Apoptotic cells (%)
PBS
Gemcitabine
Gemcitabine
Biodegrader VLPs
-
Ctl
CDA

Supplementary figure 8: In vitro characterisation of CDA biodegrader mRNA delivery via VLPs in pancreatic and lung cancer cell lines. (**a**, **b**) The Total Green Object Integrated Intensity (GCU x µm²/Image) metric was used to quantify ZsGreen fluorescence intensity of AsPC-1, BxPC-3 and Capan-2 pancreatic cancer cells (**a**) or PC-9, HCC4006 and H441 lung cancer cells (**b**) over time after transduction with VLPs containing mRNA encoding for ZsGreen (ZsGreen VLPs) at different doses (0; 1; 5; 10 pg p24/cell), followed by real-time microscopy (Incucyte Zoom). Analysis began 6 h after VLPs addition when media was changed and continued for a total period of 48 h. (**c**) Flow cytometry gating strategy used in panels (**d**, **e**). FSC: forward scatter; SSC: side scatter; MFI: median of fluorescence intensity. (**d**) Percentage of ZsGreen-positive cells 48 h after transduction with ZsGreen VLPs at different doses (0; 1; 5; 10 pg p24/cell), as analysed by flow cytometry. (**e**) ZsGreen MFI of lung and pancreatic cancer cells determined by flow cytometry 48 h after transduction with ZsGreen VLP at different doses (0; 1; 5 and 10 pg p24/cell). (**f**, **g**) Kinetic experiments analysed by Western blotting. Indicated cells were transduced or not (-) once with 10 pg p24/cell of Ctl or CDA biodegrader VLPs on day 0; CDA and biodegraders (FLAG) protein level was determined in PC-9 (**f**) and AsPC-1 cells (**g**) every day for 6 days. α-tubulin is the loading control. Quantification of CDA ratio normalised to the loading control is indicated underneath each condition. (**h**, **i**) Percentage of apoptotic cells after 6 days of treatment with (crossed bars) or without gemcitabine and with or without transduction with Ctl or CDA biodegrader VLPs in PC-9 (**h**) and AsPC-1 cells (**i**). Statistical significance was determined by a two-tailed unpaired Student's t-test. ns, not significant. Error bars indicate mean ± SEM (**a**, **b**, **d**, **e**, **h**, **i**). Each experiment in (**a, b, d, e, h, i**) was performed independently three times and twice in **f, g** (biological replicates). Source data are provided as a Source Data file.

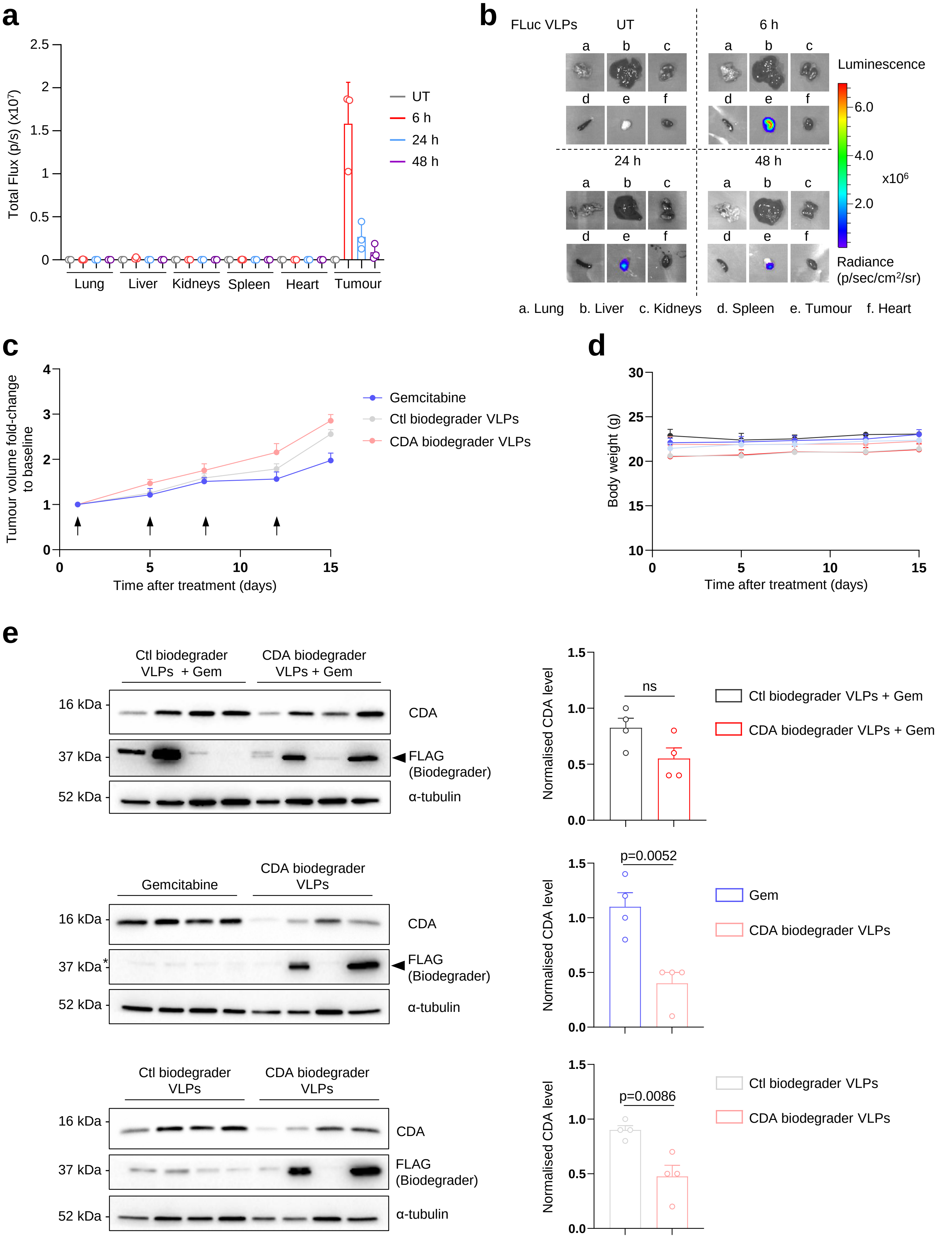

a
Total Flux (p/s) (x10⁷)
2.5
2
1.5
1
0.5
0
UT
6 h
24 h
48 h
Lung
Liver
Kidneys
Spleen
Heart
Tumour
b
FLuc VLPs
UT
6 h
24 h
48 h
a b c
d e f
Luminescence
6.0
4.0
2.0
x10⁶
Radiance
(p/sec/cm²/sr)
a. Lung
b. Liver
c. Kidneys
d. Spleen
e. Tumour
f. Heart
c
Tumour volume fold-change to baseline
Gemcitabine
Ctl biodegrader VLPs
CDA biodegrader VLPs
Time after treatment (days)
d
Body weight (g)
Time after treatment (days)
e
Ctl biodegrader VLPs + Gem
CDA biodegrader VLPs + Gem
16 kDa
37 kDa
52 kDa
CDA
FLAG
(Biodegrader)
α-tubulin
Normalised CDA level
ns
Ctl biodegrader VLPs + Gem
CDA biodegrader VLPs + Gem
Gemcitabine
CDA biodegrader VLPs
37 kDa*
p=0.0052
Gem
CDA biodegrader VLPs
Ctl biodegrader VLPs
CDA biodegrader VLPs
p=0.0086
Ctl biodegrader VLPs
CDA biodegrader VLPs

Supplementary figure 9: In vivo characterisation of CDA biodegrader mRNA delivery via VLPs in a lung CDX model. (**a**, **b**) FLuc expression was assessed ex vivo by bioluminescence imaging in tumours and indicated organs, 6, 24 and 48 h after intratumoral injection of 12.2 µg p24 of FLuc mRNA-loaded VLPs and compared to untreated mice (UT, n=3 mice per group). Total flux quantification (i.e. luminescent signal) is shown in (**a**) and representative images of each organ and tumour at the different time points are displayed in (**b**). (**c**) Tumours volume fold-change from PC-9 CDX model. Tumours (n=4 mice per group) were treated with gemcitabine (40 $mg.kg^{-1}$), Ctl biodegrader VLPs or CDA biodegrader VLPs. Arrows indicate the injection times of the treatments (VLPs and/or gemcitabine). Tumours were normalised to the first day of treatment. (**d**) Body weight of mice from (**c**) and Fig. 7g (n=4). (**e**) Western blot analysis of individual PC-9 CDX tumours from (**c** and Fig. 7g) resected at endpoint (n=4). The expression of Ctl and CDA biodegraders is shown with the FLAG antibody. α-tubulin is the loading control. The star (*) indicates the top band obtained with the FLAG antibody, which is a non-specific band. Quantification of CDA protein level normalised to the loading control is shown on the right-hand side of each Western blot. Statistical significance in (**e**) was determined by a two-tailed unpaired Student's t-test (ns, not significant). Error bars indicate mean ± SEM (**a**, **c**, **d**, **e**). Source data are provided as a Source Data file.

Supplementary Table 1: Mass spectrometry collision parameters.

Coumpound	Parent (m/z)	Daughter (m/Z)	Cone (V)	Collision (V)
GEMCITABINE	263.8489	58.9814 (*)	90	34
GEMCITABINE	263.8489	94.7698	90	38
GEMCITABINE	263.8489	111.9407	90	18
2,2-Difluorodeoxyuridine	264.8900	69.9800 (*)	32	36
2,2-Difluorodeoxyuridine	264.8900	112.9200	28	22
2,2-Difluorodeoxyuridine	264.8900	223.9800	32	6
[13C, 15N2]-Gemcitabine	266.8900	58.9800	60	36
[13C, 15N2]-Gemcitabine	266.8900	97.2300	60	40
[13C, 15N2]-Gemcitabine	266.8900	114.9300 (*)	60	20

Quantification Trace (*)
