# Supplementary material for: A cytidine deaminase biodegrader sensitises multiple cancer types to deoxycytidine analogue-based chemotherapies
Source: Nat Commun. 2026 Oct 7;17:11402. doi: 10.1038/s41467-026-78315-0 (PMC13737281; doi:10.1038/s41467-026-78315-0)
Supplement: Supplementary file 2 [file 41467_2026_78315_MOESM2_ESM.pdf]

nature portfolio

Corresponding author(s): Pierre Cordelier and Nicolas Bery

Last updated by author(s): Aug 20, 2026

Reporting Summary

Nature Portfolio wishes to improve the reproducibility of the work that we publish. This form provides structure for consistency and transparency in reporting. For further information on Nature Portfolio policies, see our Editorial Policies and the Editorial Policy Checklist.

Statistics

For all statistical analyses, confirm that the following items are present in the figure legend, table legend, main text, or Methods section.

n/a	Confirmed	
[ ]	[x]	The exact sample size (*n*) for each experimental group/condition, given as a discrete number and unit of measurement
[ ]	[x]	A statement on whether measurements were taken from distinct samples or whether the same sample was measured repeatedly
[ ]	[x]	The statistical test(s) used AND whether they are one- or two-sided<br>*Only common tests should be described solely by name; describe more complex techniques in the Methods section.*
[x]	[ ]	A description of all covariates tested
[x]	[ ]	A description of any assumptions or corrections, such as tests of normality and adjustment for multiple comparisons
[ ]	[x]	A full description of the statistical parameters including central tendency (e.g. means) or other basic estimates (e.g. regression coefficient) AND variation (e.g. standard deviation) or associated estimates of uncertainty (e.g. confidence intervals)
[ ]	[x]	For null hypothesis testing, the test statistic (e.g. *F*, *t*, *r*) with confidence intervals, effect sizes, degrees of freedom and *P* value noted<br>*Give P values as exact values whenever suitable.*
[x]	[ ]	For Bayesian analysis, information on the choice of priors and Markov chain Monte Carlo settings
[x]	[ ]	For hierarchical and complex designs, identification of the appropriate level for tests and full reporting of outcomes
[x]	[ ]	Estimates of effect sizes (e.g. Cohen's *d*, Pearson's *r*), indicating how they were calculated

Our web collection on statistics for biologists contains articles on many of the points above.

Software and code

Policy information about availability of computer code

Data collection	Excel, Image Lab (version 6.1), Xcalibur (version 4.3.73.11), MassLynx (version 4.2).
Data analysis	The data analyses were performed using Image Lab (version 6.1, Bio-Rad), Prism v10.0 (GraphPad Software), FlowJo v10, Living Image (Revvity), Mascot (version 2.7.0), Proline (version 2.1.2) or MassLynx (version 4.2).

For manuscripts utilizing custom algorithms or software that are central to the research but not yet described in published literature, software must be made available to editors and reviewers. We strongly encourage code deposition in a community repository (e.g. GitHub). See the Nature Portfolio guidelines for submitting code & software for further information.

Data

Policy information about availability of data

All manuscripts must include a data availability statement. This statement should provide the following information, where applicable:

- Accession codes, unique identifiers, or web links for publicly available datasets
- A description of any restrictions on data availability
- For clinical datasets or third party data, please ensure that the statement adheres to our policy

The mass spectrometry proteomics data generated in this study have been deposited in the ProteomeXchange Consortium database via the PRIDE partner repository with the accession code PXD065390. The remaining data are available within the Article, Supplementary Information or Source Data file. Source data are provided with this paper.

Research involving human participants, their data, or biological material

Policy information about studies with human participants or human data. See also policy information about sex, gender (identity/presentation), and sexual orientation and race, ethnicity and racism.

Reporting on sex and gender	*Use the terms sex (biological attribute) and gender (shaped by social and cultural circumstances) carefully in order to avoid confusing both terms. Indicate if findings apply to only one sex or gender; describe whether sex and gender were considered in study design; whether sex and/or gender was determined based on self-reporting or assigned and methods used. Provide in the source data disaggregated sex and gender data, where this information has been collected, and if consent has been obtained for sharing of individual-level data; provide overall numbers in this Reporting Summary. Please state if this information has not been collected. Report sex- and gender-based analyses where performed, justify reasons for lack of sex- and gender-based analysis.*
Reporting on race, ethnicity, or other socially relevant groupings	*Please specify the socially constructed or socially relevant categorization variable(s) used in your manuscript and explain why they were used. Please note that such variables should not be used as proxies for other socially constructed/relevant variables (for example, race or ethnicity should not be used as a proxy for socioeconomic status). Provide clear definitions of the relevant terms used, how they were provided (by the participants/respondents, the researchers, or third parties), and the method(s) used to classify people into the different categories (e.g. self-report, census or administrative data, social media data, etc.) Please provide details about how you controlled for confounding variables in your analyses.*
Population characteristics	*Describe the covariate-relevant population characteristics of the human research participants (e.g. age, genotypic information, past and current diagnosis and treatment categories). If you filled out the behavioural & social sciences study design questions and have nothing to add here, write "See above."*
Recruitment	*Describe how participants were recruited. Outline any potential self-selection bias or other biases that may be present and how these are likely to impact results.*
Ethics oversight	*Identify the organization(s) that approved the study protocol.*

Note that full information on the approval of the study protocol must also be provided in the manuscript.

Field-specific reporting

Please select the one below that is the best fit for your research. If you are not sure, read the appropriate sections before making your selection.

- [x] Life sciences
- [ ] Behavioural & social sciences
- [ ] Ecological, evolutionary & environmental sciences

For a reference copy of the document with all sections, see nature.com/documents/nr-reporting-summary-flat.pdf

Life sciences study design

All studies must disclose on these points even when the disclosure is negative.

Sample size	This is only relevant to the mouse studies. No sample size calculations were performed. The number of mice used was based on our experience with the models. The sample sizes were sufficient as we reached statistical significance.
Data exclusions	One mouse of the vehicle control group injected with BxPC-3/CDA biodegrader and BxPC3/Ctl biodegrader, one mouse of each of the gemcitabine, doxycycline and gemcitabine/doxycycline groups injected with H441/CDA biodegrader was excluded from analysis due to lack of tumour development.
Replication	Reproducibility was verified by repeating the experiments. The number of repeats are stated in the figure legends.
Randomization	This is only relevant to the mouse experiments. Yes allocation was random.
Blinding	No blinding was used as the experimenter who planned the experiment also performed the experiment.

Reporting for specific materials, systems and methods

We require information from authors about some types of materials, experimental systems and methods used in many studies. Here, indicate whether each material, system or method listed is relevant to your study. If you are not sure if a list item applies to your research, read the appropriate section before selecting a response.

Materials & experimental systems

n/a	Involved in the study
	☒ Antibodies
	☒ Eukaryotic cell lines
☒	Palaeontology and archaeology
	☒ Animals and other organisms
☒	Clinical data
☒	Dual use research of concern
☒	Plants

Methods

n/a	Involved in the study
☒	ChIP-seq
	☒ Flow cytometry
☒	MRI-based neuroimaging

Antibodies

Antibodies used

CDA (1/1000, ABClonal Technology, Cat#A13959, lot 5500041544),
FLAG (1/2000, Sigma, Cat#F1804-200UG, lot 0000465990),
α-tubulin (1/2000, Abcam, Cat#ab4074, lot GR3402069-2),
pan-actin (1/50000, Millipore, Cat#MAB1501, lot 3800739),
CL-CASP3 (1/500, Cell Signaling Technology, Cat#9664S, lot 22),
CL-PARP (1/1000, Cell Signaling Technology, Cat#9541S, lot 21),
HSP90 (1/1000, Cell Signaling Technology, Cat#4874S, lot 6),
GAPDH (1/5000, Cell signaling Technology, Cat#5174S, lot 9),
PCNA (1/5000, Abcam, Cat #ab29, lot GR201287-6).
Secondary antibodies include anti-mouse-HRP (1/4000, Cell Signaling, Cat#7076S, lot 40), Anti-rabbit-HRP, 1/4000, Cell Signaling, Cat#7074S, lot 34)

Validation

All these antibodies have been validated for western blot use (see manufacturers website).
CDA: https://abclonal.com/catalog-antibodies/CDARabbitpAb/A13959
FLAG: hhttps://www.sigmaaldrich.com/FR/fr/product/sigma/f1804
Tubulin: https://www.abcam.com/en-us/products/primary-antibodies/alpha-tubulin-antibody-loading-control-ab4074
Actin: https://www.merckmillipore.com/FR/fr/product/Anti-Actin-Antibody-clone-C4,MM_NF-MAB1501
CL-CASP3: https://www.cellsignal.com/products/primary-antibodies/cleaved-caspase-3-asp175-5a1e-rabbit-monoclonal-antibody/9664
CL-PARP: https://www.cellsignal.com/products/primary-antibodies/cleaved-parp-asp214-antibody/9541
HSP90: https://www.cellsignal.com/products/primary-antibodies/hsp90-antibody/4874
GAPDH: https://www.cellsignal.com/products/primary-antibodies/gapdh-d16h11-rabbit-monoclonal-antibody/5174
PCNA: https://www.abcam.com/en-us/products/primary-antibodies/pcna-antibody-pc10-ab29
Anti-mouse HRP: https://www.cellsignal.com/products/secondary-antibodies/anti-mouse-igg-hrp-linked-antibody/7076
Anti-rabbit HRP: https://www.cellsignal.com/products/secondary-antibodies/anti-rabbit-igg-hrp-linked-antibody/7074

Eukaryotic cell lines

Policy information about cell lines and Sex and Gender in Research

Cell line source(s)

HeLa-S3 (ATCC, CCL-2.2), HEK293T (ATCC, CR-1573), MIA PaCa2 (ATCC, CRL-1420), AsPC-1 (ATCC, CRL-1682), BxPC-3 (ATCC, CRL-1687), Capan-2 (ATCC, HTB-80), H441 (ATCC, HTB-174), MRC-5 (ATCC, CCL-171) were obtained from ATCC.
PC-9, HCC4006 and BEAS-2B cell lines were a gift from Dr. Olivier Calvayrac and Rémi Gence (CRCT, Toulouse, France).

Authentication

Authentication was performed using short tandem repeat DNA profiling.

Mycoplasma contamination

All cell lines tested negative for mycoplasma contamination.

Commonly misidentified lines
(See ICLAC register)

None.

Animals and other research organisms

Policy information about studies involving animals; ARRIVE guidelines recommended for reporting animal research, and Sex and Gender in Research

Laboratory animals

For all xenograft experiments, 6 to 8-week-old female NSG mice (Charles River Laboratories) were used.

Wild animals

The study did not involve wild animals.

Reporting on sex

Female NSG mice were used for cell-derived xenograft studies. All animals used in the study were of one sex as gender was considered to have minimal impact on the outcome of this study.

Field-collected samples	The study did not involve field-collected samples.
Ethics oversight	Experimental procedures performed on mice were approved by the ethical committee of INSERM CREFRE US006 animal facility and authorized by the French Ministry of Research: APAFIS #57814-2025092411253403 v9. The maximal tumor size/burden authorised was 1000 mm3.

Note that full information on the approval of the study protocol must also be provided in the manuscript.

Plants

Seed stocks	*Report on the source of all seed stocks or other plant material used. If applicable, state the seed stock centre and catalogue number. If plant specimens were collected from the field, describe the collection location, date and sampling procedures.*
Novel plant genotypes	*Describe the methods by which all novel plant genotypes were produced. This includes those generated by transgenic approaches, gene editing, chemical/radiation-based mutagenesis and hybridization. For transgenic lines, describe the transformation method, the number of independent lines analyzed and the generation upon which experiments were performed. For gene-edited lines, describe the editor used, the endogenous sequence targeted for editing, the targeting guide RNA sequence (if applicable) and how the editor was applied.*
Authentication	*Describe any authentication procedures for each seed stock used or novel genotype generated. Describe any experiments used to assess the effect of a mutation and, where applicable, how potential secondary effects (e.g. second site T-DNA insertions, mosiacism, off-target gene editing) were examined.*

Flow Cytometry

Plots

Confirm that:

- [x] The axis labels state the marker and fluorochrome used (e.g. CD4-FITC).
- [x] The axis scales are clearly visible. Include numbers along axes only for bottom left plot of group (a 'group' is an analysis of identical markers).
- [x] All plots are contour plots with outliers or pseudocolor plots.
- [x] A numerical value for number of cells or percentage (with statistics) is provided.

Methodology

Sample preparation	Transfected or transduced cells were washed with PBS, trypsinised and resuspended in complete media before two washes of PBS. Cells were then either directly analysed at the flow cytometer or stained following the manufacturer protocol (Annexin V/PI staining).
Instrument	MACSQuant VYB Flow Cytometer (Miltenyi Biotec).
Software	FlowJo v10 Software was used to analyse the flow cytometry data.
Cell population abundance	No sorted cell population was used in this study. For our flow cytometry experiments, the analysis was performed on at least 5000 cells (in the final gate) per condition, except for the biodegraders screening that was done on at least 2000 cells per condition.
Gating strategy	Gating was done with SSC/FSC gates to exclude doublets and cell debris. Fluorescent cells or stained cells were gated to their related fluorescent gate (GFP/FITC, mCherry or PI) using unstained/untransfected cells. The gating strategy is shown for each type of flow cytometry experiment in Supplementary Fig. 1a, Supplementary Fig. 5g and Supplementary Fig. 8c.

- [x] Tick this box to confirm that a figure exemplifying the gating strategy is provided in the Supplementary Information.
