# Supplementary material for: A cytidine deaminase biodegrader sensitises multiple cancer types to deoxycytidine analogue-based chemotherapies
Source: Nat Commun. 2026 Oct 7;17:11402. doi: 10.1038/s41467-026-78315-0 (PMC13737281; doi:10.1038/s41467-026-78315-0)
Supplement: Supplementary file 3 [file 41467_2026_78315_MOESM3_ESM.pdf]

nature portfolio

Peer Review File

A cytidine deaminase biodegrader sensitises multiple cancer types to deoxycytidine analogue-based chemotherapies

Corresponding Author: Dr Nicolas Bery

This file contains all reviewer reports in order by version, followed by all author rebuttals in order by version.

Version 1:

Reviewer comments:

Reviewer #1

(Remarks to the Author)
This manuscript reports a protein-based degrader of cytidine deaminase, which is a well-characterized enzyme that converts cytosine-containing drugs to uracil metabolites that renders the drugs inactive. In fact, two clinically used drugs, cytarabine and gemcitabine, are rapidly inactivated by CDA, which underscores the utility for CDA inhibitors. Here, the authors screened a panel of chimeric E3 ubiquitin ligase proteins to find a protein that is competent to degrade a chimeric GFP-CDA protein. With SPOP identified as a "hit" ligase, the team then turned their attention to the discovery of an anti-CDA variable heavy domain of heavy chain-only antibody (VHH) using phage display. Three candidate polyclonal antibodies were identified, which were ultimately narrowed to two biodegraders called CDA 1 & CDA 2. Degradation profiling of the biodegraders (including Dox-inducible variants) narrowed the candidates to CDA 1 as the best biodegrader with good specificity by whole cell proteomics profiling. The ability of CDA 1 to deplete CDA was shown in several cell lines (primarily pancreas and lung cancer cell lines) and off-target profiling yielded favorable results with minimal effects in non-CDA-expressing cell lines. Importantly, CDA 1 was able to augment the antiproliferative potency of gemcitabine and promote entry of cells into apoptosis. With an eye towards clinical translation, the team then developed xenograft models and showed that CDA 1 can modulate tumor growth kinetics mice xenografted with their Dox-inducible CDA 1-expressing cell line, as well as in cells where CDA 1 is delivered as mRNA via VLPs. In both cases, the efficacy of gemcitabine was augmented, which supports their model of controlling CDA levels for therapeutic benefit.

Overall, this is a compelling study with minor weaknesses. The methodology is sound, the paper is clearly written, and the methods are well-described and comprehensive. A revision is required to address the following concerns prior to publication:

1. (Major Concern) The premise of this study is that biodegrader CDA 1 will augment the efficacy of cytosine-containing drugs by preventing (or slowing) their metabolic inactivation by CDA. Although the authors show that CDA 1 yields an enhanced therapeutic benefit for gemcitabine therapy, they do not prove the central hypothesis, which is that CDA 1 affects gemcitabine metabolism. The authors must demonstrate that CDA 1 expression affects C-to-U metabolism of gemcitabine by CDA. The CDA-mediated conversion of gemcitabine to its uracil metabolite has been studied previously by HPLC-MS. The authors should dose cells with several concentrations of gemcitabine, +/- CDA 1, and show that + CDA 1 yields lower levels of the 2',2'-difluorouridine metabolite. This would directly demonstrate a positive affect of gemcitabine metabolism.

2. The SPR sensorgrams for anti-CDA VHHs should be shown (at least in the SI). It would be useful to relay Kd to cellular efficacy.

3. The authors need to discuss why CDA biodegrader alone treatment provides a benefit for controlling tumor growth (see Figure 6A). Why does CDA treatment not look like vehicle-only treatment?

4. The two bands for the FLAG protein should be explained (Figure 5A western blot).

Reviewer #2

(Remarks to the Author)
This manuscript describes the development of an intracellular VHH–SPOP biodegrader targeting cytidine deaminase (CDA)

to sensitize cancer cells to deoxycytidine analogues (gemcitabine and cytarabine). The authors combine phage display, cell-based screening, proteomics validation, and in vivo xenograft models, and explore mRNA delivery via VLPs.

The work is technically sound, the screening strategy is well executed, and the target (CDA) is clinically relevant. However, the overall translational and conceptual advance is modest, and several important limitations, particularly related to delivery and in vivo relevance—are insufficiently discussed.

Major Comments
1. Magnitude of Chemosensitization Is Modest

While CDA degradation is robust in vitro, the observed IC50 shifts for gemcitabine and cytarabine appear relatively modest. For example, the IC50 shifts in the pancreatic and lung lines are all around 3x (e.g. AsPC-1: ~30 nM to ~10 nM). The shifts for the AML lines for cytarabine are a bit larger but still only 3 to 5x in magnitude. These are statistically significant but biologically modest shifts. Given that CDA degradation appears near-complete in vitro, these shifts likely represent the maximal achievable effect from CDA targeting alone. Accordingly, the authors should discuss:

(a) Whether such IC50 shifts would meaningfully change clinical dosing.

(b) Whether gemcitabine or cytarabine are dose-limited by toxicity in a way that a 3–5-fold shift would materially expand therapeutic index.

(c) Whether the benefit derives from shifting tumor sensitivity or reducing required systemic exposure.

- this discussion is essential for evaluating translational impact.

2. The manuscript does not address a key biological question: Must the biodegrader be expressed in every tumor cell for efficacy or is there a bystander effect? Given that CDA acts intracellularly on nucleoside analogues, one would predict that only cells expressing the biodegrader would be sensitized. There is unlikely to be meaningful paracrine amplification, unless the local concentration of drug in the tumor microenvironment increases significantly. Overall, this has major implications for delivery efficiency requirements. If near-uniform expression is required, the delivery hurdle becomes substantially higher. The authors should explicitly discuss this.

3. In Vivo Models: Modest Effect Size and Model Limitations

Although combination therapy shows tumor inhibition, several points require acknowledgment:

(a) PC-9 model

The PC-9 model appears relatively slow growing and highly gemcitabine-responsive. This represents a relatively low-bar efficacy model. The authors should acknowledge this limitation.

(b) Overall in vivo magnitude

Across models, the antitumor effects are present but modest. These results should be presented as proof-of-concept rather than near-therapeutic-level efficacy.

(c) Multiple injections required

Repeated intratumoral VLP injections are used. The feasibility and scalability of this approach are not discussed.

(d) Immunogenicity concerns. Specifically, repeated delivery of viral-like particles, intracellular engineered biodegrader constructs raises potential immunogenicity concerns that should be acknowledged. This should be discussed as well.

4. Intratumoral Injection Is a Major Translational Limitation

The most significant limitation of this study is that all in vivo delivery is performed via intratumoral injection.

This approach is not systemically deployable and does not address metastatic disease. The authors should explicitly state that these studies represent proof-of-concept only and that systemic, tumor-targeted delivery remains a major unsolved challenge. At present, the manuscript does not sufficiently acknowledge this limitation.

5. Conceptual Positioning: What Does This Paper Advance?

The manuscript appears to aim at two distinct advances:

(a) Advancement of biodegrader discovery methodology.

(b) Advancement of a therapeutic CDA-targeting strategy.

However, aside from the screening workflow (which is well executed and commendable), the paper does not meaningfully advance biodegrader engineering (no improvements in potency, half-life, modularity, or delivery chemistry). On the therapeutic front, delivery remains unresolved, limiting near-term translational relevance. Thus, the impact therefore appears modest relative to the stated scope.

Suggestions for Strengthening the Discussion

The authors should consider addressing:

1. mRNA optimization

Future work could include:

Codon optimization

UTR engineering

Nucleoside modifications

Structural mRNA optimization for longer half-life

2. Protein engineering

The degrader could potentially be improved through:

Affinity maturation of the VHH

Multivalent formats

Alternative E3 ligase selection

Half-life extension strategies

Intracellular stability engineering

A forward-looking discussion of these strategies would strengthen the manuscript.

Minor Comments

Page 4:
“They are known as bioprotect or biodegrader.”
- Should read: “These are known as bioPROTACs or biodegraders.”

Page 9:
“We then studied CDA degradation kinetic.”
- Should read: “degradation kinetics.”

Page 10:
“As previously performed with AsPC-1 cells,”
- A reference appears to be missing.

Overall Assessment

This is a careful and technically solid study demonstrating:

A well-designed screening pipeline for intracellular biodegraders.

Targeted degradation of CDA.

Modest but consistent chemosensitization in vitro and in vivo.

Proof-of-concept mRNA delivery via VLPs.

However:

The therapeutic impact is limited by delivery constraints.

The magnitude of sensitization is modest.

The in vivo approach is not clinically scalable.

The manuscript does not substantially advance biodegrader engineering or delivery technologies.

In its current form, the impact appears somewhat below the bar expected for Nature Communications, unless the discussion is strengthened to better frame the work as a methodological advance rather than a near-term therapeutic strategy.

Version 2:

Reviewer comments:

Reviewer #1

(Remarks to the Author)
My concerns from the prior review have been addressed. There is one minor typo that should be fixed. In the caption to Supporting Information Figure 5, the description for panel (C) refers to the data from panel (C), which is obviously incorrect. I believe this is referring to the data in panel (B).

Reviewer #2

(Remarks to the Author)
The authors have improved the manuscripts and addressed all of my concerns. I am now comfortable, recommending it for publication in Nature Communications

Open Access This Peer Review File is licensed under a Creative Commons Attribution 4.0 International License, which permits use, sharing, adaptation, distribution and reproduction in any medium or format, as long as you give appropriate credit to the original author(s) and the source, provide a link to the Creative Commons license, and indicate if changes were made.
In cases where reviewers are anonymous, credit should be given to 'Anonymous Referee' and the source.
The images or other third party material in this Peer Review File are included in the article's Creative Commons license, unless indicated otherwise in a credit line to the material. If material is not included in the article's Creative Commons license and your intended use is not permitted by statutory regulation or exceeds the permitted use, you will need to obtain permission directly from the copyright holder.
To view a copy of this license, visit https://creativecommons.org/licenses/by/4.0/

REVIEWER COMMENTS

Reviewer #1 (Remarks to the Author):

This manuscript reports a protein-based degrader of cytidine deaminase, which is a well-characterized enzyme that converts cytosine-containing drugs to uracil metabolites that renders the drugs inactive. In fact, two clinically used drugs, cytarabine and gemcitabine, are rapidly inactivated by CDA, which underscores the utility for CDA inhibitors. Here, the authors screened a panel of chimeric E3 ubiquitin ligase proteins to find a protein that is competent to degrade a chimeric GFP-CDA protein. With SPOP identified as a "hit" ligase, the team then turned their attention to the discovery of an anti-CDA variable heavy domain of heavy chain-only antibody (VHH) using phage display. Three candidate polyclonal antibodies were identified, which were ultimately narrowed to two biodegraders called CDA 1 & CDA 2. Degradation profiling of the biodegraders (including Dox-inducible variants) narrowed the candidates to CDA 1 as the best biodegrader with good specificity by whole cell proteomics profiling. The ability of CDA 1 to deplete CDA was shown in several cell lines (primarily pancreas and lung cancer cell lines) and off-target profiling yielded favorable results with minimal effects in non-CDA-expressing cell lines. Importantly, CDA 1 was able to augment the antiproliferative potency of gemcitabine and promote entry of cells into apoptosis. With an eye towards clinical translation, the team then developed xenograft models and showed that CDA 1 can modulate tumor growth kinetics mice xenografted with their Dox-inducible CDA 1-expressing cell line, as well as in cells where CDA 1 is delivered as mRNA via VLPs. In both cases, the efficacy of gemcitabine was augmented, which supports their model of controlling CDA levels for therapeutic benefit.

Overall, this is a compelling study with minor weaknesses. The methodology is sound, the paper is clearly written, and the methods are well-described and comprehensive. A revision is required to address the following concerns prior to publication:

1. (Major Concern) The premise of this study is that biodegrader CDA 1 will augment the efficacy of cytosine-containing drugs by preventing (or slowing) their metabolic inactivation by CDA. Although the authors show that CDA 1 yields an enhanced therapeutic benefit for gemcitabine therapy, they do not prove the central hypothesis, which is that CDA 1 affects gemcitabine metabolism. The authors must demonstrate that CDA 1 expression affects C-to-U metabolism of gemcitabine by CDA. The CDA-mediated conversion of gemcitabine to its uracil metabolite has been studied previously by HPLC-MS. The authors should dose cells with several concentrations of gemcitabine, +/- CDA 1, and show that + CDA 1 yields lower levels of the 2',2'-difluorouridine metabolite. This would directly demonstrate a positive affect of gemcitabine metabolism.

We have now assessed by Ultra Performance Liquid Chromatography (UPLC)-MS/MS the dFdU level from AsPC-1 cells supernatant treated with gemcitabine and expressing or not the CDA biodegrader. Our data show a significant decreased level of dFdU in cells expressing the CDA biodegrader (new Supplementary Figure 5b, c), suggesting a positive effect of the biodegrader on the gemcitabine metabolism. We added a comment in the result section as follow:

"We then assessed whether CDA biodegrader would directly impact gemcitabine metabolism by reducing its metabolic inactivation through CDA degradation. To this end, we quantified the inactive gemcitabine metabolite 2',2'-difluorodeoxyuridine (dFdU) level in AsPC-1 cells supernatant treated with gemcitabine and with or without doxycycline to induce the CDA biodegrader expression using Ultra Performance Liquid Chromatography (UPLC)-MS/MS. dFdU level is significantly decreased upon CDA biodegrader expression (Supplementary Fig. 5b, c), concomitant with CDA depletion (Supplementary Fig. 5c), suggesting that CDA biodegrader positively affects gemcitabine metabolism."

2. The SPR sensorgrams for anti-CDA VHHs should be shown (at least in the SI). It would be useful to relay Kd to cellular efficacy.

The SPR sensorgrams are displayed in Supplementary Fig. 2e.

3. The authors need to discuss why CDA biodegrader alone treatment provides a benefit for controlling tumor growth (see Figure 6A). Why does CDA treatment not look like vehicle-only treatment?

We have now discussed this point in the discussion section:

"Interestingly, the CDA biodegrader alone suppressed BxPC-3 CDX tumour growth (Fig. 6a, b), but had no effect on H441 CDX tumours (Fig. 6d, e), despite no measurable differences in 2D proliferation in either cell line (Fig. 4c, d). These findings suggest that the tumour growth inhibitory effects of CDA degradation may be context dependent, potentially reflecting cell line-specific differences in gene regulatory programmes that emerge under 3D growth conditions REF51. Similar context-specific dependencies have been described for KRAS signalling, where phenotypic effects are often more pronounced in 3D than in 2D assay systems and vary substantially across cell lines REF52, 53."

4. The two bands for the FLAG protein should be explained (Figure 5A western blot).

We have now added a comment in the result section regarding the double band, which is only observed in AML cells.

"Of note, a band with minor intensity was observed underneath the CDA biodegrader 1 expected band (Fig. 5a). This might result from a byproduct of the biodegrader, which is only seen in the AML cell lines."

Reviewer #2 (Remarks to the Author):

This manuscript describes the development of an intracellular VHH–SPOP biodegrader targeting cytidine deaminase (CDA) to sensitize cancer cells to deoxycytidine analogues (gemcitabine and cytarabine). The authors combine phage display, cell-based screening, proteomics validation, and in vivo xenograft models, and explore mRNA delivery via VLPs.

The work is technically sound, the screening strategy is well executed, and the target (CDA) is clinically relevant. However, the overall translational and conceptual advance is modest, and several important limitations, particularly related to delivery and in vivo relevance—are insufficiently discussed.

Major Comments

1. Magnitude of Chemosensitization Is Modest

While CDA degradation is robust in vitro, the observed IC50 shifts for gemcitabine and cytarabine appear relatively modest. For example, the IC50 shifts in the pancreatic and lung lines are all around 3x (e.g. AsPC-1: ~30 nM to ~10 nM). The shifts for the AML lines for cytarabine are a bit larger but still only 3 to 5x in magnitude. These are statistically significant but biologically modest shifts. Given that CDA degradation appears near-complete in vitro, these shifts likely represent the maximal achievable effect from CDA targeting alone. Accordingly, the authors should discuss:

(a) Whether such IC50 shifts would meaningfully change clinical dosing.

A 3-5-fold shift of the IC50 may not be enough to modify clinical dosing. Of note, linking *in vitro* IC50 to clinical dosing is a parameter difficult to directly assess. We would expect using the same dosing and therefore increase the drug efficacy in the tumours. Interestingly, this *in vitro* shift is enough to induce tumour regression in doxycycline-based CDX models (BxPC3 and H441), showing that even a modest shift is enough to potently chemosensitise tumours *in vivo*.

(b) Whether gemcitabine or cytarabine are dose-limited by toxicity in a way that a 3–5-fold shift would materially expand therapeutic index

If we do not expect a change of clinical dosing, therefore we might not expand the therapeutic index of these drugs that is actually narrow.

(c) Whether the benefit derives from shifting tumor sensitivity or reducing required systemic exposure.

In our case, as the clinical dosing of gemcitabine might not change, the benefit would rather derive from shifting the tumour sensitivity.

These three points are now added in the discussion section:

"From a translational perspective, we do not anticipate that clinical implementation of the CDA biodegrader would alter the dosing regimens of dC analogues or directly expand their therapeutic index. Rather, the principal benefit is expected to derive from enhanced tumour sensitivity to these agents."

- this discussion is essential for evaluating translational impact.

2. The manuscript does not address a key biological question: Must the biodegrader be

expressed in every tumor cell for efficacy or is there a bystander effect? Given that CDA acts intracellularly on nucleoside analogues, one would predict that only cells expressing the biodegrader would be sensitized. There is unlikely to be meaningful paracrine amplification, unless the local concentration of drug in the tumor microenvironment increases significantly. Overall, this has major implications for delivery efficiency requirements. If near-uniform expression is required, the delivery hurdle becomes substantially higher. The authors should explicitly discuss this.

This is indeed an important point to discuss. Actually, because the degradation is not complete *in vivo*, while it is *in vitro* with the PC-9 model, the biodegrader most likely has to be expressed in most tumour cells for efficacy. This highlights that improving the delivery efficiency and optimising the biodegrader mRNA/protein will be crucial in the future to enhance the *in vivo* response. We have now added a comment in the discussion regarding this point (which is also related to reviewer comment n°5):

"Although the *in vivo* data obtained in PC-9 tumours are promising, it is noteworthy that CDA degradation within tumours was incomplete, in contrast to the near-complete degradation observed *in vitro*. These findings suggest that robust expression of the biodegrader across the majority of tumour cells may be required to achieve near-complete target depletion, thereby maximising *in vivo* efficacy."

3. In Vivo Models: Modest Effect Size and Model Limitations

Although combination therapy shows tumor inhibition, several points require acknowledgment:

(a) PC-9 model

The PC-9 model appears relatively slow growing and highly gemcitabine-responsive. This represents a relatively low-bar efficacy model. The authors should acknowledge this limitation.

This limitation has been introduced in the discussion section:

"To fully evaluate the *in vivo* efficacy of the CDA biodegrader, it will be important to assess its delivery in additional tumour models that present greater biological challenges than the PC-9 CDX model, including higher growth rates and greater resistance to gemcitabine."

(b) Overall in vivo magnitude

Across models, the antitumor effects are present but modest. These results should be presented as proof-of-concept rather than near-therapeutic-level efficacy.

We agree that this work is a proof-of-concept study and we have now clearly indicated this in the abstract, introduction, results and discussion. Although, we did not present the *in vivo* results as near-therapeutic-level efficacy, we added "as proof-of-concept" in the result section of the VLPs for clarity:

"As proof-of-concept, we investigated an mRNA delivery method based on the FlashRNA® vector, which enables transient mRNA delivery via non-integrative VLPs[40]."

Regarding the antitumour effects, we acknowledge that these are modest in the PC-9 model (see our response to the previous reviewer comment) but the antitumour effects are compelling in the doxycycline-inducible CDX models, where we measured partial to near-total tumour regression. We amended the conclusion on the doxycycline-inducible models in the result section as follow:

"These doxycycline-inducible CDX models demonstrate a proof-of-concept that CDA biodegrader efficiently sensitises tumours to gemcitabine, inducing potent inhibition in the pancreatic cancer CDX model and near-complete regression in the lung cancer CDX model."

(c) Multiple injections required

Repeated intratumoral VLP injections are used. The feasibility and scalability of this approach are not discussed.

For clinical translation, decreasing the number of injections is mandatory as two injections weekly will not be feasible with patients. A maximum of one injection every two weeks could be done. Therefore, improving CDA biodegrader expression in tumours by improving the stability of either the mRNA or the biodegrader protein itself will be important (related to reviewer comment n°5). We added a comment in the discussion section:

"In the present study, VLPs were administered twice weekly in mice, a dosing frequency that is unlikely to be clinically practical. By contrast, an administration schedule of once every two weeks would be more feasible REF65. Achieving such a schedule will depend directly on the optimisation strategies outlined above to extend the half-life of both the delivered mRNA and the biodegrader protein."

(d) Immunogenicity concerns. Specifically, repeated delivery of viral-like particles, intracellular engineered biodegrader constructs raises potential immunogenicity concerns that should be acknowledged. This should be discussed as well.

The immunogenicity related to this delivery approach is indeed a potential concern that should be assessed in the future. We have now included a comment in the discussion on the immunogenicity issue related to this delivery method and cargo:

"Importantly, repeated dosing also raises potential immunogenicity concerns related to both the delivery vehicle and its cargo. Although IT delivery may partially mitigate this risk, immunogenicity will require careful evaluation before clinical translation."

4. Intratumoral Injection Is a Major Translational Limitation

The most significant limitation of this study is that all in vivo delivery is performed via intratumoral injection.

This approach is not systemically deployable and does not address metastatic disease. The authors should explicitly state that these studies represent proof-of-concept only and that

systemic, tumor-targeted delivery remains a major unsolved challenge. At present, the manuscript does not sufficiently acknowledge this limitation.

We acknowledge that IT injection is a limitation as patients with metastasis cannot be selected with this route of injection. Nevertheless, IT injection presents several advantages such as high therapeutic index and low toxicity (PMID: 34006998). It could be used in patients without metastasis, which still represent 30-40% of patients with pancreatic cancer for instance. Furthermore, IT route of injection has been previously used in a gene therapy clinical trial led by clinicians from our group (phase I and II, NCT02806687 and PMID: 25586689), showing its feasibility in patients with pancreatic cancer (but also in clinical trials led by other groups, e.g. PMID: 29801474). To reach a higher number of patients, tumour-targeted delivery should be implemented but as indicated by the reviewer, it is at the moment an unsolved challenge. We have now acknowledged this limitation in the discussion section:

"Once both the CDA biodegrader and the delivery method have been optimised, additional work will be required before advancing this therapeutic strategy towards clinical application. In this proof-of-concept study, VLPs were administered intratumourally in mice, as achieving systemic tumour-specific delivery remains a major challenge. Although IT injection has an important clinical limitation, *i.e.* not applicable in patients with metastatic disease, it also offers several advantages, including a high therapeutic index and low systemic toxicity[65]. Notably, IT injection has previously been used in a clinical gene therapy trial (phase I/II, NCT02806687)[66], supporting the clinical feasibility of this route of administration in pancreatic cancer."

5. Conceptual Positioning: What Does This Paper Advance?

The manuscript appears to aim at two distinct advances:

(a) Advancement of biodegrader discovery methodology.

(b) Advancement of a therapeutic CDA-targeting strategy.

However, aside from the screening workflow (which is well executed and commendable), the paper does not meaningfully advance biodegrader engineering (no improvements in potency, half-life, modularity, or delivery chemistry). On the therapeutic front, delivery remains unresolved, limiting near-term translational relevance. Thus, the impact therefore appears modest relative to the stated scope.

Following reviewers' comments, we have now acknowledged in the manuscript that this is a proof-of-concept study in which further work will be required before advancing CDA biodegrader towards clinical application.

We believe that this study advances the biodegrader field because: (i) it provides the first described CDA (bio)degrader with a high specificity for its target, (ii) it shows the feasibility to deliver CDA biodegrader with VLPs *in vitro* and *in vivo* (to our knowledge, it is the first time that VLPs have been used to deliver mRNA encoding biodegraders), (iii) it enables to achieve a

near-complete CDA degradation *in vitro*. This latter point also suggests that, despite further work should be done to improve the CDA biodegrader stability/potency, the major improvement to be done in the future should be at the mRNA level, more than on the biodegrader engineering side.

Suggestions for Strengthening the Discussion

The authors should consider addressing:

1. mRNA optimization

Future work could include:

Codon optimization

UTR engineering

Nucleoside modifications

Structural mRNA optimization for longer half-life

2. Protein engineering

The degrader could potentially be improved through:

Affinity maturation of the VHH

Multivalent formats

Alternative E3 ligase selection

Half-life extension strategies

Intracellular stability engineering

A forward-looking discussion of these strategies would strengthen the manuscript.

We have now added a discussion of these optimisation strategies (at the mRNA and protein levels). Regarding the affinity maturation, it is important to note that the anti-CDA VHH affinity is already in the pM range, which is relatively high for a VHH. Hence, improving its K_D might be challenging. Furthermore, different articles showed that a high binder affinity does not always translate to an improved degradation efficacy (PMID: 40542219 and PMID: 38746666). The improvement of the biodegrader should rather be focused on other parameters such as the protein stability or biodegrader potency that we have now discussed:

“Accordingly, further optimisation of both the CDA biodegrader and its delivery strategy will be necessary to improve biodegrader stability and expression at both the mRNA and protein levels. At the mRNA level, strategies may include codon optimisation of the coding sequence and modification of the 5′ and/or 3′ untranslated regions (UTRs) to improve translational efficiency REF60, 61. mRNA stability may be further increased through nucleoside modifications (e.g. substitution of uridine with pseudouridine) and cap structure engineering (e.g. Cap 1 or Cap 2 structures) REF60, 61. Finally, other RNA platforms providing enhanced and prolonged protein expression, such as self-amplifying RNA REF62 or circular RNA REF63, could be explored. Collectively, these approaches are expected to enhance and sustain biodegrader expression.

Although we demonstrate that the CDA biodegrader remains expressed in cells for at least 2 days following delivery, its intracellular protein stability may be further improved through lysine residue mutagenesis REF44 and/or the introduction of stabilising mutations within the VHH framework REF46. These approaches have previously been shown to reduce intracellular proteolysis of a KRAS biodegrader REF44 and to enhance the stability of intracellular VHHs REF46, respectively. In parallel, even though we demonstrate a near-complete CDA degradation in vitro, degradation potency may be further improved through several complementary strategies. For example, a multivalent CDA biodegrader could be developed, as this format has previously been shown to maximise the degradation efficacy of SHP2 biodegraders REF64. Increased potency may also be achieved through the identification of alternative E3 ligases with greater activity than SPOP. Finally, the delivery strategy itself could be further refined, either by increasing the concentration of VLPs administered intratumourally or by implementing alternative delivery platforms such as nanoparticles.”

Minor Comments Page 4:

“They are known as bioprotect or biodegrader.”

- Should read: “These are known as bioPROTACs or biodegraders.”

Page 9:

“We then studied CDA degradation kinetic.”

- Should read: “degradation kinetics.”

Page 10:

“As previously performed with AsPC-1 cells,”

- A reference appears to be missing.

We have now corrected these minor comments in the manuscript.

Overall Assessment

This is a careful and technically solid study demonstrating:

A well-designed screening pipeline for intracellular biodegraders.

Targeted degradation of CDA.

Modest but consistent chemosensitization in vitro and in vivo.

Proof-of-concept mRNA delivery via VLPs.

However:

The therapeutic impact is limited by delivery constraints.

The magnitude of sensitization is modest.

The in vivo approach is not clinically scalable.

The manuscript does not substantially advance biodegrader engineering or delivery technologies.

In its current form, the impact appears somewhat below the bar expected for Nature Communications, unless the discussion is strengthened to better frame the work as a methodological advance rather than a near-term therapeutic strategy.

We thank the reviewer for acknowledging that this study is well designed and technically solid with a commendable screening workflow of intracellular biodegraders. We also thank the reviewer comments that have helped us to better highlight the methodological advance demonstrated by this proof-of-concept study as well as the potential strategies to improve its translatability towards future clinical applications.

REVIEWER COMMENTS

Reviewer #1 (Remarks to the Author):

My concerns from the prior review have been addressed. There is one minor typo that should be fixed. In the caption to Supporting Information Figure 5, the description for panel (C) refers to the data from panel (C), which is obviously incorrect. I believe this is referring to the data in panel (B).

We have now corrected this typo in the figure legend.

Reviewer #2 (Remarks to the Author):

The authors have improved the manuscripts and addressed all of my concerns. I am now comfortable, recommending it for publication in Nature Communications
